# Hybridized Charge‐Transfer Window within a Fully Conjugated Multi‐Resonance Thermally Activated Delayed Fluorescence Framework for Ultrafast Reverse Intersystem Crossing and High‐Efficiency in Deep‐Blue Organic Light‐Emitting Diodes

**DOI:** 10.1002/adma.72861

**Published:** 2026-03-24

**Authors:** Uisung Lee, Kyungwoo Jeong, Sunwoo Kang, Jun Yeob Lee

**Affiliations:** ^1^ Department of Display Engineering Sungkyunkwan University Suwon Gyeonggi‐do South Korea; ^2^ Department of Chemistry Dankook University Cheonan Chungcheongnam‐do Republic of Korea; ^3^ School of Chemical Engineering Sungkyunkwan University Suwon Gyeonggi Republic of Korea; ^4^ SKKU Institute of Energy Science and Technology Sungkyunkwan University Suwon Gyeonggi Republic of Korea

**Keywords:** blue OLED, HCT, high efficiency, MR‐TADF, RISC

## Abstract

Multi‐Resonance (MR) thermally activated delayed fluorescence (TADF) materials, featuring narrow band emission and high efficiency, are being utilized in blue organic light‐emitting diode (OLED) applications. However, compared to conventional donor–acceptor (D–A) type TADF emitters, these molecules exhibit relatively large singlet‐triplet energy gaps and slow reverse intersystem crossing (RISC) rates, indicating the need for further research to overcome these limitations. Herein, we designed deep blue MR‐TADF molecules by integrating intramolecular short‐range charge transfer and long‐range charge transfer to minimize the singlet‐triplet energy gap and accelerate the RISC rate through hybridized excited states maintaining a narrow emission bandwidth. Compared with conventional MR‐TADF molecules emitting only by short‐range charge transfer, the new MR‐TADF molecule is differentiated in that intramolecular long‐range charge transfer within the polyaromatic hydrocarbon framework additionally contributes to the emission process along with short‐range charge transfer for accelerated RISC. As a result, the ***bf*DOB‐BN2** MR‐TADF emitter exhibited narrow blue emission at 447 nm with a narrow full width at half maximum (FWHM) of 20 nm, a small singlet‐triplet energy gap of 0.04 eV, and an ultrafast RISC rate of 2.1 × 10^6^ s^−^^1^. In a blue TADF OLED device, the new TADF molecule showed a high external quantum efficiency of 37.5% with color coordinates (0.139 and 0.065).

## Graphical abstract

A hybridized charge‐transfer window combining short‐range and long‐range charge transfer is realized within a fully conjugated multi‐resonance thermally activated delayed fluorescence framework. The resulting excited‐state hybridization minimizes the singlet–triplet energy gap (0.04 eV) and enables ultrafast reverse intersystem crossing (*k_rISC_* = 2.1 × 10^6^ s^−^^1^), leading to an exceptionally high external quantum efficiency of 37.5% with narrow deep‐blue emission approaching the BT.2020 standard.

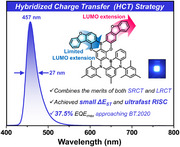

## 1 Introduction

Thermally activated delayed fluorescence (TADF) emitters enable harvesting of all triplet excitons via reverse intersystem crossing (RISC) mechanism, thereby achieving up to 100% internal quantum efficiency [1, 2]. This allows for high external quantum efficiency (EQE) without expensive rare metals such as platinum (Pt) or iridium (Ir), highlighting the significance of TADF in both academic, and industrial fields [3, 4, 5, 6].

Conventional TADF materials adopt a twisted donor–acceptor (D–A) architecture to spatially separate the highest occupied molecular orbital (HOMO) and lowest unoccupied molecular orbital (LUMO). The D‐A type TADF molecules effectively minimize the singlet‐triplet energy gap (ΔE_ST_) and facilitate rapid RISC [7, 8]. However, the D–A structures linked through a single bond inherently generate manifold rotational and vibrational states, which in turn induce pronounced structural relaxation in the excited state. Consequently, the D–A type TADF emitters suffer from a large Stokes shift and a broad emission bandwidth, both of which are detrimental to device lifetime and color purity [9, 10, 11, 12].

In 2016, the Hatakeyama group reported DABNA (5,9‐diphenyl‐5,9‐diaza‐13b‐boranaphtho[3,2,1‐de] anthracene), which exhibits a narrow full width at half maximum (FWHM) and a high radiative decay rate through multiple resonance (MR) effect. It induces atomic‐level spatial separation of the frontier orbitals, in which the HOMO is localized on electron‐rich nitrogen (N) atoms and the LUMO on electron‐deficient boron (B) atoms, with both orbitals alternately distributed at the ortho‐ and para‐positions of the polycyclic aromatic hydrocarbon (PAH) framework. This unique electronic configuration, within a rigid π‐conjugated framework, introduces short‐range charge transfer (SRCT), resulting in high photoluminescence quantum yield (PLQY), and ultranarrow FWHM [13]. However, despite providing a small ΔE_ST_, this molecular design still suffers from relatively slow RISC compared with typical D–A‐type TADF systems because the singlet and triplet states originate from closely similar SRCT‐based orbitals, leading to suppressed spin–orbit coupling according to El‐Sayed's rule [14, 15, 16]. Consequently, this leads to severe efficiency roll‐off at high luminance and accelerates material degradation induced by high‐energy triplet states [17, 18, 19, 20, 21]. Recently, several materials with exceptionally high photophysical properties and excellent device performance have been reported based on multi‐boron–extended MR core strategies [22, 23]. However, this molecular expansion approach inevitably increases synthetic complexity, leading to the formation of impurities from multiple ring systems during cyclization and ultimately lowering the overall yield. Moreover, the excessive increase in molecular weight and planarity markedly raises the sublimation or deposition temperature, and the resulting thermal stress can induce degradation during processing [24, 25]. Therefore, despite their outstanding intrinsic properties, such materials face significant limitations for practical commercialization.

Another approach involves the peripheral introduction of donor and/or acceptor units to the MR core, enabling the mixing of long‐range charge transfer (LRCT) character. This strategy can effectively reduce ΔE_ST_ and promote a faster RISC process. However, as previously discussed, the incorporation of LRCT character often causes both spectral broadening and a large Stokes shift, compromising color purity and device lifetime [26, 27, 28, 29, 30, 31, 32, 33]. Additionally, the intrinsic low singlet energy of the charge transfer (CT) state in D–A systems causes a redshift in emission, making it difficult to achieve deep‐blue emission [34, 35, 36, 37]. Therefore, the development of deep‐blue emitters that can simultaneously harness LRCT to facilitate small ΔE_ST_ and fast RISC while maintaining narrow emission bandwidth, remains a critical research challenge.

Herein, we synthesized three DABNA‐derived isomeric emitters, ***bf*DOB‐BN1** (14,18‐dimesityl‐16‐methyl‐14,18‐dihydro‐7,12,23‐trioxa‐14,18‐diaza‐4b,22b‐diboraindeno[1,2‐a]dinaphtho[1,2,3‐fg:1',2',3'‐jk]pentacene), ***bf*DOB‐BN2** (4,26‐dimesityl‐2‐methyl‐4,26‐dihydro‐6,11,15,21‐tetraoxa‐4,26‐diaza‐13b,21c‐diboraindeno[1,2‐a]indeno[1',2':6,7]naphtho[1,2,3‐fg]naphtho[1,2,3‐jk]pentacene), and ***bf*DOB‐BN3** (11,15‐dimesityl‐13‐methyl‐11,15‐dihydro‐6,17,23,26‐tetraoxa‐11,15‐diaza‐6c,24b‐diboraindeno[1,2‐b]indeno[1',2':6,7]naphtho[1,2,3‐fg]naphtho[1,2,3‐jk]pentacene) by combining a DABNA core with a benzofuran‐fused DOBNA (5,9‐dioxa‐13b‐boranaphtho[3,2,1‐de]anthracene) framework to enable both intramolecular SRCT and LRCT within PAH framework without introducing any peripheral donor or acceptor units. Only two boron atoms embedded in the main skeleton were employed to minimize the molecular weight for practical applications. The three isomers were differentiated in that the benzofuran was fused to the DOBNA unit through different positions of it. The management of the fused position of benzofuran to the DOBNA framework controlled the molecular orbital distribution and contribution of LRCT to the MR‐TADF emission. The fusion of benzofuran with oxygen oriented to the meta position of boron induced LUMO extension to the benzofuran and accompanying LRCT character, while that with oxygen oriented to the para position of boron did not change the orbital distribution. Among the three isomeric emitters, ***bf*DOB‐BN2** demonstrated a small ΔE_ST_ of 0.04 eV, and a fast RISC rate of 2.1 × 10^6^ s^−^^1^ through a well‐balanced LRCT and SRCT character within the MR core inducing hybridized charge‐transfer (HCT) emission properties. This resulted in a narrow FWHM of 20 nm and deep‐blue emission at 447 nm, even without the incorporation of peripheral substitution. The corresponding device achieved a high EQE of 37.5% and improved EQE roll‐off performance in the deep‐blue region with a CIEy of 0.065. As a result, by incorporating a benzofuran fused DOBNA unit into SRCT–based DABNA derivatives, the LRCT contribution can be finely tuned, leading to HCT emission characteristics, and outlining a new molecular design strategy that enables dramatic RISC acceleration while maintaining an exceptionally narrow FWHM. The EQE and RISC rate recorded in this work are one of the state‐of‐the‐art values reported in the one‐ and two‐boron‐based MR‐TADF emitters.

## 2 Results and Discussion

### 2.1 Molecular Design and Theoretical Calculation

**FIGURE 1:**
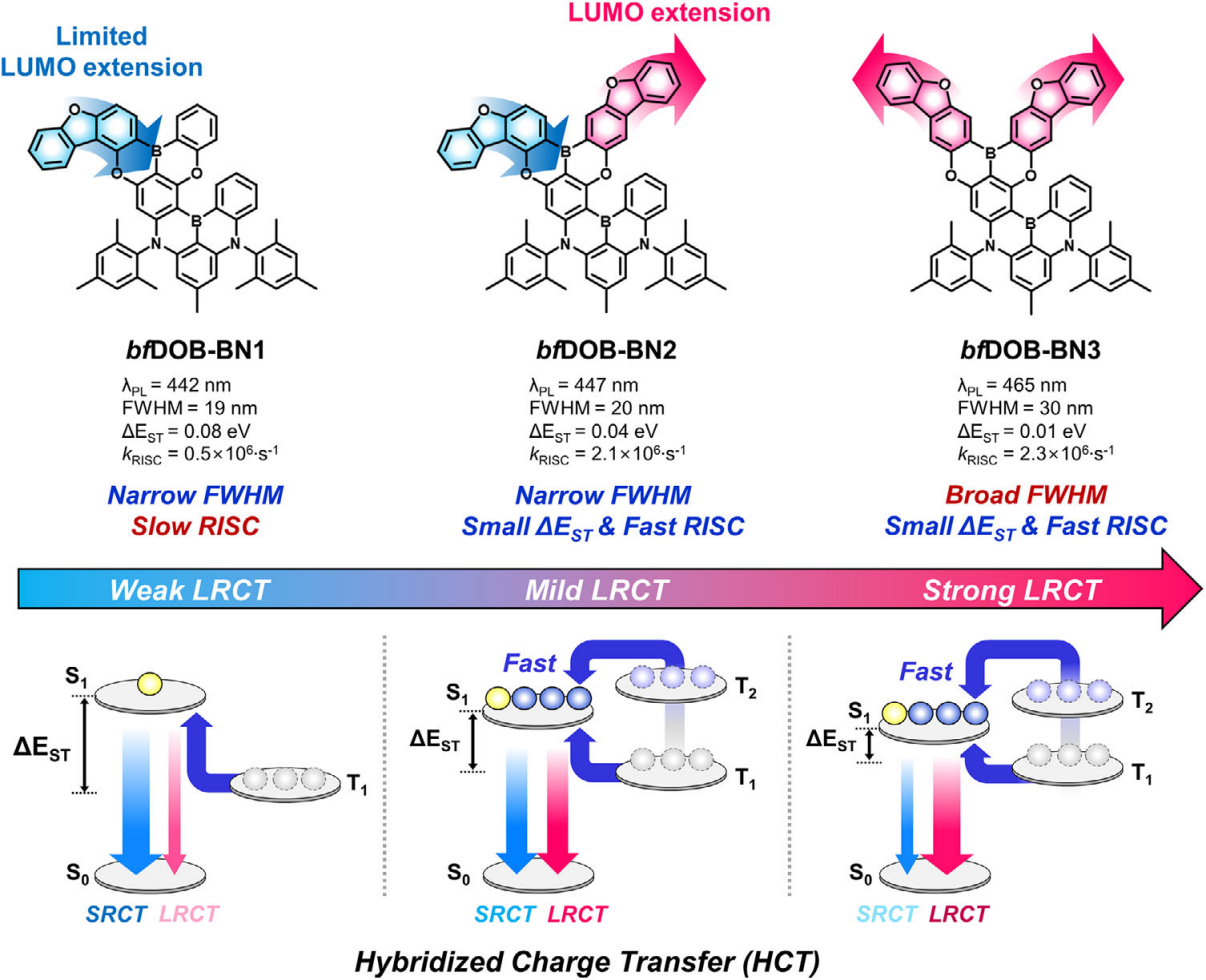
Molecular design concept of benzofuran‐extended MR‐TADF emitters.

In the molecular design of the blue MR‐TADF materials emitting both by SRCT and LRCT, a benzofuran‐fused DOBNA unit within a PAH molecular framework was employed to manage molecular orbital distribution and to maintain the narrow emission characteristics associated with the SRCT character of the DABNA core while incorporating LRCT emission. A DABNA merged with a DOBNA unit was the main skeleton of the MR‐TADF emitters and only the DOBNA unit was fused with benzofuran units to finely tune the CT strength. One or two benzofuran units were fused with the DOBNA core and the fusion position of benzofuran was changed to control the LRCT character of the emitters. In the case of ***bf*DOB‐BN1**, one benzofuran unit was fused in the DOBNA‐based PAH structure and the oxygen was positioned at the para position (para‐oriented benzofuran) relative to the boron of DOBNA. ***bf*DOB‐BN2** has an additional benzofuran unit fused to the DOBNA unit and the oxygen of the extra benzofuran was oriented to the meta position (meta‐oriented benzofuran) to the boron of DOBNA. Whereas, ***bf*DOB‐BN3** has two benzofuran units symmetrically fused to the DOBNA unit through meta‐oriented oxygen and boron atoms. It is expected that the change of the orientation of benzofuran may affect the molecular orbital distribution and degree of LRCT. The design motivation of the emitters is explained in Figure 1.

**FIGURE 2:**
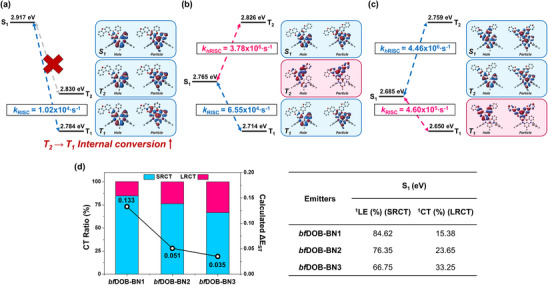
The spatial distribution of hole‐ and electron‐ NTOs in the S_1_, T_1_, and T_2_ states for (a) ***bf*DOB‐BN1**, (b) ***bf*DOB‐BN2**, and (c) ***bf*DOB‐BN3**. (d) Calculated ΔE_ST_ evaluated with the STEOM‐DLPNO‐CCSD/SVP basis set at the LC‐ω*HPBE/6‐31G* level of theory and the corresponding CT ratio of emitters estimated from intra‐fragment charge transfer (IFCT) analysis.

Density functional theory (DFT) calculations were conducted to compare the pivotal parameters of the photophysical properties in ***bf*DOB‐BN1**, ***bf*DOB‐BN2**, and ***bf*DOB‐BN3**, as implemented in the suite of Gaussian 16 program [38]. To understand the electronic properties of these materials, LC‐ωhPBE functional [39, 40, 41] was used with the 6–31G* basis set. The optimal ω values were determined by minimizing J^2^(ω) (Figure S1). TDDFT calculations in conjunction with Tamm‐Dancoff approximation (TDA) [42] were performed to gain the optimized structures and their corresponding energy levels in the singlet and triplet excited states (S_1_ and T_1_). The optimized structures and their corresponding natural transition orbitals (NTOs) in S_1_, T_1_, and T_2_ states are depicted in Figure 2.

The hole‐and electron‐NTOs of S_1_, T_1_, and T_2_ states are similarly distributed on the molecular structure of ***bf*DOB‐BN1**. In detail, the hole‐NTOs predominantly lie on the DABNA core unit, but electron‐NTOs are mainly distributed on the DABNA‐core unit and partially spread on the DOBNA core unit. From these results, it is expected that the transition characteristics of the three excited states can be assigned as a mixing of strong SRCT and weak LRCT. In ***bf*DOB‐BN2**, the S_1_ and T_1_ states show a similar spatial distribution of NTOs to ***bf*DOB‐BN1**. However, the NTOs of the T_2_ state are quite different from both NTOs mainly spreading on the benzofuran‐fused DOBNA unit. Interestingly, ***bf*DOB‐BN3** shows the distinct spatial distributions of both NTOs, compared to other compounds. Both NTOs of the S_1_ state are entirely spread on the DABNA‐core and DOBNA core units while those of the T_1_ state are mainly distributed on the benzofuran‐fused DOBNA unit. In addition, hole‐NTO of T_2_ state mainly delocalizes on the DABNA core unit and partially populates on the DOBNA core, but electron‐NTOs spread on the DABNA and DOBNA cores. These results indicate that the presence of benzofuran units and their position play a pivotal role in perturbing the electronic structures in the excited states.

To quantitatively obtain the excitation characteristics of these complexes, the IFCT analyses were performed using Multiwfn software (Figure 2d) [43]. As a result, the contribution of the SRCT on the S_1_ state is calculated to be 84.62%, 76.35%, and 66.75%, respectively, corresponding to the ***bf*DOB‐BN1**, ***bf*DOB‐BN2**, and ***bf*DOB‐BN3**. On the contrary, the calculated LRCT contributions on S_1_ states are 15.38%, 23.65%, and 33.25%, respectively. From these results, it is evident that the introduction of benzofuran units to extend the π‐conjugation suppresses the SRCT character and enhances the LRCT. Furthermore, it is expected that the HCT character may reinforce in the order of ***bf*DOB‐BN1**< ***bf*DOB‐BN2**< ***bf*DOB‐BN3** due to the increase of the LRCT.

The calculated S_1_, T_1_, and T_2_ energies computed by STEOM‐DLPNO‐CCSD calculations are listed in Table S1. All energies were obtained at the optimized molecular structures of S_1_, T_1_, and T_2_ states from TDDFT calculation with LC‐ω*hPBE/6‐31G* level of theory. The calculated S_1_ energy, T_1_ energy, and their corresponding ΔE(S_1_‐T_1_) are in quantitative agreement with experiments.

From these results, it can be understood that the extensive π‐conjugation induced by the addition of the benzofuran moiety leads to a decrease in both excited state energies ΔE(S_1_‐T_1_) values. This result indicates that a design strategy to extend π‐conjugation plays an advantageous role in enhancing the conversion efficiency of the triplet exciton into the singlet exciton. Based on these computed parameters, the reverse intersystem crossing rate (*k_rISC_*) from T_1_ to S_1_ states is calculated to be 1.017 × 10^4^, 6.551 × 10^4^, and 4.602 × 10^5^ *s^−1^*, respectively, which correspond to faster *k_rISC_* in the order of ***bf*DOB‐BN1**< ***bf*DOB‐BN2**< ***bf*DOB‐BN3**.

To check the existence of the spin‐flip transition from high‐lying to low‐lying internal states, we further consider the spin‐flip transition from T_2_ and S_1_ states. Except for ***bf*DOB‐BN1**, T_2_ energies of ***bf*DOB‐BN2** and ***bf*DOB‐BN3** are endothermically positioned relative to their S_1_ energies. Based on the results of NTOs, the *k_hrISC_* of ***bf*DOB‐BN1** from T_2_ to S_1_ state is expected to be forbidden due to the activation of internal conversion. In contrast, *k_hrISC_* may be allowed in ***bf*DOB‐BN2** and ***bf*DOB‐BN3** since the vibronic coupling between T_1_ and T_2_ states is expected to be weak due to the non‐overlapping of two transition orbitals, thereby, *k_hrISC_* is likely to be activated in these compounds. The calculated *k_hrISC_* values of ***bf*DOB‐BN2** and ***bf*DOB‐BN3** are 3.775 × 10^6^ and 4.455 × 10^6^ *s^−1^*. The results of the spin‐flip transition behaviors and electronic structures of the excited states lead us to expect that ***bf*DOB‐BN1** solely reveals the thermal activation‐dependent exciton conversion while ***bf*DOB‐BN2** and ***bf*DOB‐BN3** exhibit the simultaneous spin‐flip transitions via thermal activation and non‐thermal activation.

The three emitters were synthesized according to the procedures outlined in Scheme S1. They shared the same intermediate A and different building blocks for the benzofuran‐fused DOBNA were used for each compound (Scheme S2). Intermediate A was constructed via successive Buchwald–Hartwig aminations, while intermediates B1, B2, and B3 were obtained through nucleophilic aromatic substitution. The two intermediates were coupled by a Buchwald–Hartwig amination to yield the corresponding AB1, AB2, and AB3 precursors. Subsequent one‐pot borylation with boron triiodide furnished the final compounds. All intermediates were verified by ^1^H‐nuclear magnetic resonance (NMR) and mass spectrometry, and the final products were further confirmed by ^13^C‐NMR and high‐resolution mass spectrometry. For ***bf*DOB‐BN2** and ***bf*DOB‐BN3**, ^1^H–^1^H COSY (correlation spectroscopy) analysis was additionally performed to further confirm the molecular structures (Figures S2–S23). Synthetic details and characterization data are provided in the Supporting Information.

### 2.2 Photophysical Properties

**FIGURE 3:**
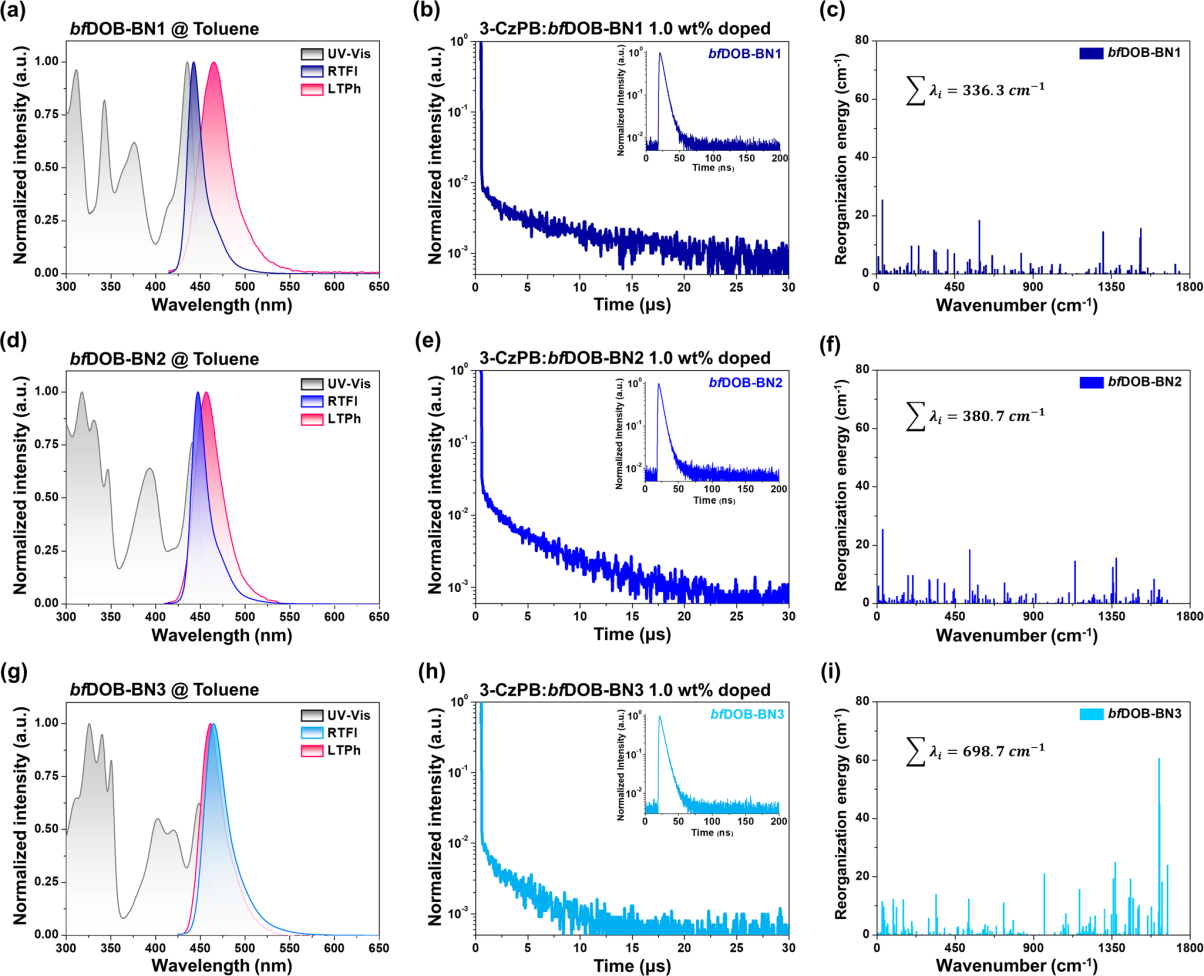
UV–vis and PL spectra (10^−^^5^ m in toluene), normalized transient PL decay of the delayed component (1.0 wt.% doped in 3‐CzPB film) and reorganization energy (λ) as a function of vibrational wavenumber for ***bf*DOB‐BN1**, (a–c), ***bf*DOB‐BN2** (d–f) and ***bf*DOB‐BN3** (g–i). RTFl and LTPh denote room‐temperature fluorescence and low‐temperature phosphorescence. Insets in (b), (e), and (h) represent the prompt components.

To investigate the photophysical properties of the synthesized emitters, UV–vis absorption, and photoluminescence (PL) measurements were conducted in dilute toluene solution (10^−^^5^ m) (Figure 3; Figure S24). All three compounds displayed comparable absorption profiles, with distinct bands appearing in the regions of 300–350, 350–400, and approximately 430–450 nm. The absorption band between 300 and 350 nm is assigned to the *π*–π* transition of the extended benzofuran moiety within the DOBNA core, while the features in the 350–400 nm region correspond to the n–π* transitions of the ***bf*DOB‐BN1**, ***bf*DOB‐BN2**, and ***bf*DOB‐BN3** monomers. The sharp absorption bands centered at 435 nm for ***bf*DOB‐BN1**, 441 nm for ***bf*DOB‐BN2**, and 449 nm for ***bf*DOB‐BN3** are attributed to SRCT transitions induced by the MR effect [44, 45, 46]. PL spectra revealed that ***bf*DOB‐BN1** exhibited a moderately blueshifted emission peak at 442 nm with a narrow FWHM of 18 nm, which is attributed to the electron‐donating effect of the oxygen atom at the para‐position relative to the boron center. Negligible orbital delocalization toward the benzofuran moiety was observed in the molecular simulation of ***bf*DOB‐BN1**, yielding a high triplet energy of 2.66 eV and the largest ΔE_ST_ of 0.08 eV among the three emitters. This is attributed to the weakened electron‐deficient character of the DOBNA unit caused by the electron‐donating benzofuran substituent which suppresses LRCT character. The small Stokes shift of 6 nm of ***bf*DOB‐BN1** reflects the rigidity of the molecular structure. Compared with ***bf*DOB‐BN1**, ***bf*DOB‐BN2** showed an emission peak at 447 nm with a narrow FWHM of 20 nm and a small Stokes shift of 6 nm. Although a slight redshift and broadening of the emission spectrum were observed, the additional meta‐oriented benzofuran maintained the deep blue narrowband emission of the main backbone structure. One noticeable effect of the meta‐oriented benzofuran is the decrease of ΔE_ST_ from 0.08 eV (***bf*DOB‐BN1**) to 0.04 eV (***bf*DOB‐BN2**) because of slightly lowered singlet energy while keeping the triplet energy. As predicted by molecular simulation, the additional meta‐oriented benzofuran induced LRCT character, decreasing the singlet energy and ΔE_ST_ at the same time. In the case of ***bf*DOB‐BN3** with two meta‐oriented fused benzofurans, a substantially red‐shifted emission peak at 465 nm along with a Stokes shift of 16 nm and a broadened FWHM of 30 nm was observed due to strong LRCT character by two benzofuran units. Although a very small ΔE_ST_ of 0.01 eV was recorded by strong CT properties, the bathochromic shift of the emission spectrum damages the color purity of the ***bf*DOB‐BN3** emitter. Furthermore, Huang–Rhys factor analysis of ***bf*DOB‐BN1**, ***bf*DOB‐BN2**, and ***bf*DOB‐BN3** reveals a clear difference in their total reorganization energies (Figure 3c,f,i). ***bf*DOB‐BN1** and ***bf*DOB‐BN2** show relatively small total λ values of 336.3 and 380.7 cm^−^^1^, respectively, whereas ***bf*DOB‐BN3** exhibits a markedly larger value of 698.7 cm^−^^1^, nearly twice that of the others. Especially, this difference is most pronounced in the high‐frequency vibrational region (1622 and 1625 cm^−^^1^), which may significantly contribute to emission bandwidth broadening (Figure S25). The substantially larger involvement of these vibrations in ***bf*DOB‐BN3** reflects a greater displacement between the S_0_ and S_1_ potential energy surfaces, leading to enhanced vibronic coupling and, consequently, a broader emission spectrum compared with ***bf*DOB‐BN1** and ***bf*DOB‐BN2**. Comparing the photophysical properties of the three emitters, ***bf*DOB‐BN2** is ideal as the deep blue MR‐TADF emitter because it shows a deep blue emission peak, a small FWHM, and a small ΔE_ST_ at the same time with the help of the meta‐oriented benzofuran.

**FIGURE 4:**
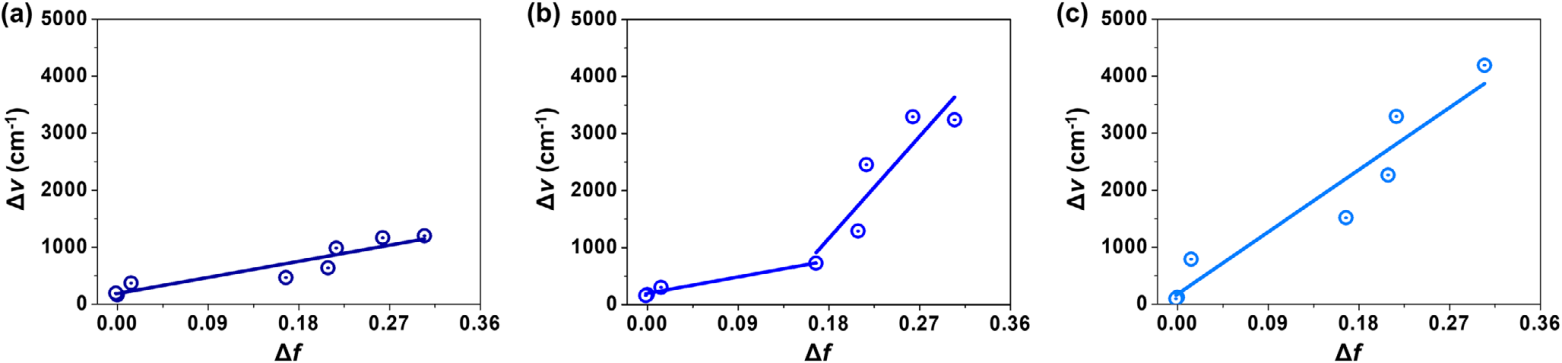
Lippert–Mataga plots of (a) ***bf*DOB‐BN1**, (b) ***bf*DOB‐BN2**, and (c) ***bf*DOB‐BN3**. The x‐axis represents the solvent orientation polarizability (Δ*f*), while the y‐axis corresponds to the Stokes shift (*Δν*, cm^−^^1^). Linear fits were applied to the data points for each emitter.

To further investigate the emission nature of the emitters, solvatochromism was analyzed by measuring their PL spectra in solvents with different polarities (Figures S26–S28, Table S2). ***bf*DOB‐BN1** showed relatively minor changes in the emission wavelength and FWHM compared to the other two emitters, indicating a weak LRCT character. In contrast, ***bf*DOB‐BN2** exhibited a pronounced solvatochromic shift from deep blue to green region, reaching a maximum wavelength of 514 nm with an FWHM of 96 nm in acetonitrile. ***bf*DOB‐BN3** showed an even more significant redshift of the emission spectrum in polar acetonitrile solvent. Based on these results, a Lippert–Mataga plot of the Stokes shift vs. the solvent polarity function (Δ*f*) was recorded [47, 48, 49]. Among the three emitters, only ***bf*DOB‐BN2** (Figure 4b) displayed a dual‐slope behavior in the Lippert–Mataga plot, suggesting a clear HCT character arising from the moderate mixing of SRCT by the MR effect and LRCT by meta‐oriented fused benzofuran. Specifically, ***bf*DOB‐BN2** exhibits a relatively small slope of 3189 cm^−^^1^ in the low‐polarity regime, followed by a markedly increased slope of 19 873 cm^−^^1^ in the high‐polarity regime, clearly reflecting the polarity‐dependent evolution of the excited‐state character. In contrast, both ***bf*DOB‐BN1** (Figure 4a) and ***bf*DOB‐BN3** (Figure 4c) exhibited a linear relationship with a single slope, suggesting that the emission is dominantly governed by a single emission mechanism, in agreement with the SRCT and LRCT calculation results. The small slope observed for ***bf*DOB‐BN1** (3138 cm^−^^1^) in the Lippert–Mataga plot indicates minimal sensitivity of the emission spectrum to solvent polarity, which is consistent with a typical SRCT‐dominant emission behavior. Conversely, the steep slope observed for ***bf*DOB‐BN3** (12 120 cm^−^^1^) reflects strong solvent‐dependent emission character, as commonly observed in LRCT‐dominant emission (Table S3). These observations are also consistent with the calculated changes in dipole moment between the ground and excited states (Table S4), obtained from DFT calculation for the S_0_ state and TDDFT calculation for the S_1_ state. The magnitude of the dipole moment changes increases in the order ***bf*DOB‐BN1**< ***bf*DOB‐BN2**< ***bf*DOB‐BN3**, indicating progressively stronger differences in charge redistribution upon excitation. These differences affect how the molecules interact with the solvent environment, leading to a progressively stronger solvatochromic response from ***bf*DOB‐BN1** to ***bf*DOB‐BN3**. Therefore, the emission mechanism of the MR‐TADF emitter represented by SRCT and LRCT could be controlled by managing the fusion position of the benzofuran within a PAH structure. The LRCT character was strengthened by stepwise addition of meta‐oriented benzofuran in the PAH structure.

The emission properties of the three emitters are well correlated with the HOMO and LUMO energy levels collected by cyclic voltammetry (CV) (Figure S29). The CV analysis showed that the three emitters possessed similar HOMO levels of about −5.59 eV, but different LUMO levels depending on the position of fused benzofuran. ***bf*DOB‐BN1** featured the shallowest LUMO level of −2.43 eV, while ***bf*DOB‐BN2** and ***bf*DOB‐BN3** recorded deepened LUMO levels of −2.65 and −2.70 eV, respectively. The addition of the para ‐oriented benzofuran induced shallowing of the LUMO because of the weakened electron deficiency of the boron atom through the para‐oriented oxygen atom. The HOMO‐LUMO gap becomes small by incorporating the meta‐oriented benzofuran, which agrees with the simulation result that ***bf*DOB‐BN3** exhibits the most extensive LUMO delocalization and the strongest LRCT character among the emitters.

To elucidate the influence of CT character on exciton dynamics, transient PL measurements were performed using 1 wt.% emitter doped 3‐CzPB (2,6‐bis(3‐(9H‐carbazol‐9‐yl)phenoxy)benzonitrile) films. Despite indications of multicomponent behavior in the decay profiles, all emission decays were analyzed using a bi‐exponential fitting model (expdec2), which provides physically meaningful parameters and more reliably captures the overall decay characteristics than higher‐order fitting. The fitting analysis yields the prompt lifetime (τ_p_) and delayed lifetime (τ_d_). Additionally, the prompt and delayed contributions were determined by integrating the emission intensity over the prompt region and the delayed region (from the end of the prompt component to 50 µs), respectively. (Figure 3b,e,h). The prompt decay components of the three emitters were similar, whereas the τ_d_ values of the ***bf*DOB‐BN1**, ***bf*DOB‐BN2**, and ***bf*DOB‐BN3** were 5.8, 2.3, and 1.9 µs, respectively, following the CT character of the three emitters. The increase of LRCT contribution to the emission process accelerated the delayed emission process. This systematic analysis highlights how the modulation of CT character governs the RISC rate and ultimately impacts exciton harvesting efficiency in MR‐TADF emitters. PL quantum yield (PLQY) of the three emitters was 99%, proposing efficient RISC process and suppressed non‐radiative decay pathways through the rigid benzofuran fused PAH structure. Remarkably, the *k_rISC_* increased dramatically from 5.0 × 10^5^ s^−^^1^ (***bf*DOB‐BN1**) to 2.1 × 10^6^ s^−^^1^ (***bf*DOB‐BN2**), and 2.3 × 10^6^ s^−^^1^ (***bf*DOB‐BN3**). This pronounced enhancement of RISC rate is consistent with the decreasing ΔE_ST_ values and is attributed to the incorporation of LRCT. The RISC rates recorded in the ***bf*DOB‐BN2** and ***bf*DOB‐BN3** are among the top‐tier values reported in the one‐and two‐boron‐based MR‐TADF emitters. The limitation of the slow RISC process in the one‐and two‐boron‐based MR‐TADF emitters could be overcome by inducing LRCT within the PAH framework through the fused benzofuran unit. All photophysical parameters of the emitters are summarized in Table 1. To further assess the RISC process within a Marcus‐type framework, temperature‐dependent transient PL measurements were performed and analyzed using a modified Arrhenius approach to extract effective kinetic parameters (Figures S30 and S31, Table S5) [50, 51]. Although quantitative differences exist between the experimentally obtained λ_eff_ and SOC_eff_ values and those derived from quantum‐chemical calculations, the experimental results follow a trend comparable to that predicted by the calculations across the three emitters. Overall, this comparison helps bridge the gap between theoretical predictions and experimental observations within a Marcus‐type framework for the RISC process.

**TABLE 1:** Photophysical properties of *bf*DOB‐BN1, *bf*DOB‐BN2, and *bf*DOB‐BN3.

Emitters	*λ*_abs_ ^a^ (nm)	*λ*_emi_ ^a^ (nm)	FWHM^a^ (nm)	*E*_HOMO_ ^b^ (eV)	*E_L_*_UMO_ ^c^ (eV)	S_1_ ^d^ (eV)	T_1_ ^d^ (eV)	Δ*E*_ST_ ^d^ (eV)	*τ*_p_ ^e^ (ns)	*τ*_d_ ^e^ (µs)	Φ^e^ (%)	*k_ISC_* ^f^ (10^8^s^−1^)	*k_rISC_* ^f^ (10^6^s^−1^)
*bf*DOB‐BN1	435	442	18	−5.58	−2.43	2.74	2.66	0.08	5.80	5.84	99	1.1	0.5
*bf*DOB‐BN2	441	447	20	−5.59	−2.65	2.76	2.72	0.04	5.49	2.28	99	1.4	2.1
*bf*DOB‐BN3	449	465	30	−5.59	−2.70	2.70	2.69	0.01	5.66	1.90	99	1.4	2.3

^a^ Absorption maximum (*λ*_abs_), fluorescence maximum (*λ*_emi_) and FWHM measured in toluene (10^−5^ m, 298K);

^b^ HOMO energy level measured by cyclic voltammetry in methylene chloride;

^c^ LUMO energy level estimated from HOMO and optical bandgap;

^d^ S₁ and T₁ energy levels estimated from fluorescence and phosphorescence maxima in toluene at 77 K, and ΔE_ST_ represents the energy gap between S_1_ and T_1_;

^e^ Prompt (*τ*_P_), delayed (*τ*_d_) fluorescence lifetimes, and PLQY were measured in the 1% doped film;

^f^ *k_ISC_* and *k_rISC_* are rate constants for intersystem crossing and reverse intersystem crossing respectively.

Thermogravimetric analysis (TGA) was conducted to assess the thermal stability of the materials. The decomposition temperature (T_d_) was defined as the temperature at which the sample retained 90% of its initial mass (corresponding to a 5% weight loss). All three emitters exhibited excellent thermal stability, with T_d_ values of 455°C for ***bf*DOB‐BN1**, 494°C for ***bf*DOB‐BN2**, and 517°C for ***bf*DOB‐BN3** (Figure S32).

### 2.3 Device Performance

**FIGURE 5:**
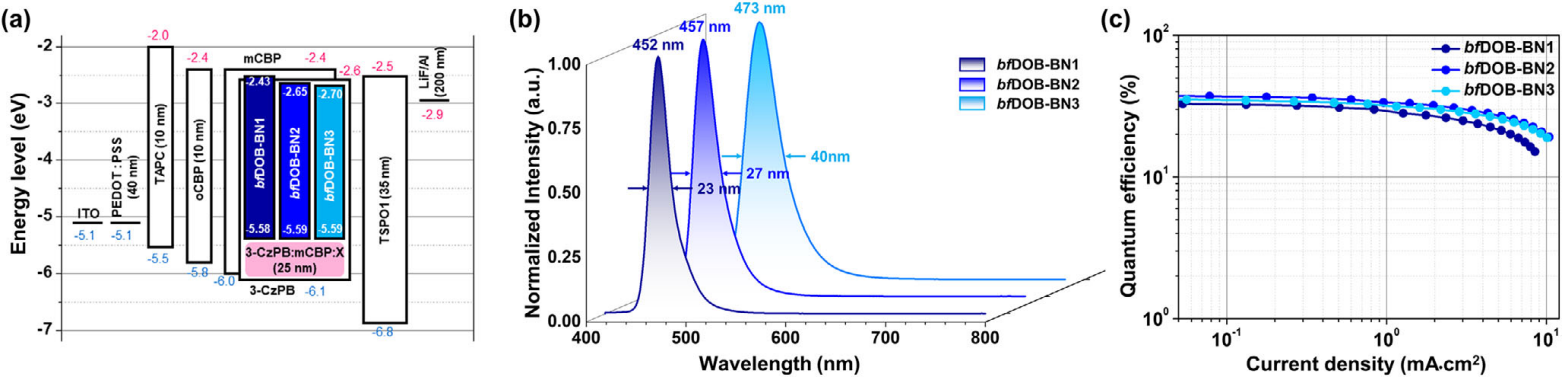
OLED device performance of ***bf*DOB‐BN1**, ***bf*DOB‐BN2**, and ***bf*DOB‐BN3** (1.0 wt.%) doped in 3‐CzPB: mCBP (7:3) host system. (a) The device structure and energy level diagram. (b) Normalized electroluminescence spectra. (c) Quantum efficiency‐current density curve.

To evaluate the electroluminescence performance of three blue emitters, a bottom‐emitting OLED device was fabricated through device optimization. Current density‐voltage‐luminance curves, electroluminescence spectra, quantum efficiency as a function of current density, and the device structure are presented in Figure 5 and Figure S33. Summarized device performances of emitters are presented in Table 2.

**TABLE 2:** OLED device performance of ***bf*DOB‐BN1**, ***bf*DOB‐BN2**, and ***bf*DOB‐BN3** (1.0 wt.%) doped in 3‐CzPB: mCBP (7:3) host system.

Emitter	λ_EL_ ^a^ (nm)	FWHM^a^ (nm)	CIE(x,y)^b^	QE^c^			CE^d^	Roll‐off^e^
				Max (%)	1 mA/cm^2^ (%)	5 mA/cm^2^ (%)	Max (%)	
*bf*DOB‐BN1	452	23	(0.145, 0.045)	32.8	29.3	21.7	16.0	33.8
*bf*DOB‐BN2	457	27	(0.139, 0.065)	37.5	33.5	27.2	26.1	27.5
*bf*DOB‐BN3	473	40	(0.120, 0.196)	37.1	31.9	26.0	50.4	29.9

^a^ Emission maximum, full width half maximum of electroluminescence;

^b^ CIE coordinates measured at 100 cd m^−2^;

^c^ QE (quantum efficiency) values measured at maximum, 1 mA/cm^2^ and 5 mA/cm^2^;

^d^ CE (current efficiency) values measured at maximum;

^e^ Roll‐off corresponds to QE (5 mA cm^−^^2^) / QE_max_.

The devices fabricated using a 3‐CzPB: mCBP (3,3’‐Di(9H‐carbazol‐9‐yl)‐1,1’‐biphenyl) (7:3) mixed host exhibited electroluminescence spectra redshifted by approximately 10 nm compared to the PL spectra in toluene accompanied by a slight broadening of the FWHM, providing peak wavelength/FWHM of 452/23, 457/27, and 473/40 nm for ***bf*DOB‐BN1**, ***bf*DOB‐BN2**, and ***bf*DOB‐BN3**, respectively. Both ***bf*DOB‐BN1** and ***bf*DOB‐BN2** demonstrated deep blue emission with a peak wavelength below 460 nm and an FWHM of less than 30 nm. However, the strong LRCT character of ***bf*DOB‐BN3** largely shifted the emission to the sky blue region. The color coordinates of the ***bf*DOB‐BN1**, ***bf*DOB‐BN2**, and ***bf*DOB‐BN3** devices were (0.145, 0.045), (0.139,0.065), and (0.120, 0.196) respectively. The balanced SRCT and LRCT characters of ***bf*DOB‐BN2** enabled deep blue emission close to BT 2020 standard without significant redshift and broadening of the emission spectrum.

All emitters achieved high EQEs exceeding 30%. Notably, ***bf*DOB‐BN2** and ***bf*DOB‐BN3** devices achieved exceptionally high EQEs of 37.5% and 37.1%, respectively. In particular, the EQE of the ***bf*DOB‐BN2** device is one of the highest EQEs of the one‐and two‐boron‐based MR‐TADF emitters with a y color coordinate of less than 0.10. The high EQE of the ***bf*DOB‐BN2** device is due to the high PLQY of 99% and the high horizontal emitting dipole orientation ratio of 86.7% as presented in Figure S34. The extension of the planar PAH structure through the benzofuran induced the extensive horizontal emitting dipole orientation of the ***bf*DOB‐BN2** emitter. Considering both these results and the optical simulation analysis (Table S6), the measured maximum EQE of 37.5% for **bfDOB‐BN2** translates into an effective internal quantum efficiency of approximately 98%, demonstrating an exceptionally high level of internal efficiency and outstanding exciton utilization enabled by intrinsically efficient exciton management through rapid RISC.

Additionally, the ***bf*DOB‐BN2** device performed better than the ***bf*DOB‐BN1** device in terms of EQE roll‐off because of enhanced *k_rISC_* through small ΔE_ST_ induced by hybridized excited states. The molecular design strategy aimed to establish an HCT state by appropriately balancing SRCT and LRCT characteristics within the PAH framework realized high EQE, small FWHM, and reduced EQE roll‐off in the deep blue OLEDs nearly satisfying the BT 2020 standard. In addition, the operational stability of the devices was further evaluated to assess the practical implications of the molecular design. Device lifetime measurements conducted under identical operating conditions reveal that the **bfDOB‐BN1** device exhibits the shortest operational stability, whereas **bfDOB‐BN2** and **bfDOB‐BN3** show comparable and clearly prolonged performance. This stability trend is consistent with the intrinsic properties of the emitters, where enhanced LRCT character facilitates faster RISC and suppresses the accumulation of long‐lived triplet excitons that are detrimental to device stability (Figure S35).

**FIGURE 6:**
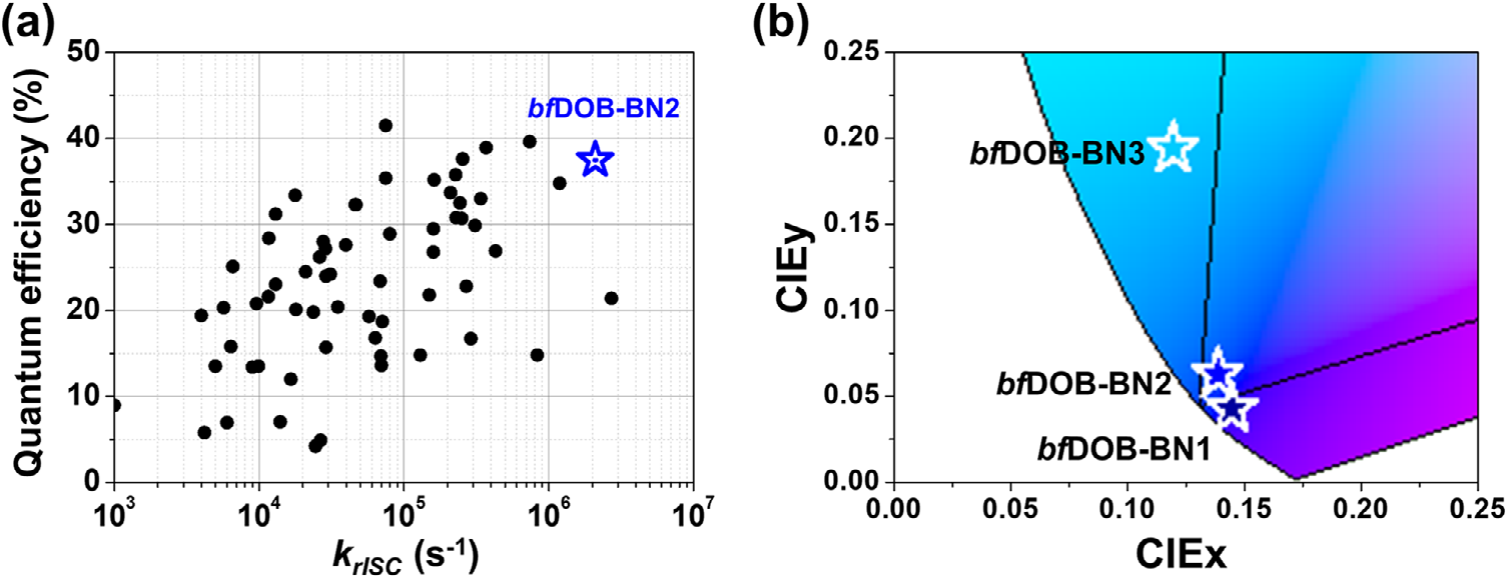
(a) Plot of quantum efficiency vs. *k_rISC_*, comparing ***bf*DOB‐BN2** with previously reported deep‐blue MR‐TADF emitters (CIEy≤0.1) one‐and two‐ boron‐based core. (b) CIE coordinates of ***bf*DOB‐BN1**, ***bf*DOB‐BN2**, and ***bf*DOB‐BN3**. The black line represents the BT.2020 standard.

Consequently, ***bf*DOB‐BN2** exhibits the fastest *k_rISC_* and an exceptionally high EQE among the deep‐blue MR‐TADF emitters based on one‐and two‐boron‐based cores reported to date (Figure 6; Table S7), a design space generally regarded as more compatible with practical requirements than the substantially π‐extended multi‐boron architectures, which, despite their excellent photophysical properties, often present challenges when transitioning toward practical implementation. Our approach achieves both narrowband emission and high efficiency in the deep‐blue region, providing an effective molecular design that advances the development of state‐of‐the‐art MR‐TADF emitters with strong potential for technologically relevant applications.

## 3 Conclusions

In this study, we demonstrated a new molecular design strategy to overcome the intrinsic limitations of slow RISC and deep blue emission color observed in one‐and two‐boron‐based MR‐TADF emitters by fusing benzofuran with DOBNA‐based main skeleton. The fusion of benzofuran allowed the LRCT excited state in the light emission process of the MR‐TADF emitters, enabling HCT excited states with both SRCT and LRCT characteristics. As a result, the ***bf*DOB‐BN2** emitter demonstrated narrow emission in the deep blue region through SRCT character and fast RISC process through LRCT character. The ***bf*DOB‐BN2** device delivered a remarkable EQE of 37.5% in the deep‐blue region (CIEy = 0.065) and a small FWHM of 27 nm along with improved EQE roll‐off. Overall, the LRCT modulation approach in the MR‐TADF emitters offers an effective strategy to engineer HCT states, enabling narrowband emission, fast RISC, and highly efficient deep‐blue electroluminescence, and provides a promising pathway for the development of state‐of‐the‐art deep‐blue OLEDs that combine high efficiency, color purity, and small EQE roll‐off.

## Conflicts of Interest

The authors declare no conflicts of interest.

## Supporting information




**Supporting File**: adma72861‐sup‐0001‐SuppMat.docx.

## Data Availability

The data that supports the findings of this study are available in the supplementary material of this article.
