# Supplementary material for: Hybridized Charge‐Transfer Window within a Fully Conjugated Multi‐Resonance Thermally Activated Delayed Fluorescence Framework for Ultrafast Reverse Intersystem Crossing and High‐Efficiency in Deep‐Blue Organic Light‐Emitting Diodes
Source: Adv Mater. 2026 Mar 24;38(23):e72861. doi: 10.1002/adma.72861 (PMC13103624; doi:10.1002/adma.72861)
Supplement: Supplementary file 1 — Supporting File: adma72861‐sup‐0001‐SuppMat.docx. [file ADMA-38-e72861-s001.docx]

Supporting information

Hybridized Charge-Transfer Window within a Fully Conjugated Multi-Resonance Thermally Activated Delayed Fluorescence Framework for Ultrafast Reverse Intersystem Crossing and High-Efficiency in Deep-Blue Organic Light-Emitting Diodes

Uisung Lee^1^, Kyungwoo Jeong^1^, Sunwoo Kang^2^***, Jun Yeob Lee^1,3,4^***

^1^Department of Display Engineering, Sungkyunkwan University
2066, Saburo, Jangan-gu, Suwon-si, Gyeonggi-do, 16419, Korea
^2^Department of Chemistry, Dankook University,
119, Dandaero, Dongnam-gu, Cheonan-si, Chuncheongnam-do, 31116, Republic of Korea
^3^School of Chemical Engineering, Sungkyunkwan University
2066, Seobu-ro, Jangan-gu, Suwon, Gyeonggi, 16419, Republic of Korea
^4^SKKU Institute of Energy Science and Technology, Sungkyunkwan University
2066, Seobu-ro, Jangan-gu, Suwon, Gyeonggi, 16419, Republic of Korea

* Corresponding author

E-mail: [sunwoo.kang@dankook.ac.kr](mailto:sunwoo.kang@dankook.ac.kr) (Prof. S. Kang), [leej17@skku.edu](mailto:leej17@skku.edu) (Prof. J.Y. Lee)

**Table of Contents**

**1. General information**

**2. Computational details**

**3. Device fabrication**

**4. Synthesis of compounds**

**5. Supplementary Figures**

**6. Supplementary Tables**

**7. Reference**

**1. General information**

All reagents were purchased from commercial suppliers (Sigma-Aldrich, Alfa Aesar, and TCI) and used without further purification. Palladium catalysts and ligands were obtained from P&H Tech, and solvents for reactions and purification were purchased from Samchun Pure Chemical, Duksan Sci., and Daejung Chemical & Metal.

^1^H and ^13^C nuclear magnetic resonance (NMR) spectra of compounds in CDCl₃ were recorded on an AVANCE Ⅲ (Bruker, 700 MHz) spectrometer. Molecular weights of intermediates were determined by LC–MS using an expressionL-CMS (Advion) with APCI mode, and final compounds were measured on a JMS-700 (JEOL) with high-resolution FAB mode.

UV–vis absorption and photoluminescence (PL) spectra were collected at 1.0 × 10⁻⁵ M using a UV–vis spectrophotometer V-730 (JASCO) and a fluorescence spectrometer LS-55 (PerkinElmer), respectively.

Cyclic voltammetry (CV) measurements were performed on an Iviumstat (Ivium Tech.) in dichloromethane at a scan rate of 100 mV s⁻¹. Platinum wires served as the working and counter electrodes, while Ag/AgCl was employed as the reference electrode. The ferrocenium/ferrocene redox couple was used as an internal standard, and 0.1 M tetrabutylammonium perchlorate (TBAClO₄) was applied as the supporting electrolyte.

PL quantum yield and transient PL decay data were obtained using a Quantaurus QY Absolute system (Hamamatsu, C11347-11) and a Quantaurus-Tau system (Hamamatsu, C11367-31), respectively.

The rate constants associated with the electronic transitions were determined based on the commonly applied Adachi’s method, expressed as follows:


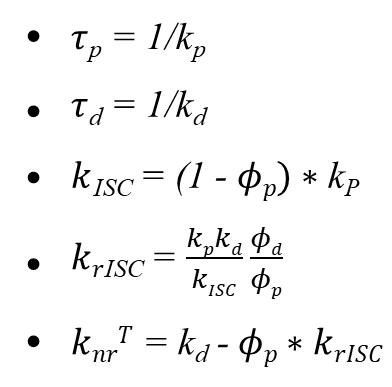


Φ_p_ ​ and Φ_d_ denote the prompt and delayed photoluminescence quantum yields, respectively; τ_p_ and τ_d_ correspond to decay lifetimes. The parameters *k*_p_​ and *k*_d_ ​represent the radiative rate constants of the prompt and delayed components, while *k*_ISC_ ​ and *k*_rISC_ describe the intersystem crossing and reverse intersystem crossing rates. In the present kinetic evaluation, nonradiative decay from the triplet manifold was assumed to be negligible (k_nr_^T^=0), consistent with the established three-state kinetic framework of TADF.^[1]^

Thermogravimetric analysis (TGA) was conducted using a TG/DTA7300 instrument (SEICO Instruments) under a nitrogen atmosphere, with the temperature ramped from room temperature to 600 °C. The decomposition temperature (T_d_​) was defined as the temperature at which a 5% loss of the initial sample mass occurred.

Lippert–Mataga analysis was performed based on solvent-dependent UV–vis absorption and photoluminescence (PL) measurements conducted in various solvents at a concentration of 10^−5^ M. The corresponding Stokes shifts were determined from the maxima of the absorption and emission spectra. The solvent-dependent PL behavior was analyzed using the Lippert–Mataga model, where the slope of the Lippert–Mataga plot—constructed based on the equation given below—was employed to qualitatively assess the charge-transfer character of the excited state. Linear fitting of the Stokes shift as a function of the solvent polarity function was carried out, and the resulting fitting parameters are summarized in **Table S4**.

$\boldsymbol{\Delta}\bar{\boldsymbol{v}}\boldsymbol{=}{\bar{\boldsymbol{v}}}_{\boldsymbol{abs}}\boldsymbol{-}{\bar{\boldsymbol{v}}}_{\boldsymbol{emi}}= \frac{2{{(\mu}_{e}-\mu_{g})}^{2}}{hca^{3}}\Delta f+const$ Eq. (1)

$\Delta f=(\frac{\varepsilon-1}{2\varepsilon+1})-(\frac{n^{2}-1}{2n^{2}-1})$ Eq. (2)

The modified Arrhenius plot was constructed based on temperature-dependent transient PL measurements (1.0 wt% doped in 3-CzPB film) carried out at 300, 270, 240, and 210 K. The delayed fluorescence lifetime (τ_D_) was extracted at each temperature, and the effective RISC rate constant was estimated from the inverse of the delayed fluorescence lifetime. Using the modified Arrhenius equation described below, effective kinetic parameters were extracted from linear fitting of ln(*k_rISC,eff_T^1/2^*) as a function of inverse temperature.

$\ln(k_{rISC,eff}\cdot T^{1/2}$) = ln($\frac{2\pi{|HSOC|}^{2}}{\hbar\sqrt{4\pi\lambda kB}}) -\frac{{(\Delta EST+\lambda)}^{2}}{4\lambda kB}\cdot\frac{1}{T}$ EEQeqe

Eq. (3)

Angle-dependent photoluminescence measurements for evaluating the emitting dipole orientation were carried out using a setup composed of a rotational stage and a fused-silica half-cylindrical lens. Thin-film samples were fabricated on quartz substrates and encapsulated to prevent exposure to ambient conditions. Each film was mounted onto the flat surface of the half-cylinder lens using refractive index-matching oil. A 325 nm He–Cd laser served as the excitation source, with the beam intensity controlled by neutral-density filters. p-Polarized PL emission, selected using a linear polarizer, was collected by a spectrometer (MAYA 2000 Pro, Ocean Optics Inc.) while the sample stage was continuously rotated.

**2. Computational details**

According to the previous reports,^[2]^ ω parameter should be tuned to quantitatively describe the nature of CT excitation. Moreover, the optimal ω (ω*) should be individually determined, depending on the molecular system. The following equation expressed that the ω^*^ can be determined at the minimum point of *J^2^(ω)* as a function of ω.

$J^{2}\left( \omega\right)= \sum_{i=0}^{1} {[\varepsilon_{H}\left( N+i \right)+IP\left( N+i \right)]}^{2}$ Eq. (4)

, where $\varepsilon_{H}$, *N*, IP indicate the energy level of the HOMO, electron number of the system, and ionization potential, respectively. All molecular structures of ground state (S_0_), S_1_, and T_1_ states were optimized without any symmetry constraints (C1) in the gas phase. The frequency calculations were performed to confirm their thermodynamic stability at the individual optimized structures. Unfortunately, although the optimally tuned range separated DFT functionals beneficially describe the long-range CT excitation, the core structure in these synthesized compounds has been known to exhibit the short-range CT excitation, nominated as MR type. According to the previous reports, it is demonstrated that the use of DFT methodology to predict both excited state energies of MR type emitter is useless to describe the exact nature of both excited states. To solve this critical issue, it is previously suggested that the methodology considering the electron-electron correlation effect guarantees a quantitative prediction of both excited state energies. Therefore, several methodologies such as ADC(2), STEOM-DLPNO-CCSD, EOM-CCSD, and double hybrid (DH) DFT functionals are proposed to investigate the understanding of the nature of excited states in an MR type emitter.^[3]^ In present work, STEOM-DLPNO-CCSD calculations were further conducted to obtain the quantitative excited-state energies at the optimized structures from DFT calculations. Due to the limit of molecular size and computing resources, SVP basis set was used to compute the STEOM-DLPNO-CCSD calculations.

The scalar relativistic zero order regular approximation (ZORA) Hamiltonian was utilized to compute the spin-orbit coupling (SOC) constant between S_1_ and T_1_ states, as implemented in suite of ORCA 5.0 program.^[4]^ The spin-orbit coupling constants of <S_0_|H_SOC_|T_1_> and <S_1_|H_SOC_|T_1_> were calculated at the optimized T_1_ structures. The intersystem and reverse intersystem crossing rate (*k_ISC_* and *k_rISC_*) can be obtained by semi-empirical Marcus theory formalism as the following equation.^[5]^

$k_{rISC}=\frac{4\pi^{2}{\langle S_{1}\left| H_{SOC} \right|T_{1}\rangle}^{2}}{h}\frac{1}{\sqrt{4\pi k_{b}T\lambda}}exp(-\left( \frac{\left( {\Delta E}_{ST}+\lambda\right)^{2}}{4\lambda k_{b}T} \right))$ Eq. (5)

, where <S_1_|H_SOC_|T_1_>, *λ*, *k_b_*, *h*, and T are defined as spin-orbit coupling constant between S_1_ and T_1_ states, reorganization energy (S_1_ and T_1_ state), Boltzmann constant, Plank constant, and temperature, respectively.

The Huang-Rhys factor can be calculated by following equation.

$Huang-Rhys factor= \frac{1}{2}\omega_{i}K_{i}^{2}$ Eq. (6)

, where $K_{i}$ is the shift vector and *ω*_i_ is the vibrational frequency of *i*-th normal vibrational mode. The $K_{i}$ can be defined as dimensionless displacement vector corresponding to changes in geometries between initial and final state of *i*-th normal vibrational mode.^[6]^

**3. Device fabrication**

Devices were fabricated by vacuum thermal evaporation under high-vacuum conditions. Following deposition, the devices were encapsulated under ambient atmosphere using a standard UV-curable epoxy procedure. Electrical characteristics were recorded with a Keithley 2400 source meter, and optical properties were measured using a Konica Minolta CS-2000 spectroradiometer. The device structure of emitters is as follows: indium tin oxide (ITO, 50 nm)/PEDOT:PSS (40 nm)/TAPC (10 nm)/oCBP (10 nm)/ 3-CzPB:mCBP (25 nm: 30 wt%: 1%)/TSPO1 (35 nm)/LiF (1.5 nm)/Al (200 nm). The full name of the materials is as follows: PEDOT:PSS is poly(3,4-ethylenedioxythiophene) polystyrene sulfonate, TAPC is 1,1-bis[(di-4-tolylamino)phenyl]cyclohexane, oCBP is 2,2`-di(9H-carbazol-9-yl)-1,1`-biphenyl, 3-CzPB is 2,6-bis(3-(9*H*-carbazol-9-yl)phenoxy)benzonitrile, mCBP is 3,3`-di(9H-carbazol-9-yl)-1,1`-biphenyl, and TSPO1 is diphenyl[4-(triphenylsilyl)phenyl]phosphine oxide. **Figure S6** shows the molecular structures of the materials used to fabricate the device.

**4. Synthesis of compounds.**

**Scheme S1. Synthesis scheme of *bf*DOB-BN1, *bf*DOB-BN2 and *bf*DOB-BN3.**

***Synthesis of N-(3-bromo-5-methylphenyl)-2,4,6-trimethyl-N-phenylaniline (A-2)***

3,5-dibromotoluene (10.0 g, 40.0 mmol), 2,4,6-trimethylaniline (5.7 g, 42.0 mmol), Pd_2_(dba)₃ (0.4 g, 0,4 mmol), BINAP (0.5 g, 0.8 mmol), and sodium tert-butoxide (5.8 g, 60 mmol) were added to a 2-neck round-bottom flask and dissolved in toluene. The reaction mixture was purged with N₂three times and then heated at 100°C for 3 h. After reaction, the mixture was cooled to room temperature and extracted with toluene and deionized water (DW). The combined organic layers were concentrated under reduced pressure using a rotary evaporator, and the residue was purified by column chromatography to afford **A-2** as a white solid. (9.0 g, yield: 74%)***.* ^1^H NMR** (700 MHz, CDCl_3_) δ 6.95 (s, 2H), 6.69 (s, 1H), 6.41 (s, *1*H), 6.24 (s, 1H), 2.32 (s, 3H), 2.21 (s, 3H), 2.17 (s, 6H). MS (APCI) m/z 305.2 [(M+H)^+^].

***Synthesis of 2-bromo-1-(4-(tert-butyl)phenoxy)-3-(3-chlorophenoxy)benzene (A-1)***

**A-2** (8.0 g, 26.3 mmol), iodobenzene (5.9 g, 28.9 mmol), Pd_2_(dba)₃ (0.2 g, 0.3 mmol), tBu_3_PHBF_4_ (0.2 g, 0.5 mmol), and sodium tert-butoxide (3.8 g, 39.4 mmol) were added to a 2-neck round-bottom flask and dissolved in toluene. The reaction mixture was purged with nitrogen three times and then heated at 90°C for 2 h. After reaction, the mixture was cooled to room temperature and extracted with toluene and deionized water (DW). The combined organic layers were concentrated under reduced pressure using a rotary evaporator, and the residue was purified by column chromatography to afford **A-1** as a beige solid. (8.8 g, yield: 89%). **^1^H NMR** (700 MHz, CDCl_3_) δ 7.23 – 7.17 (m, 2H), 6.99 – 6.95 (m, 2H), 6.93 (s, 2H), 6.91 (tt, *J* = 7.4, 1.1 Hz, 1H), 6.88 (t, *J* = 1.7 Hz, 1H), 6.82 (s, 1H), 6.67 (s, 1H), 2.33 (s, 3H), 2.19 (s, 3H), 1.99 (s, 6H). MS (APCI) m/z 381.3 [(M+H)^+^].

***Synthesis of N1,N3-dimesityl-5-methyl-N1-phenylbenzene-1,3-diamine (A)***

**A-1** (8.0 g, 21.0 mmol), 2,4,6-trimethylaniline (3.1 g, 23.1 mmol), Pd_2_(dba)₃ (0.4 g, 0.4 mmol), tBu_3_PHBF_4_ (0.2 g, 0.8 mmol), and sodium tert-butoxide (3.0 g, 31.6 mmol) were added to a 2-neck round-bottom flask and dissolved in toluene. The reaction mixture was purged with nitrogen three times and then heated at 90°C for 3 h. After reaction, the mixture was cooled to room temperature and extracted with toluene and deionized water (DW). The combined organic layers were concentrated under reduced pressure using a rotary evaporator, and the residue was purified by column chromatography to afford **A** as a white solid. (8.3 g, yield: 91%). **^1^H NMR** (700 MHz, CDCl_3_) δ 7.28 (s, 1H), 7.18 – 7.14 (m, 2H), 6.97 (dd, *J* = 8.6, 0.9 Hz, 2H), 6.91 (s, 2H), 6.88 (s, 2H), 6.84 (t, *J* = 7.2 Hz, 1H), 6.34 – 6.12 (m, 1H), 6.10 – 6.01 (m, 1H), 2.34 (s, 3H), 2.29 (s, 3H), 2.18 (s, 6H), 2.11 (s, 3H), 1.99 (s, 6H). MS (APCI) m/z 435.0 [(M+H)^+^].

***Synthesis of N1-(3-(dibenzo[b,d]furan-1-yloxy)-5-(dibenzo[b,d]furan-2-yloxy)phenyl)-N1,N3-dimesityl-5-methyl-N3-phenylbenzene-1,3-diamine (AB1)***

**A** (2.5 g, 5.8 mmol), **B1** (3.3 g, 6.3 mmol), Pd_2_(dba)₃ (0.3 g, 0.3 mmol), tBu_3_PHBF_4_ (0.2 g, 0.6 mmol), and sodium tert-butoxide (1.1 g, 11.5 mmol) were added to a 2-neck round-bottom flask and dissolved in toluene. The reaction mixture was purged with nitrogen three times and refluxed for 12 h. After reaction, the mixture was cooled to room temperature and extracted with toluene and deionized water (DW). The combined organic layers were concentrated under reduced pressure using a rotary evaporator, and the residue was purified by column chromatography to afford **AB1** as a white solid. (4.1 g, yield: 82%). **^1^H NMR** (700 MHz, CDCl_3_) δ 7.85 (dd, *J* = 7.7, 0.6 Hz, 1H), 7.54 (d, *J* = 8.2 Hz, 1H), 7.43 (ddd, *J* = 8.4, 7.3, 1.3 Hz, 1H), 7.31 (t, *J* = 8.0 Hz, 1H), 7.27 (dd, *J* = 8.2, 0.8 Hz, 1H), 7.24 (dd, *J* = 7.5, 0.7 Hz, 1H), 7.21 – 7.18 (m, 2H), 7.11 – 7.09 (m, 2H), 6.99 (tt, *J* = 7.6, 1.0 Hz, 1H), 6.89 (d, *J* = 1.0 Hz, 1H), 6.88 (dt, *J* = 3.1, 1.4 Hz, 2H), 6.87 – 6.86 (m, 1H), 6.86 (s, 2H), 6.83 (s, 1H), 6.82 (s, 2H), 6.81 (dd, *J* = 7.9, 0.7 Hz, 1H), 6.75 (t, *J* = 6.8 Hz, 1H), 6.44 (dt, *J* = 16.9, 2.1 Hz, 2H), 6.17 (d, *J* = 25.6 Hz, 2H), 6.05 (t, *J* = 2.1 Hz, 1H), 2.29 (s, 3H), 2.27 (s, 3H), 2.04 (s, 3H), 2.00 (s, 6H), 1.92 (s, 6H). MS (APCI) m/z 785.5 [(M)^+^].

***Synthesis of N1-(3-(dibenzo[b,d]furan-2-yloxy)-5-phenoxyphenyl)-N1,N3-dimesityl-5-methyl-N3-phenylbenzene-1,3-diamine (AB2)***

**AB2** was prepared following the procedure described for synthesis of **AB1**, replacing **B1** with **B2** (2.7 g, 6.3 mmol) to afford **AB2** as a white solid. (3.7 g, yield: 81%). **^1^H NMR** (700 MHz, CDCl_3_) δ 7.85 (dd, *J* = 44.6, 7.7 Hz, 2H), 7.53 (dd, *J* = 7.7, 6.9 Hz, 2H), 7.48 (d, *J* = 2.4 Hz, 1H), 7.47 – 7.43 (m, 1H), 7.43 – 7.40 (m, 1H), 7.39 (d, *J* = 8.8 Hz, 1H), 7.32 (t, *J* = 7.2 Hz, 1H), 7.29 (t, *J* = 8.1 Hz, 1H), 7.24 (t, *J* = 7.2 Hz, 2H), 7.11 (t, *J* = 7.9 Hz, 2H), 7.03 (dd, *J* = 8.8, 2.5 Hz, 1H), 6.90 (d, *J* = 7.5 Hz, 3H), 6.86 (s, 2H), 6.80 (d, *J* = 6.5 Hz, 3H), 6.74 (t, *J* = 7.6 Hz, 1H), 6.45 (d, *J* = 17.8 Hz, 2H), 6.16 (d, *J* = 31.1 Hz, 2H), 6.05 (t, *J* = 1.7 Hz, 1H), 2.29 (s, 3H), 2.23 (s, 3H), 2.03 (s, 3H), 2.02 (s, 6H), 1.92 (s, 6H).MS (APCI) m/z 876.6 [(M+H)^+^].

***Synthesis of N1-(3,5-bis(dibenzo[b,d]furan-2-yloxy)phenyl)-N1,N3-dimesityl-5-methyl-N3-phenylbenzene-1,3-diamine (AB3)***

**AB3** was prepared following the procedure described for synthesis of **AB1**, replacing **B1** with **B3** (3.3 g, 6.3 mmol) to afford **AB3** as a white solid. (4.1 g, yield: 83%). **^1^H NMR** (700 MHz, CDCl_3_) δ 7.88 (d, *J* = 7.7 Hz, 2H), 7.58 (dd, *J* = 17.3, 5.3 Hz, 4H), 7.51 – 7.46 (m, 4H), 7.35 (t, *J* = 7.5 Hz, 2H), 7.23 (t, *J* = 7.9 Hz, 2H), 7.19 (dd, *J* = 8.8, 2.5 Hz, 2H), 7.03 (s, 1H), 7.02 (d, *J* = 2.5 Hz, 2H), 6.95 (s, 2H), 6.90 (s, 2H), 6.85 (t, *J* = 7.2 Hz, 1H), 6.54 (d, *J* = 2.0 Hz, 2H), 6.33 (s, 1H), 6.26 (s, 1H), 6.13 (t, *J* = 2.1 Hz, 1H), 2.36 (s, 3H), 2.25 (s, 3H), 2.15 (s, 6H), 2.11 (s, 3H), 2.04 (s, 6H). MS (APCI) m/z 876.5 [(M+H)^+^].

***Synthesis of 14,18-dimesityl-16-methyl-14,18-dihydro-7,12,23-trioxa-14,18-diaza-4b,22b-diboraindeno[1,2-a]dinaphtho[1,2,3-fg:1',2',3'-jk]pentacene (bfDOB-BN1)***

**AB1** (3.5 g, 4.5 mmol) and boron triiodide (6.1 g, 15.6 mmol) were added to the one-neck round-bottom flask under nitrogen and dissolved in anhydrous 1,2-dichlorobenzene. The mixture was heated 160℃ and stirred for 2 days. After reaction, the mixture was cooled to room temperature and excess DIEPA was added to quench the reaction. The crude mixture was extracted with DCM/DW and the combined organic layers were concentrated under reduced pressure. The residue was purified by column chromatography to afford ***bf*DOB-BN1** as a yellow solid. (0.6 g, yield: 17%). **^1^H NMR** (700 MHz, CDCl_3_) δ 8.96 (dd, *J* = 7.7, 1.5 Hz, 1H), 8.80 (d, *J* = 8.6 Hz, 1H), 8.78 (dd, *J* = 7.7, 1.5 Hz, 1H), 8.33 (dd, *J* = 7.5, 0.7 Hz, 1H), 7.70 (ddd, *J* = 8.4, 6.9, 1.6 Hz, 1H), 7.65 (d, *J* = 8.1 Hz, 1H), 7.60 (d, *J* = 8.2 Hz, 2H), 7.50 (dddd, *J* = 15.7, 8.5, 7.0, 1.5 Hz, 2H), 7.45 (td, *J* = 8.3, 1.0 Hz, 2H), 7.27 (s, 2H), 7.23 (ddd, *J* = 7.7, 6.9, 1.0 Hz, 1H), 7.19 (s, 2H), 6.77 (d, *J* = 8.5 Hz, 1H), 6.62 (s, 1H), 6.10 (s, 1H), 6.00 (s, 1H), 2.57 (s, 3H), 2.49 (s, 3H), 2.23 (s, 3H), 2.00 (s, 6H), 1.94 (s, 6H). **^13^C NMR** (176 MHz, CDCl_3_) δ 162.93, 160.38, 159.71, 159.69, 156.70, 155.63, 151.94, 145.89, 144.86, 144.76, 143.40, 138.43, 138.27, 138.13, 137.49, 137.09, 136.96, 136.85, 134.13, 133.55, 132.84, 131.42, 130.58, 130.24, 126.70, 123.28, 123.19, 123.12, 122.87, 119.07, 118.62, 114.83, 113.15, 111.39, 107.29, 105.93, 105.17, 94.86, 77.20, 77.02, 76.84, 23.22, 21.42, 21.30, 17.74, 17.72. **HRMS** (FAB+) m/z 800.3388 [(M+H)+]; Calcd. For C_55_H_42_B_2_N_2_O_3_, 800.3382.

***Synthesis of 4,26-dimesityl-2-methyl-4,26-dihydro-6,11,15,21-tetraoxa-4,26-diaza-13b,21c-diboraindeno[1,2-a]indeno[1',2':6,7]naphtho[1,2,3-fg]naphtho[1,2,3-jk]pentacene (bfDOB-BN2)***

***bfDOB-BN2*** was prepared following the procedure described for synthesis of ***bf*DOB-BN1**, replacing **AB1** with **AB2** (3.0 g, 3.4 mmol) to afford ***bf*DOB-BN2** as a yellow solid. (0.6 g, yield: 18%). **^1^H NMR** (700 MHz, CD_2_Cl_2_) δ 9.06 (dd, *J* = 7.6, 1.6 Hz, 1H), 8.94 (s, 1H), 8.93 – 8.90 (m, 1H), 8.33 (ddd, *J* = 7.5, 1.4, 0.7 Hz, 1H), 8.19 (s, 1H), 8.08 (ddd, *J* = 7.6, 1.4, 0.7 Hz, 1H), 7.69 (d, *J* = 6.9 Hz, 1H), 7.68 – 7.66 (m, 2H), 7.61 – 7.59 (m, 1H), 7.57 (ddd, *J* = 8.5, 6.8, 1.7 Hz, 1H), 7.54 (ddd, *J* = 8.3, 7.1, 1.3 Hz, 1H), 7.47 (td, *J* = 7.3, 1.0 Hz, 1H), 7.42 (ddd, *J* = 7.9, 7.1, 0.9 Hz, 1H), 7.32 – 7.28 (m, 3H), 7.25 – 7.21 (m, 2H), 6.83 (dd, *J* = 8.7, 1.0 Hz, 1H), 6.69 (s, 1H), 6.15 (t, *J* = 0.9 Hz, 1H), 6.06 (t, *J* = 1.0 Hz, 1H), 2.58 (s, 3H), 2.50 (s, 3H), 2.23 (d, *J* = 0.7 Hz, 3H), 1.99 (s, 6H), 1.95 (s, 6H). **^13^C NMR** (176 MHz, CDCl_3_) δ 163.25, 159.71, 157.90, 156.70, 156.33, 155.64, 152.29, 151.99, 146.01, 144.89, 144.77, 143.43, 138.51, 138.44, 138.17, 137.51, 137.07, 136.97, 136.87, 133.12, 131.55, 130.59, 130.27, 129.06, 128.74, 126.72, 124.12, 123.28, 123.20, 123.10, 122.71, 121.79, 118.57, 114.90, 114.64, 113.22, 111.95, 111.41, 110.06, 107.42, 105.96, 105.24, 94.76, 77.43, 77.21, 77.03, 76.85, 23.24, 21.43, 21.33, 17.80, 17.76. **HRMS** (FAB+) m/z 890.3494 [(M⁺•)]. Calculated for C_61_H_44_B_2_N_2_O_4_: 890.3487.

***Synthesis of 11,15-dimesityl-13-methyl-11,15-dihydro-6,17,23,26-tetraoxa-11,15-diaza-6c,24b-diboraindeno[1,2-b]indeno[1',2':6,7]naphtho[1,2,3-fg]naphtho[1,2,3-jk]pentacene (bfDOB-BN3)***

***bf*DOB-BN3** was prepared following the procedure described for synthesis of ***bf*DOB-BN1**, replacing **AB1** with **AB3** (2.8 g, 3.2 mmol) to afford **AB3** as a yellow solid. (0.5 g, yield: 18%). **^1^H NMR** (700 MHz, CD_2_Cl_2_) δ 9.06 (dd, *J* = 7.7, 1.7 Hz, 1H), 8.99 (s, 1H), 8.96 (s, 1H), 8.22 (s, 1H), 8.12 – 8.08 (m, 2H), 8.03 (s, 1H), 7.69 (tt, *J* = 8.2, 0.8 Hz, 2H), 7.60 (dddd, *J* = 8.3, 7.3, 6.2, 1.3 Hz, 2H), 7.57 (ddd, *J* = 8.5, 6.8, 1.7 Hz, 1H), 7.43 (td, *J* = 7.4, 0.9 Hz, 2H), 7.30 (ddd, *J* = 7.7, 6.8, 1.1 Hz, 1H), 7.28 – 7.26 (m, 2H), 7.25 – 7.22 (m, 2H), 6.82 (dd, *J* = 8.8, 1.0 Hz, 1H), 6.49 (s, 1H), 6.14 (t, *J* = 1.0 Hz, 1H), 6.06 (t, *J* = 0.9 Hz, 1H), 2.54 (s, 3H), 2.50 (s, 3H), 2.22 (d, *J* = 0.9 Hz, 3H), 1.97 (s, 6H), 1.94 (s, 6H). **^13^C NMR** (176 MHz, CDCl_3_) δ 163.47, 160.25, 157.96, 157.90, 156.38, 152.36, 152.24, 152.20, 146.01, 144.89, 144.69, 143.37, 138.45, 138.16, 137.51, 137.09, 136.87, 131.55, 130.52, 130.26, 129.21, 128.95, 128.81, 128.74, 124.11, 124.02, 122.77, 121.80, 121.54, 118.55, 114.91, 114.19, 112.07, 112.02, 110.11, 108.92, 106.00, 105.23, 94.45, 77.21, 77.03, 76.84, 23.21, 21.39, 21.32, 17.79, 17.69. **HRMS** (FAB+) m/z 890.3482 [(M+H)+]; Calcd. For C_61_H_44_B_2_N_2_O_4_, 890.3487.

**Scheme S2. Synthesis scheme for intermediates B1, B2 and B3.**

***Synthesis of 1-bromo-3-fluoro-5-phenoxybenzene (B1-1)***

1-bromo-3,5-difluorobenzene (5.0 g, 25.9 mmol), phenol (2.7 g, 28.5 mmol) and potassium carbonate (5.4 g, 38.9 mmol) were added to a 2-neck round-bottom flask and dissolved in DMF. The reaction mixture was purged with N₂ three times and then heated at 140°C for 6 h. After reaction, the mixture was cooled to room temperature and extracted with dichloromethane and deionized water (DW). The combined organic layers were concentrated under reduced pressure using a rotary evaporator, and the residue was purified by column chromatography to afford **B1-1** as a colorless oil. (4.9 g, yield: 71%). **^1^H NMR** (700 MHz, CDCl_3_) δ 7.40 (t, *J* = 8.0 Hz, 2H), 7.21 (t, *J* = 7.4 Hz, 1H), 7.06 (d, *J* = 7.8 Hz, 2H), 6.97 (dt, *J* = 7.9, 1.9 Hz, 1H), 6.92 (s, 1H), 6.64 (dt, *J* = 10.0, 2.2 Hz, 1H). MS (APCI) m/z 267.8 [(M)^+^].

***Synthesis of 1-(3-bromo-5-phenoxyphenoxy)dibenzo[b,d]furan (B1)***

**B1-1** (4.0 g, 20.7 mmol), dibenzofuran-1-ol (4.2 g, 22.8 mmol) and cesium carbonate (10.1 g, 31.0 mmol) were added to a 2-neck round-bottom flask and dissolved in NMP. The reaction mixture was purged with N₂ three times and then heated at 180°C for 6 h. After reaction, the mixture was cooled to room temperature and extracted with dichloromethane and deionized water (DW). The combined organic layers were concentrated under reduced pressure using a rotary evaporator, and the residue was purified by column chromatography to afford **B1** as a white solid. (6.2 g, yield: 84%). **^1^H NMR** (700 MHz, CDCl_3_) δ 7.88 (ddd, *J* = 7.7, 1.2, 0.6 Hz, 1H), 7.59 (d, *J* = 8.2 Hz, 1H), 7.47 (ddd, *J* = 8.3, 7.3, 1.3 Hz, 1H), 7.44 – 7.40 (m, 2H), 7.37 – 7.33 (m, 2H), 7.31 (td, *J* = 7.7, 0.9 Hz, 1H), 7.16 (tt, *J* = 7.7, 1.0 Hz, 1H), 7.04 – 7.01 (m, 2H), 6.99 – 6.97 (m, 1H), 6.94 (dd, *J* = 6.8, 1.9 Hz, 1H), 6.90 – 6.87 (m, 1H), 6.71 (t, *J* = 2.2 Hz, 1H). MS (APCI) m/z 431.3 [(M)^+^].

***Synthesis of 1-(3-bromo-5-fluorophenoxy)dibenzo[b,d]furan (B2-1)***

**B2-1** was prepared following the procedure described for synthesis of **B1-1**, replacing phenol with dibenzofuran-1-ol (3.1 g, 17.1 mmol) to afford **B2-1** as a colorless oil. (4.1 g, yield: 74%). **^1^H NMR** (700 MHz, CDCl_3_) δ 7.91 (ddd, *J* = 7.6, 1.2, 0.6 Hz, 1H), 7.63 (d, *J* = 2.5 Hz, 1H), 7.61 – 7.56 (m, 2H), 7.50 (ddd, *J* = 8.4, 7.3, 1.3 Hz, 1H), 7.36 (td, *J* = 7.7, 0.9 Hz, 1H), 7.17 (dd, *J* = 8.8, 2.5 Hz, 1H), 6.96 (ddd, *J* = 7.9, 2.2, 1.7 Hz, 1H), 6.92 (t, *J* = 2.4 Hz, 1H), 6.65 (dt, *J* = 10.0, 2.2 Hz, 1H). MS (APCI) m/z 358.4 [(M+H)^+^].

***Synthesis of 1-(3-bromo-5-(dibenzo[b,d]furan-2-yloxy)phenoxy)dibenzo[b,d]furan (B2)***

**B2** was prepared following the procedure described for synthesis of **B1**, replacing dibenzofuran-1-ol with dibenzofuran-2-ol (3.1 g, 17.1 mmol) to afford **B2** as a white solid. (4.7 g, yield: 81%). **^1^H NMR** (700 MHz, CDCl_3_) δ 7.89 (d, *J* = 7.6 Hz, 2H), 7.61 (d, *J* = 2.4 Hz, 1H), 7.58 (dd, *J* = 8.2, 3.3 Hz, 2H), 7.54 (d, *J* = 8.8 Hz, 1H), 7.50 – 7.45 (m, 2H), 7.43 – 7.38 (m, 2H), 7.36 – 7.34 (m, 1H), 7.33 – 7.30 (m, 1H), 7.15 (dd, *J* = 8.8, 2.5 Hz, 1H), 6.96 – 6.93 (m, 2H), 6.87 – 6.84 (m, 1H), 6.73 (t, *J* = 2.2 Hz, 1H). MS (APCI) m/z 521.2 [(M)^+^].

***Synthesis of 2,2'-((5-bromo-1,3-phenylene)bis(oxy))didibenzo[b,d]furan (B3)***

1-bromo-3,5-difluorobenzene (2.0 g, 10.4 mmol), dibenzofuran-2-ol (4.8 g, 25.9 mmol) and cesium carbonate (10.1 g, 31.1 mmol) were added to a 2-neck round-bottom flask and dissolved in NMP. The reaction mixture was purged with N₂ three times and then heated at 180°C for 4 h. After reaction, the mixture was cooled to room temperature and extracted with dichloromethane and deionized water (DW). The combined organic layers were concentrated under reduced pressure using a rotary evaporator, and the residue was purified by column chromatography to afford **B3** as a white solid. (4.6 g, yield: 86%) ^1^H NMR (700 MHz, CDCl_3_) δ 7.91 – 7.88 (m, 2H), 7.63 (d, *J* = 2.5 Hz, 2H), 7.58 (d, *J* = 8.2 Hz, 2H), 7.56 (d, *J* = 8.7 Hz, 2H), 7.48 (ddd, *J* = 8.4, 7.3, 1.3 Hz, 2H), 7.36 – 7.33 (m, 2H), 7.18 (dd, *J* = 8.8, 2.5 Hz, 2H), 6.79 (d, *J* = 2.2 Hz, 2H), 6.65 (t, *J* = 2.2 Hz, 1H). MS (APCI) m/z 522.2 [(M+H)^+^].

**5. Supplementary Figures**

**

**Figure S1**. The plots of *J^2^*(ω) as a function of ω and their corresponding raw data in ***bf*DOB-BN1**, ***bf*DOB-BN2**, and ***bf*DOB-BN3**.


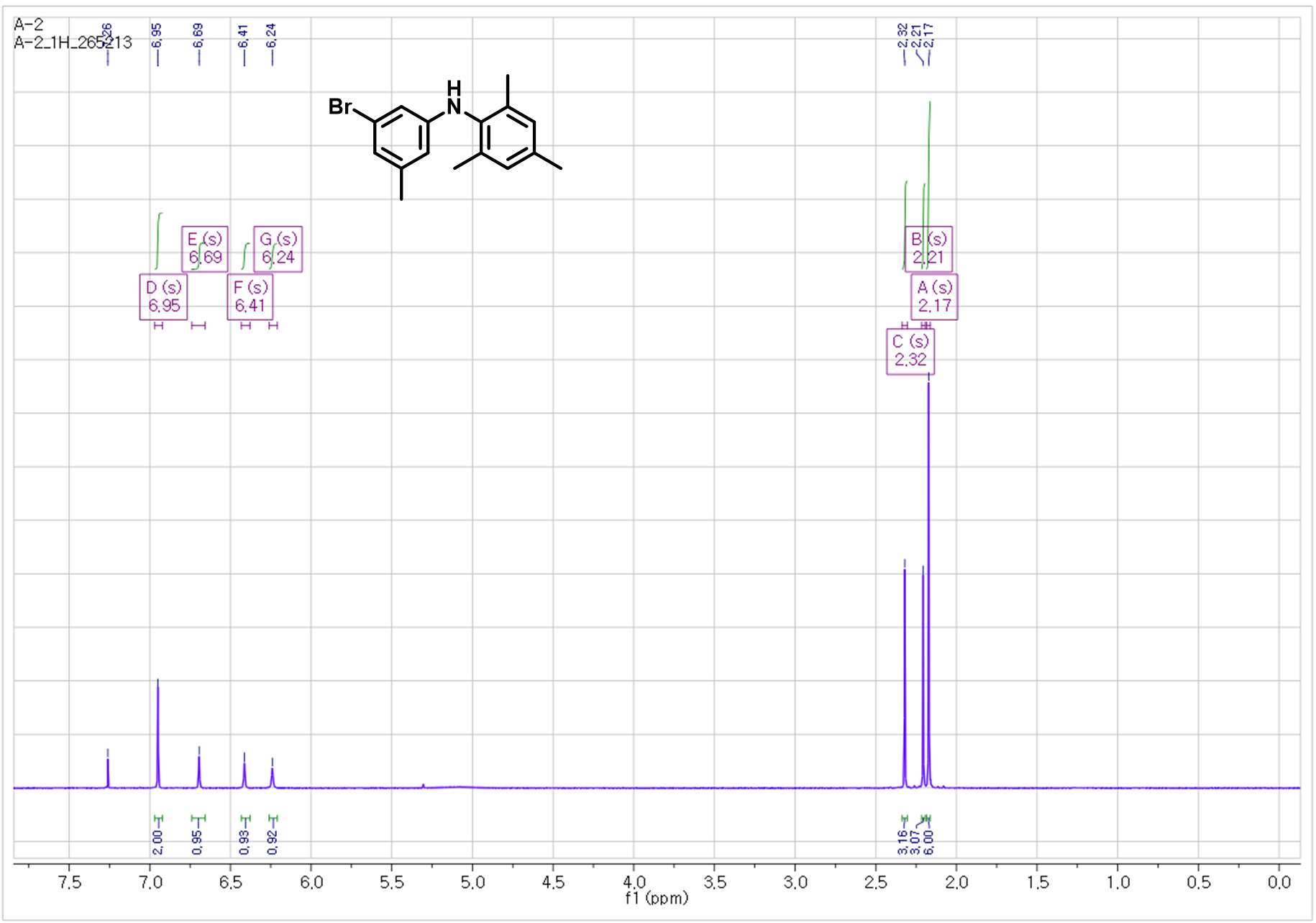
 **Figure S2.** ^1^H NMR spectrum of **A-2** (700 MHz, CDCl_3_)


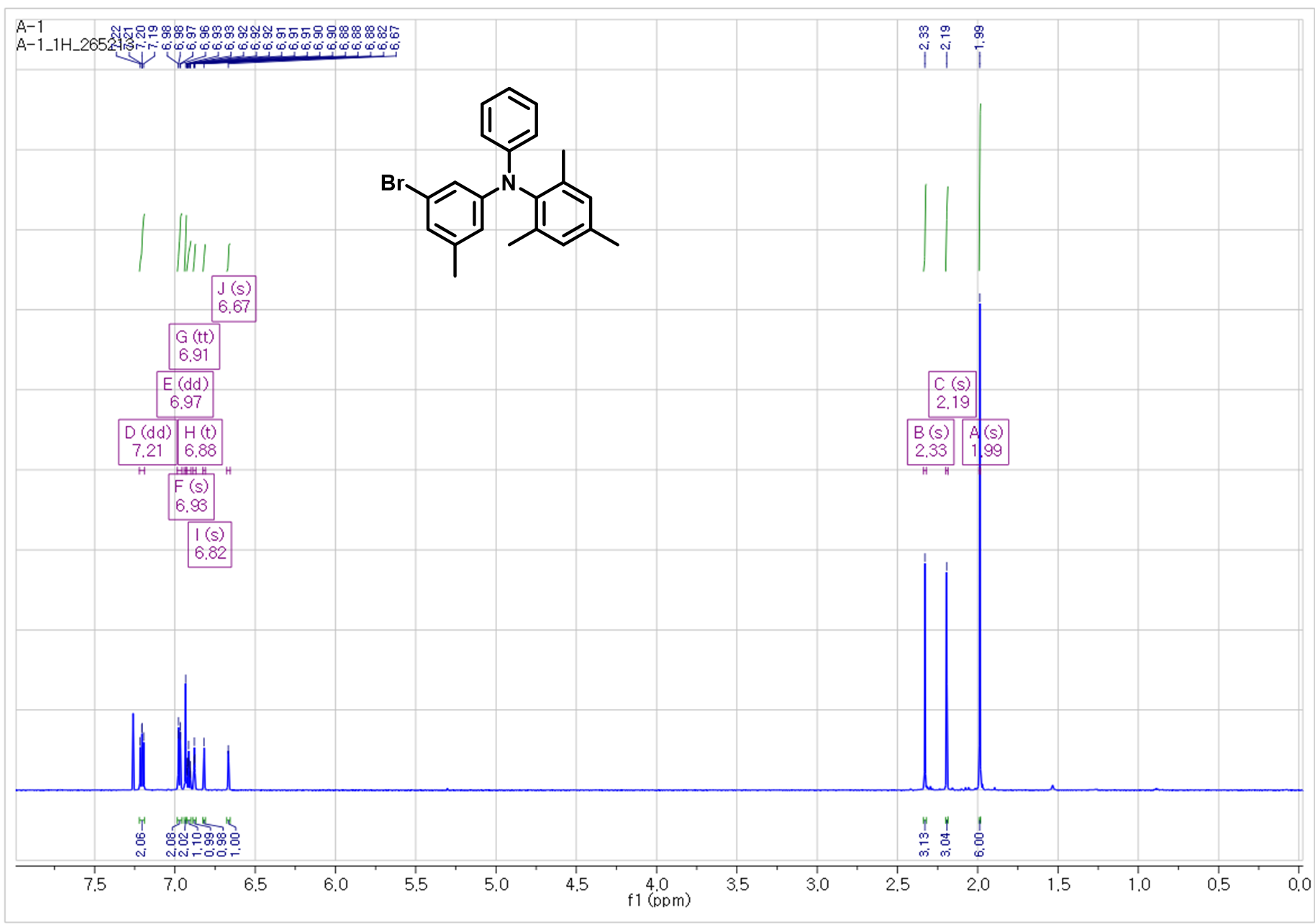
 **Figure S3.** ^1^H NMR spectrum of **A-1** (700 MHz, CDCl_3_)


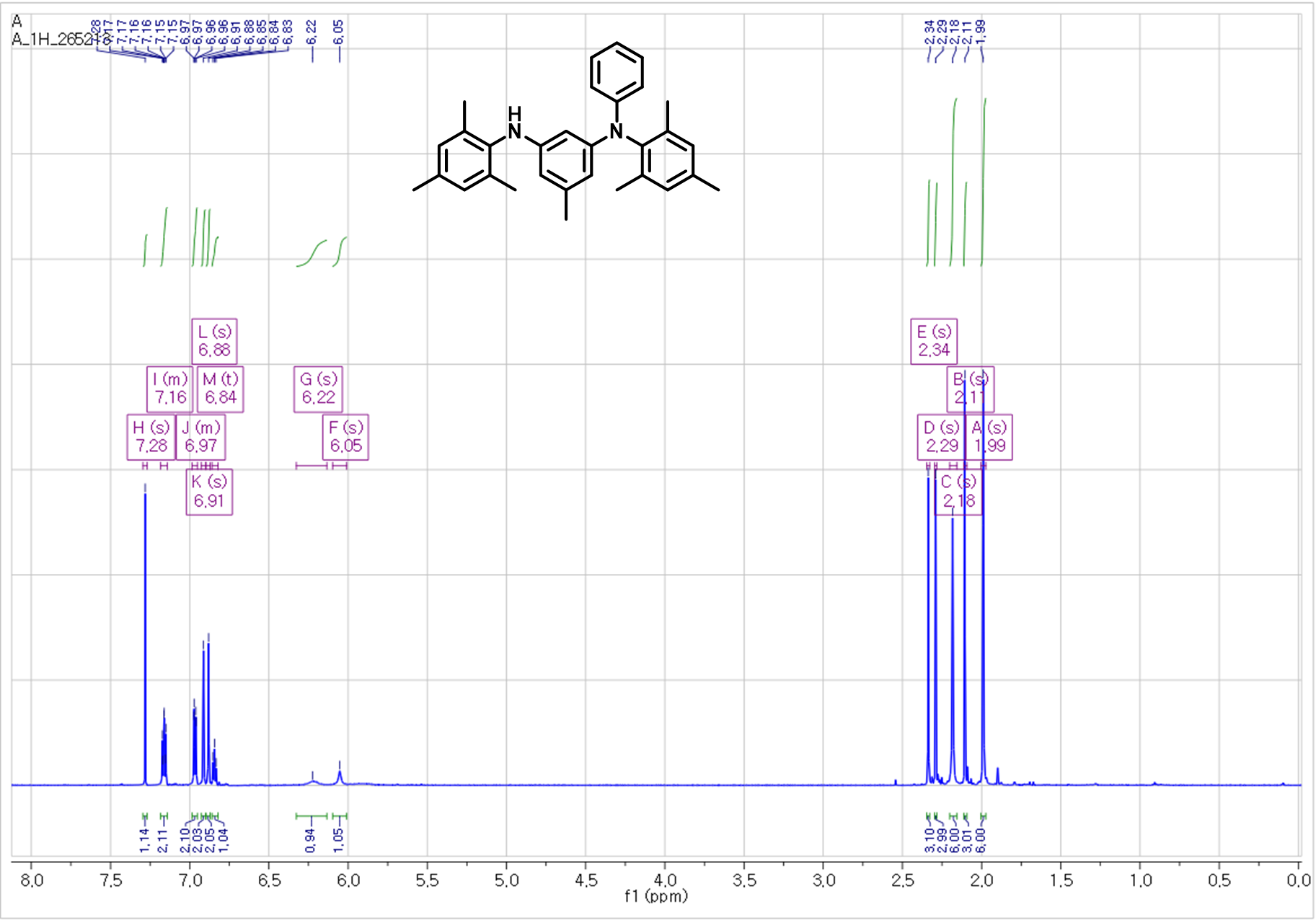
 **Figure S4.** ^1^H NMR spectrum of **A** (700 MHz, CDCl_3_)

**
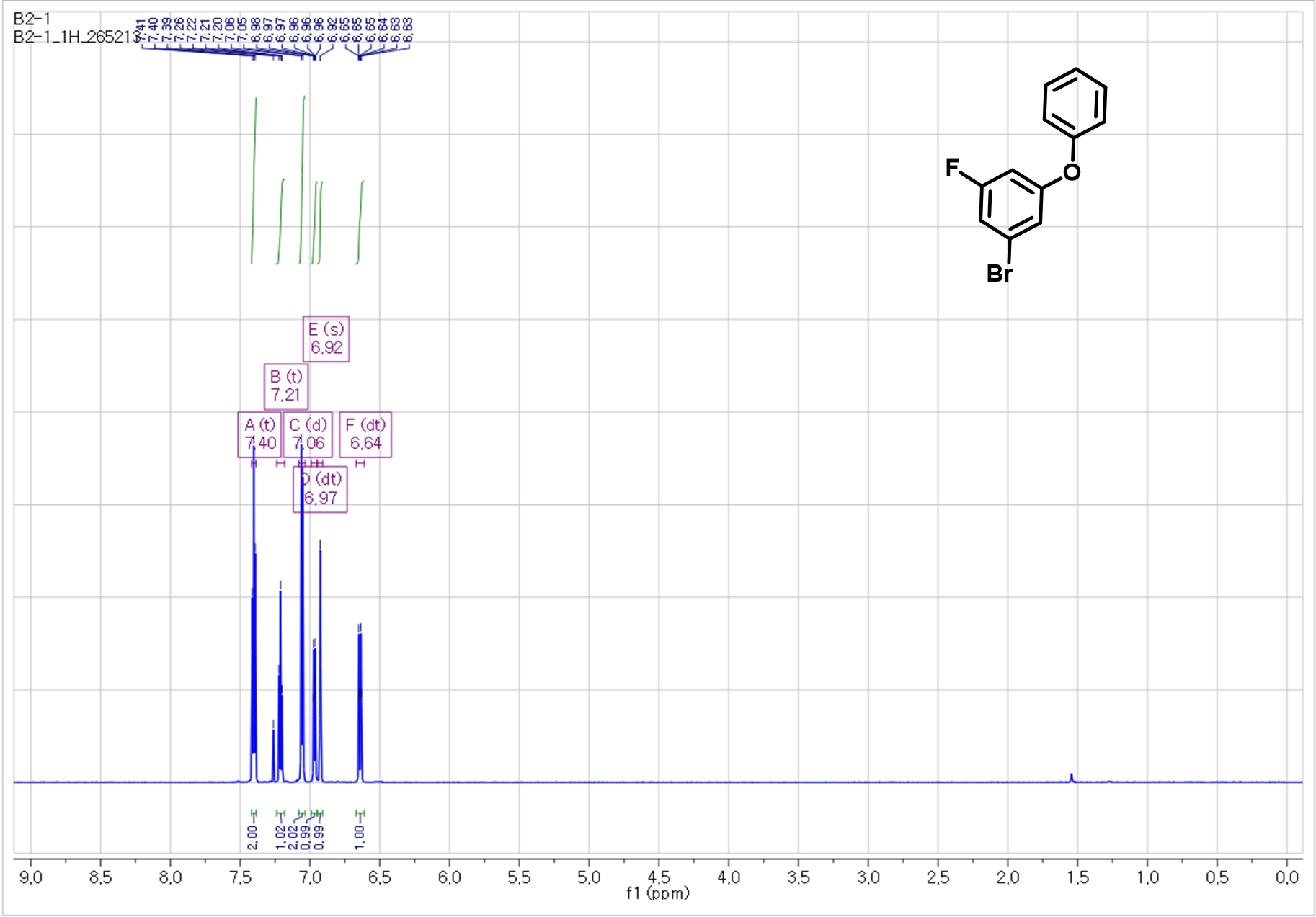
Figure S5.** ^1^H NMR spectrum of **B1-1** (700 MHz, CDCl_3_)


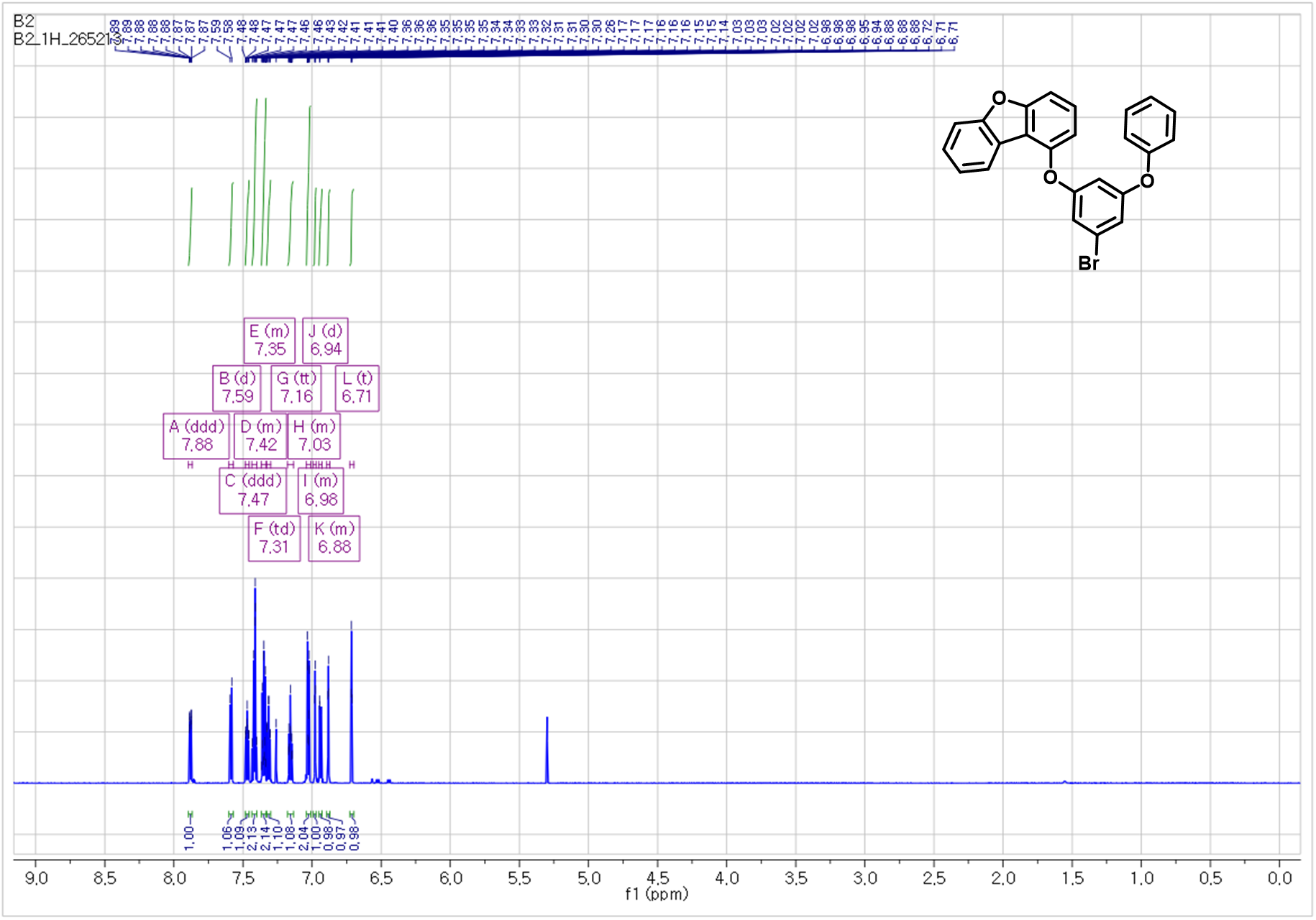
**Figure S6.** ^1^H NMR spectrum of **B1** (700 MHz, CDCl_3_)

**
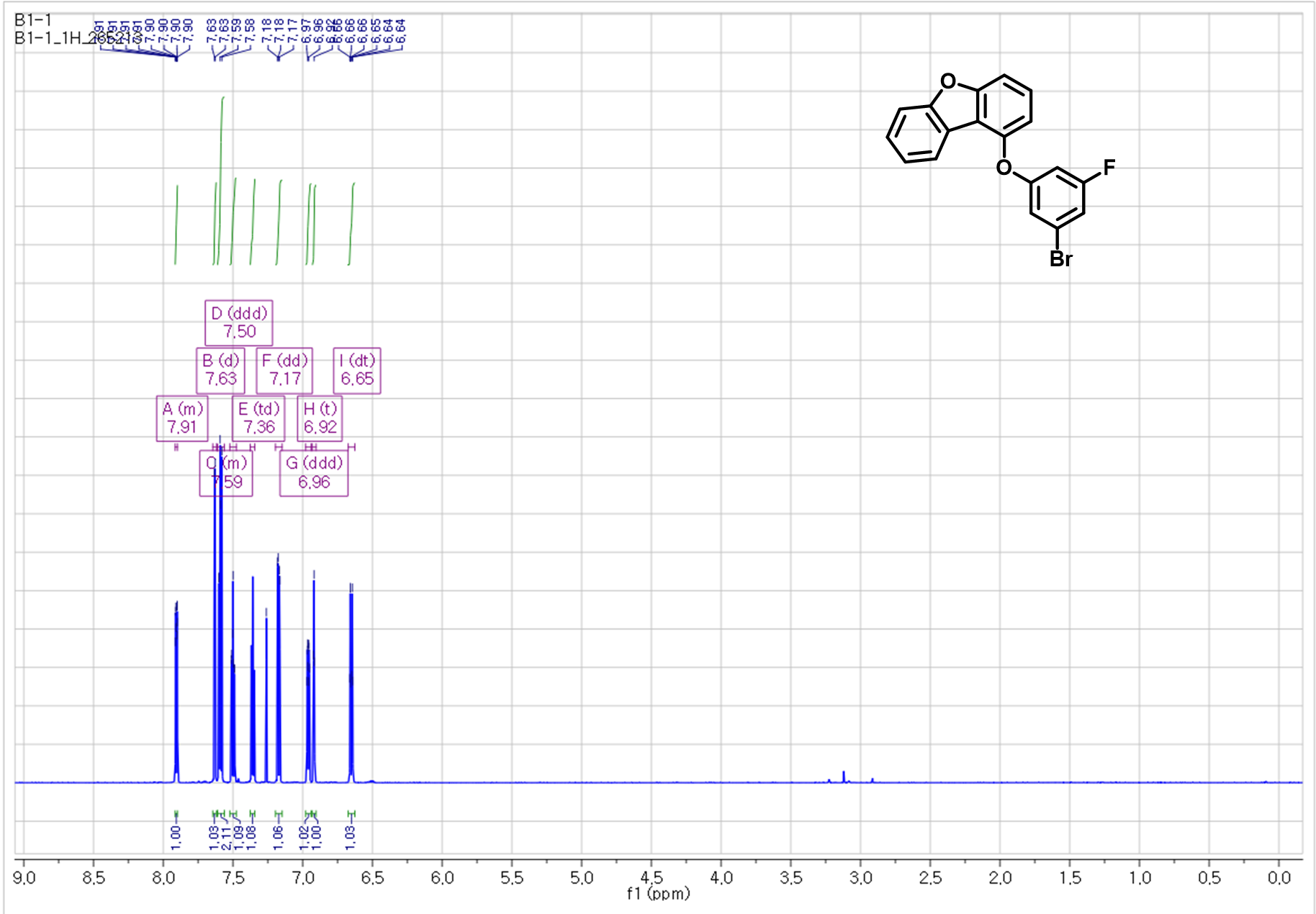
Figure S7.** ^1^H NMR spectrum of **B2-1** (700 MHz, CDCl_3_)


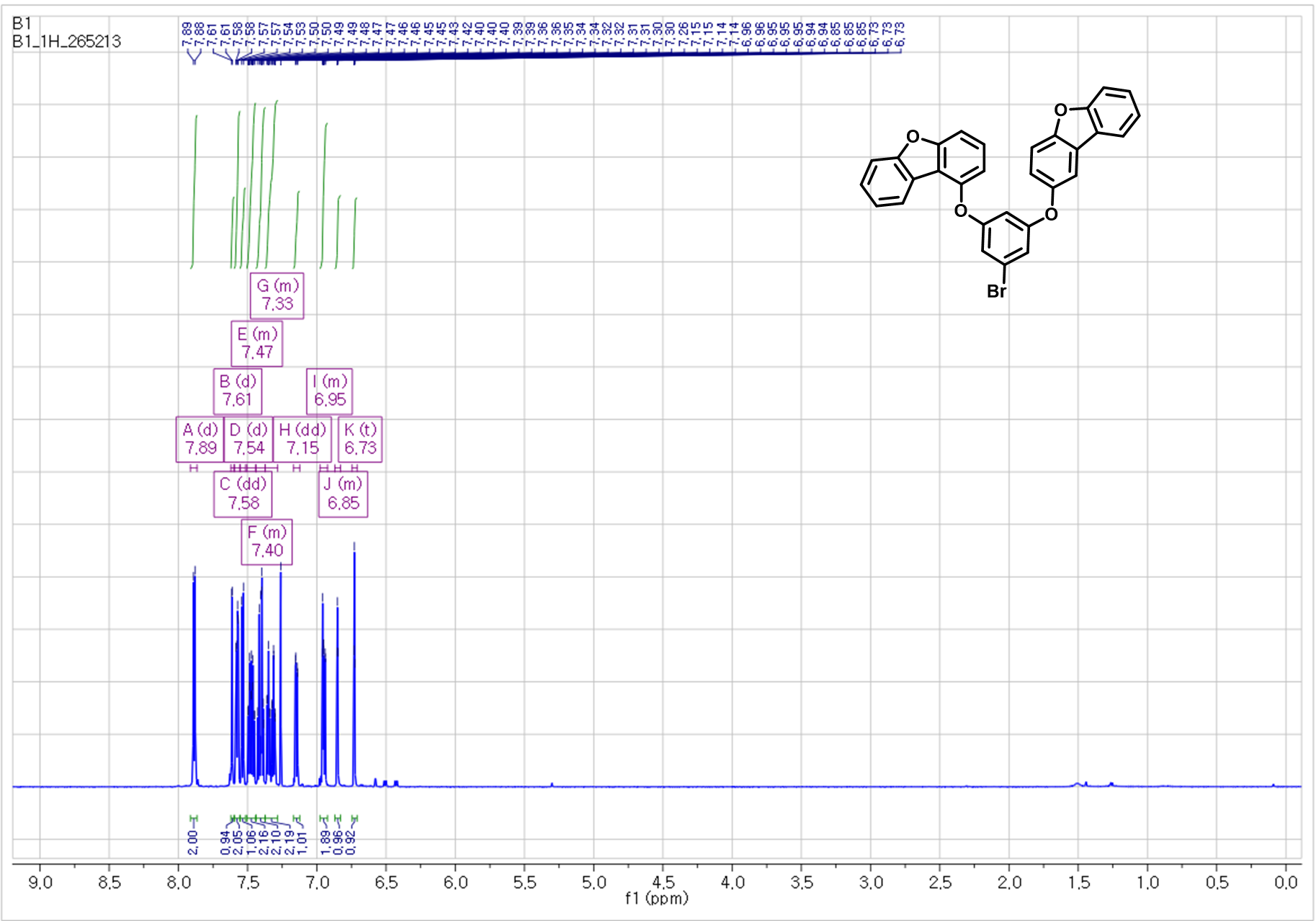
**Figure S8.** ^1^H NMR spectrum of **B2** (700 MHz, CDCl_3_)


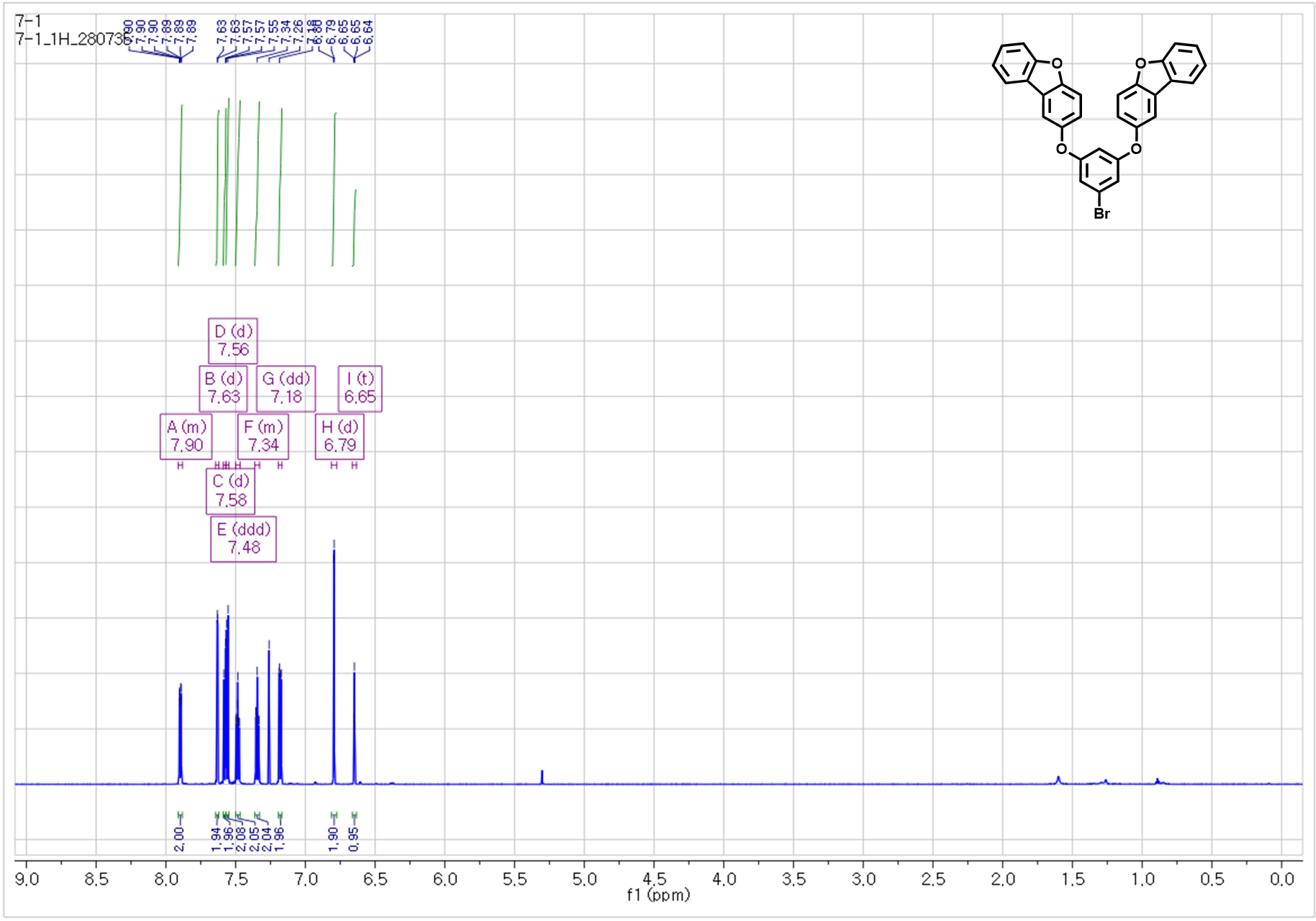
**Figure S9.** ^1^H NMR spectrum of **B3** (700 MHz, CDCl_3_)


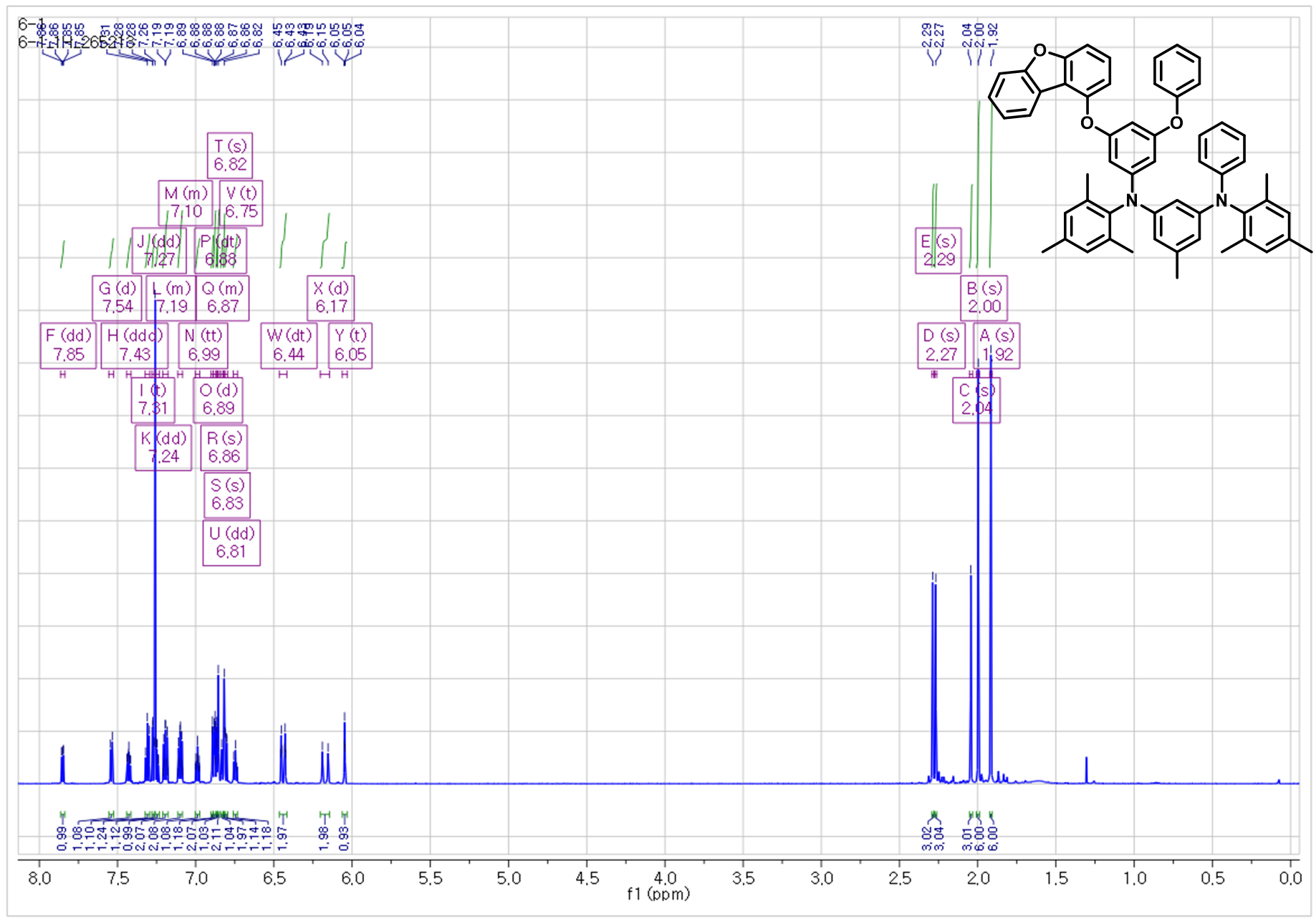
**Figure S10.** ^1^H NMR spectrum of **AB1** (700 MHz, CDCl_3_)


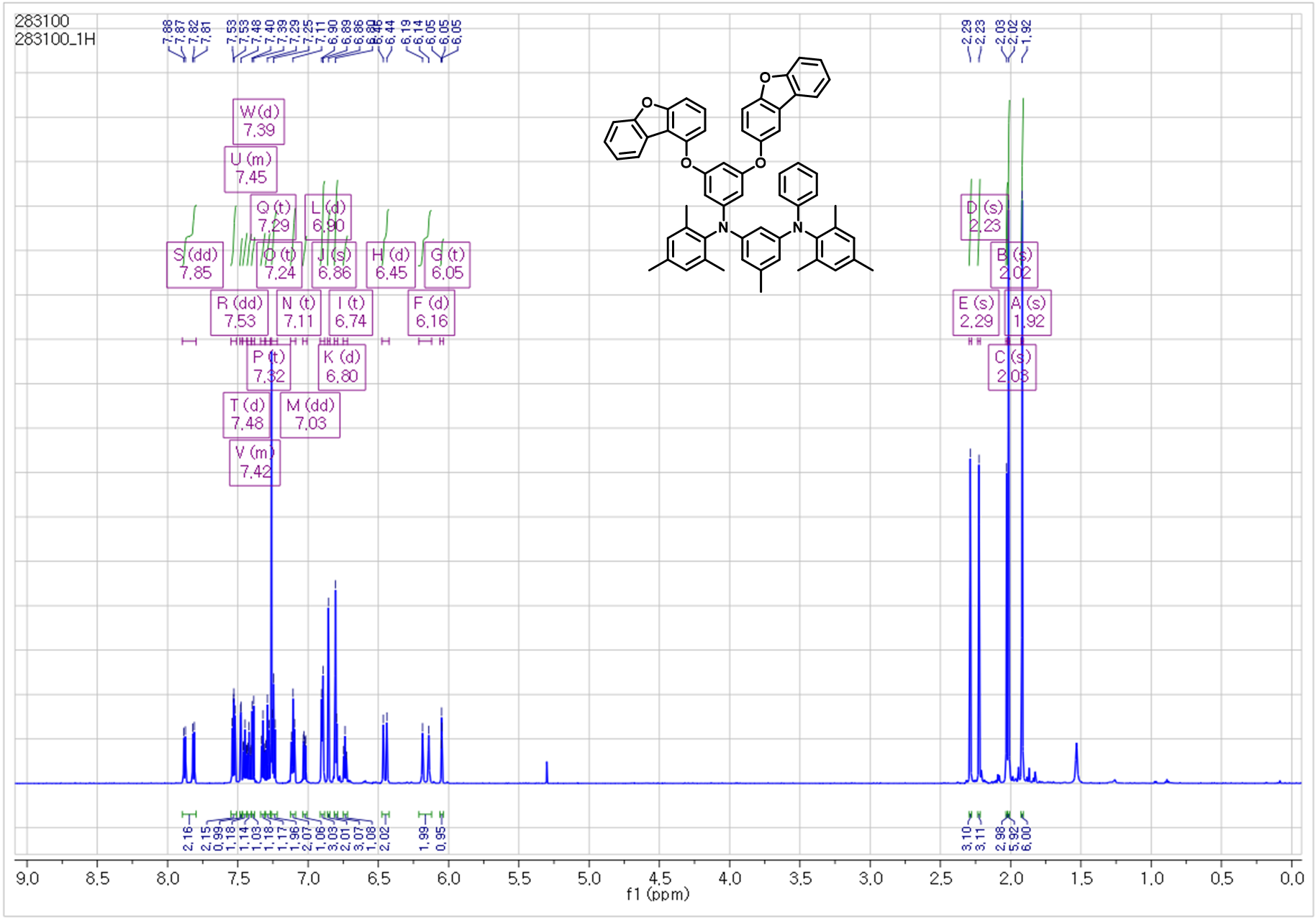
**Figure S11.** ^1^H NMR spectrum of **AB2** (700 MHz, CDCl_3_)


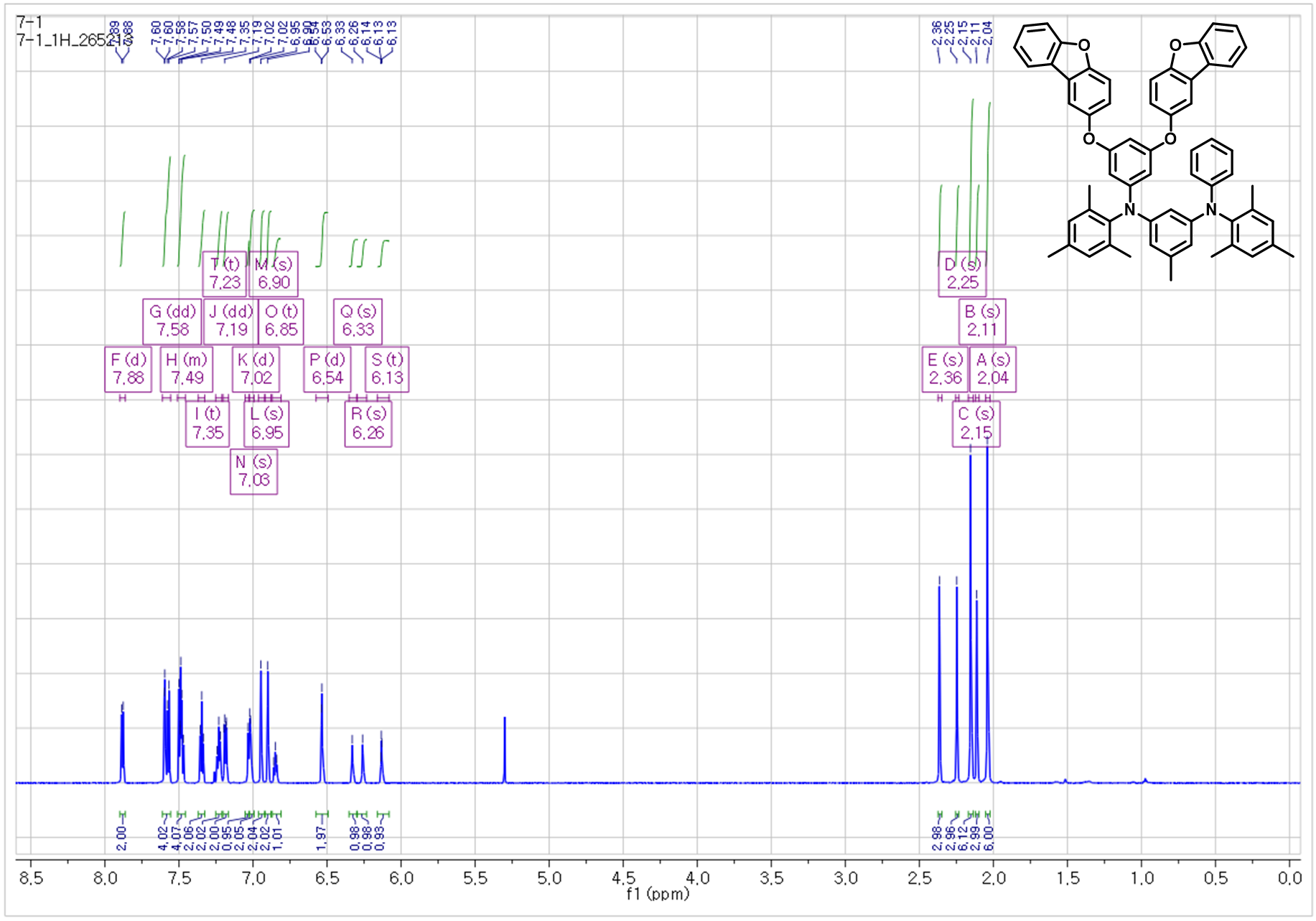
**Figure S12.** ^1^H NMR spectrum of **AB3** (700 MHz, CDCl_3_)

**
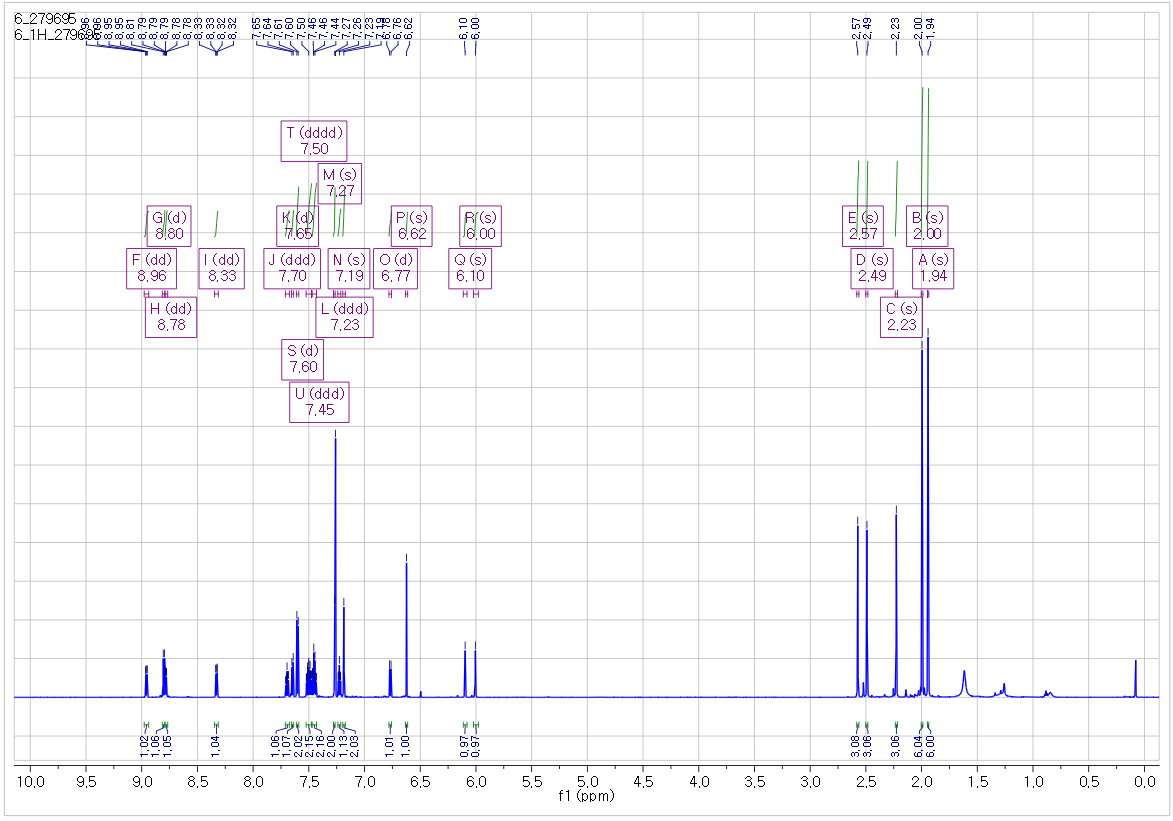

Figure S13.** ^1^H NMR spectrum of ***bf*DOB-BN1** (700 MHz, CDCl_3_)


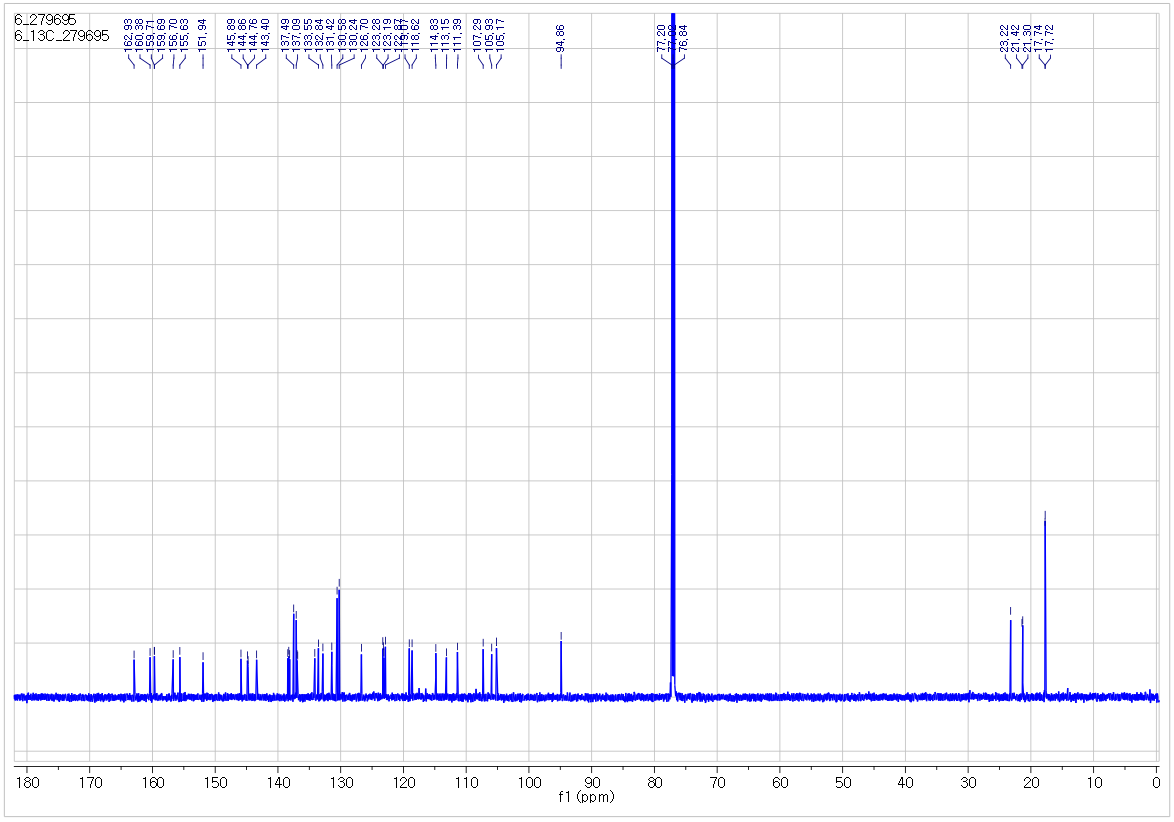
 **Figure S14.** ^13^C NMR spectrum of ***bf*DOB-BN1** (700 MHz, CDCl_3_)*
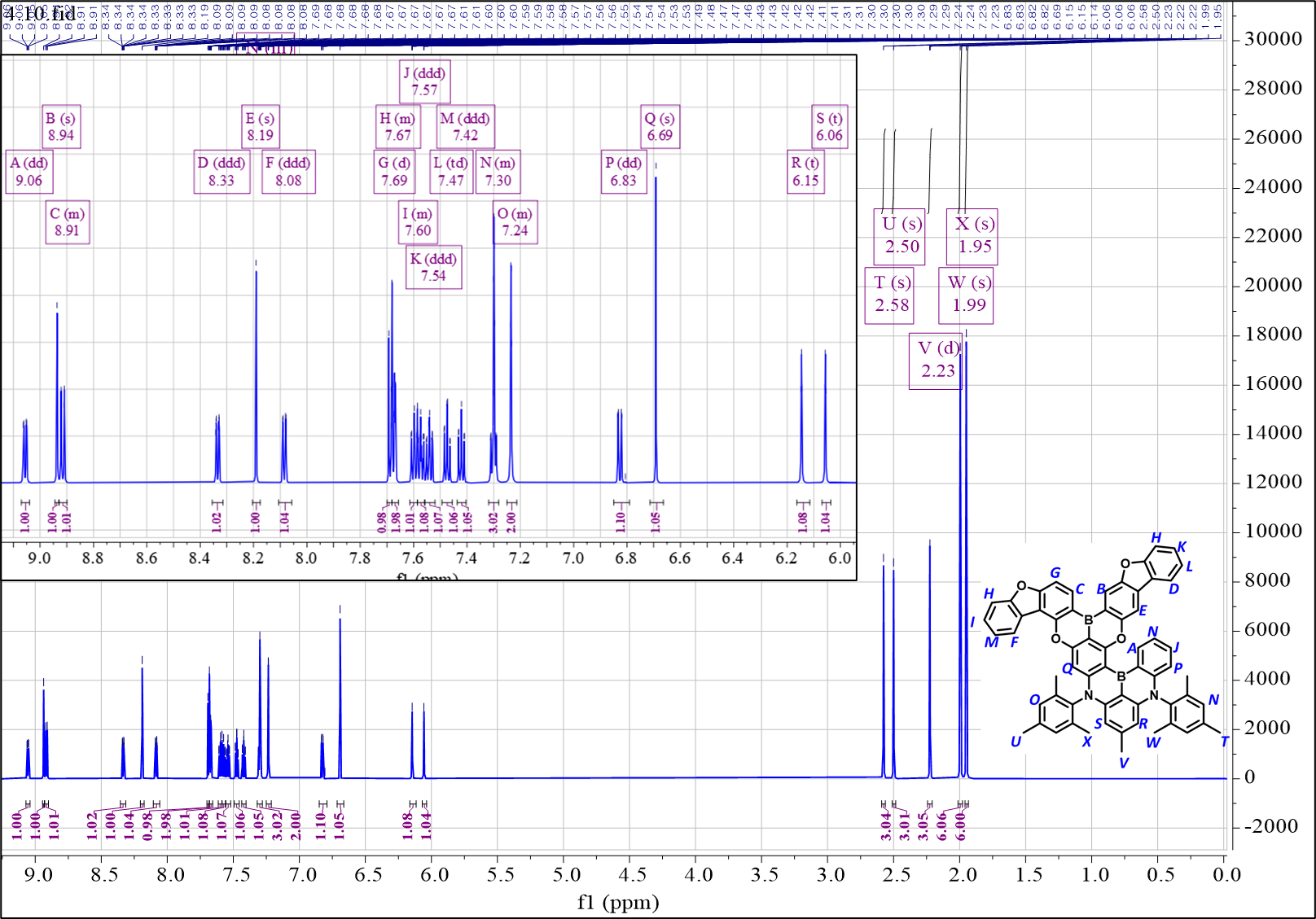
* **Figure S15.** ^1^H NMR spectrum of ***bf*DOB-BN2** (700 MHz, CD_2_Cl_2_)
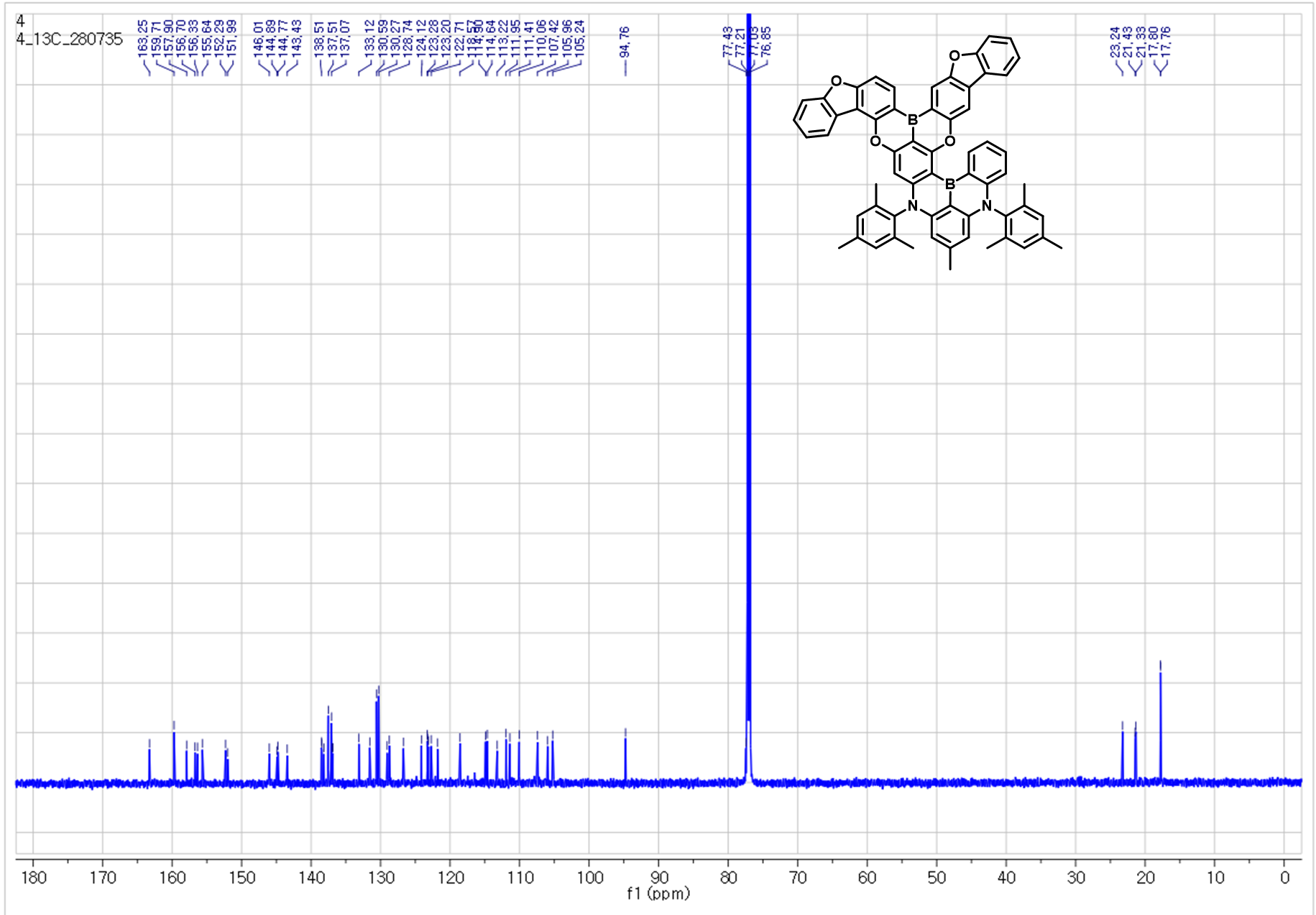

**Figure S16.** ^13^C NMR spectrum of ***bf*DOB-BN2** (700 MHz, CDCl_3_)

**
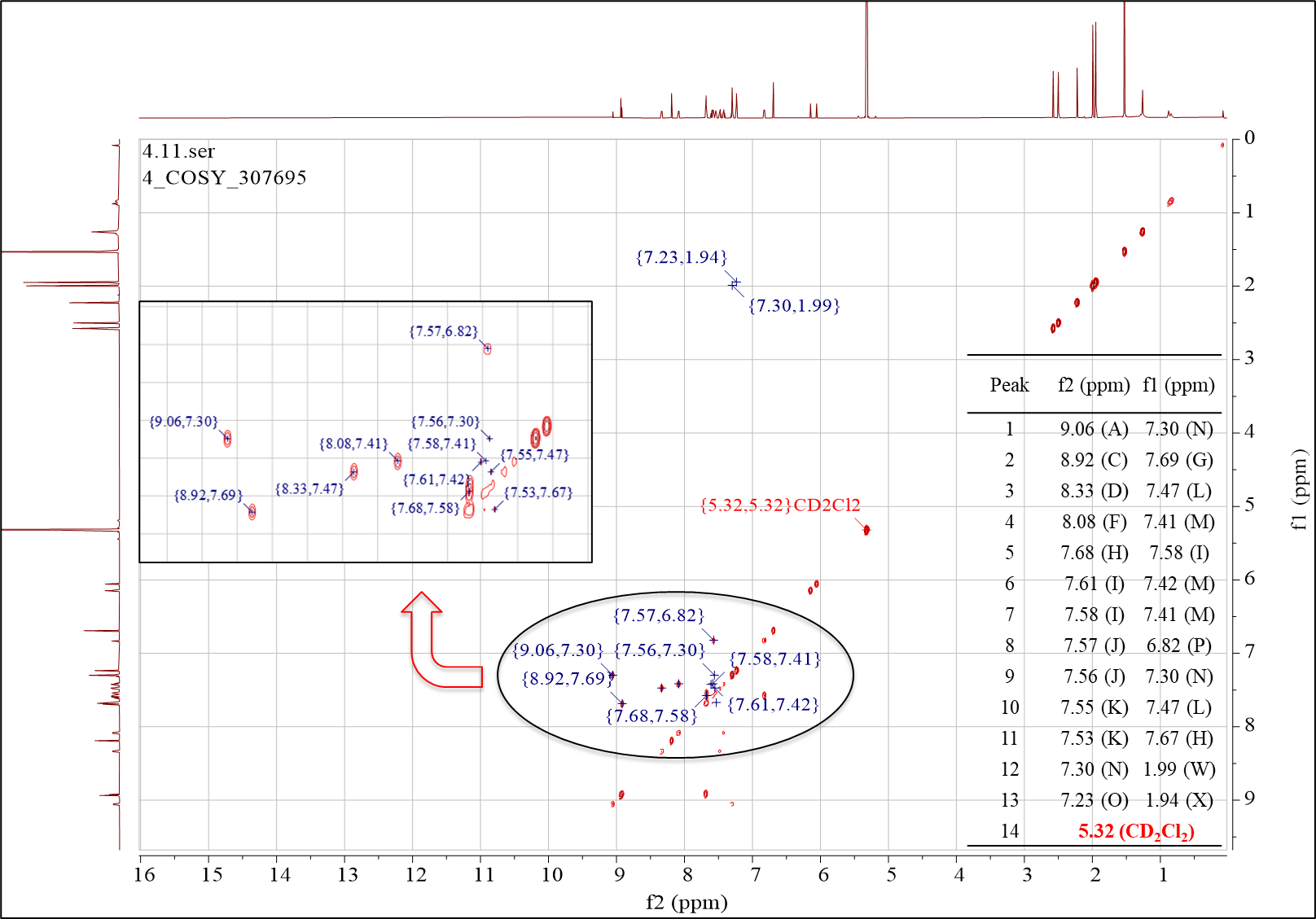
Figure S17.** ¹H–¹H COSY NMR spectra of ***bf*DOB-BN2** (700 MHz, CD₂Cl₂).


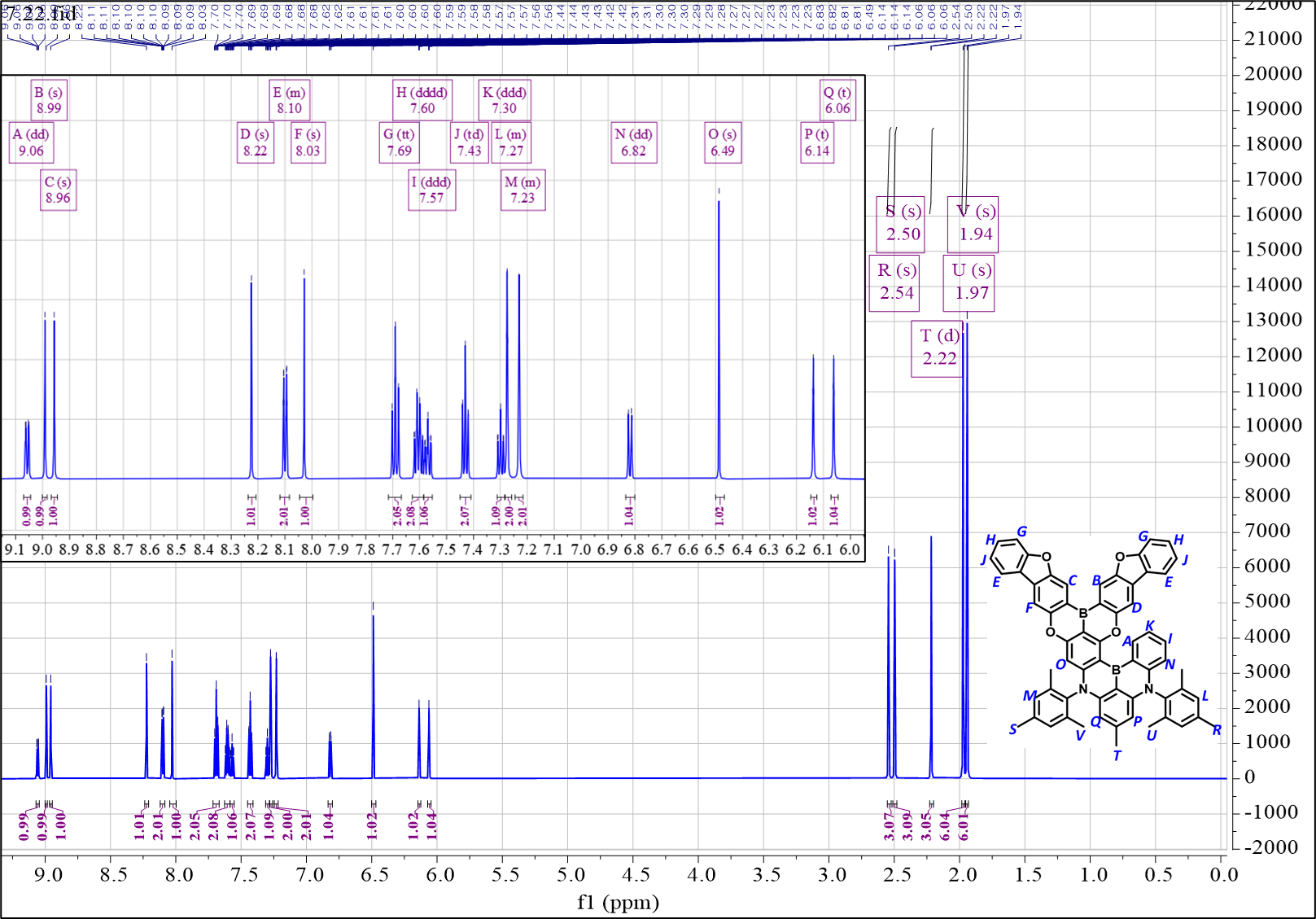


**Figure S18.** ^1^H NMR spectrum of ***bf*DOB-BN3** (700 MHz, CD_2_Cl_2_)
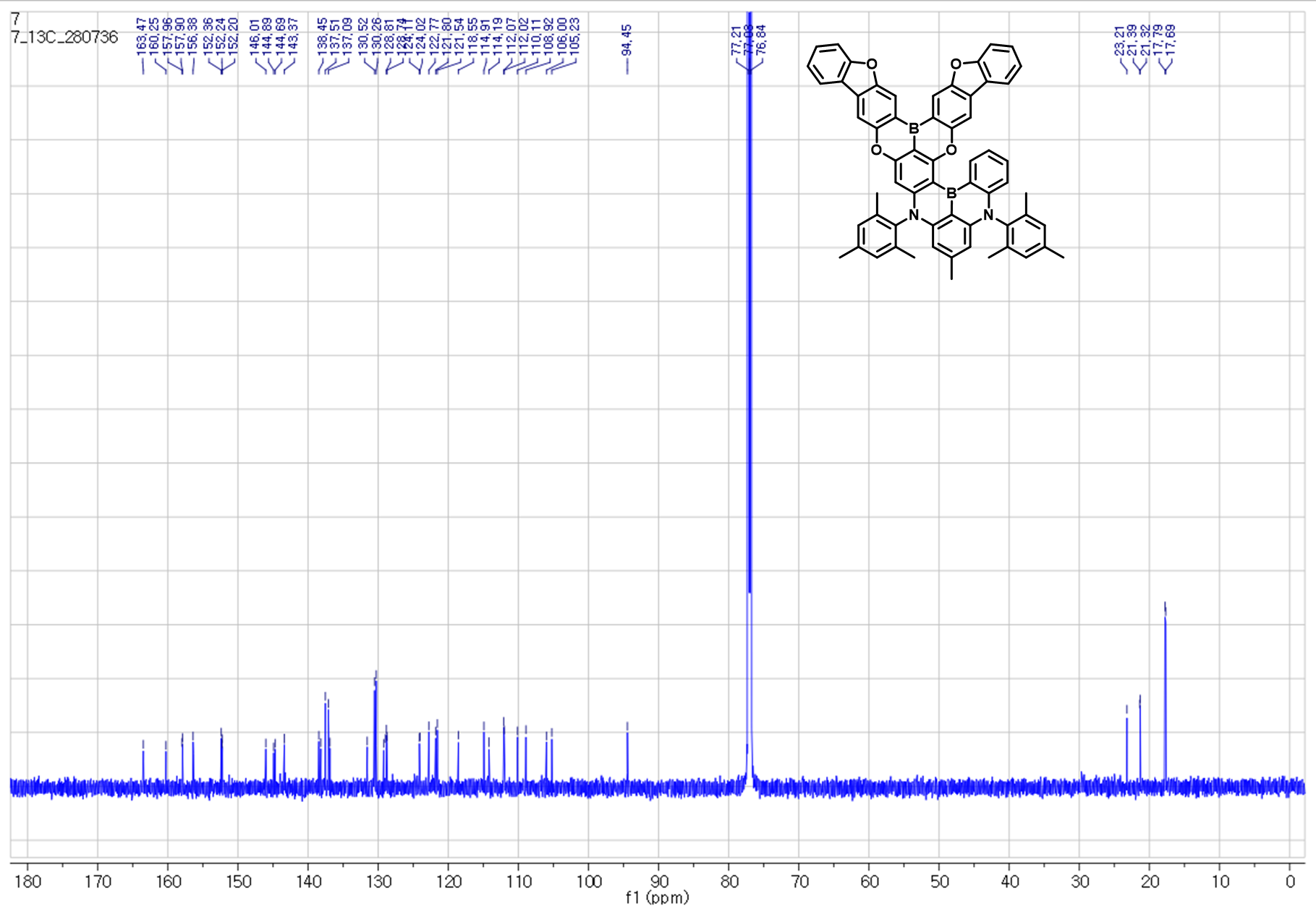


**Figure S19.** ^13^C NMR spectrum of ***bf*DOB-BN3** (700 MHz, CDCl_3_)

**
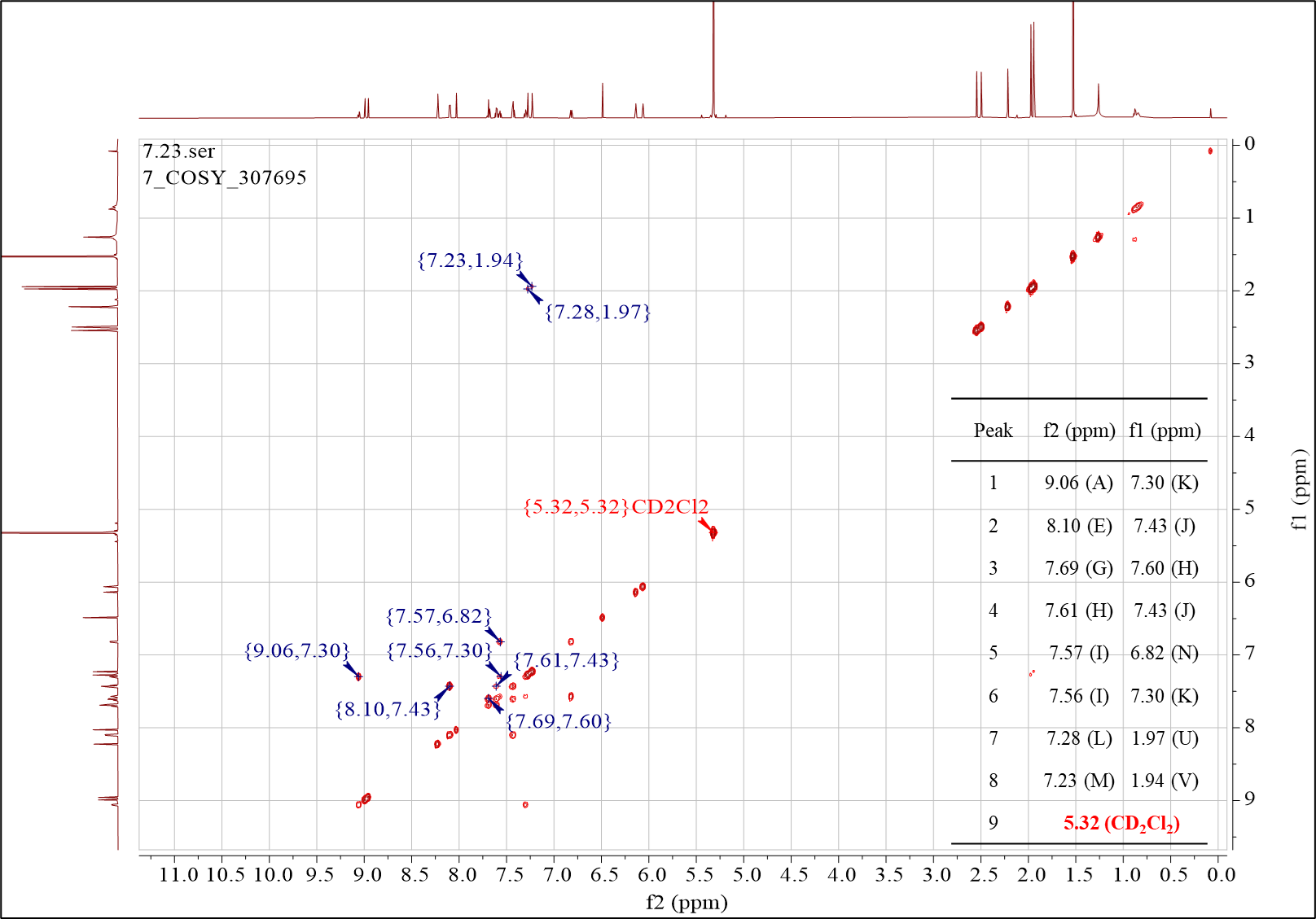
**
**Figure S20.** ¹H–¹H COSY NMR spectra of ***bf*DOB-BN3** (700 MHz, CD₂Cl₂).


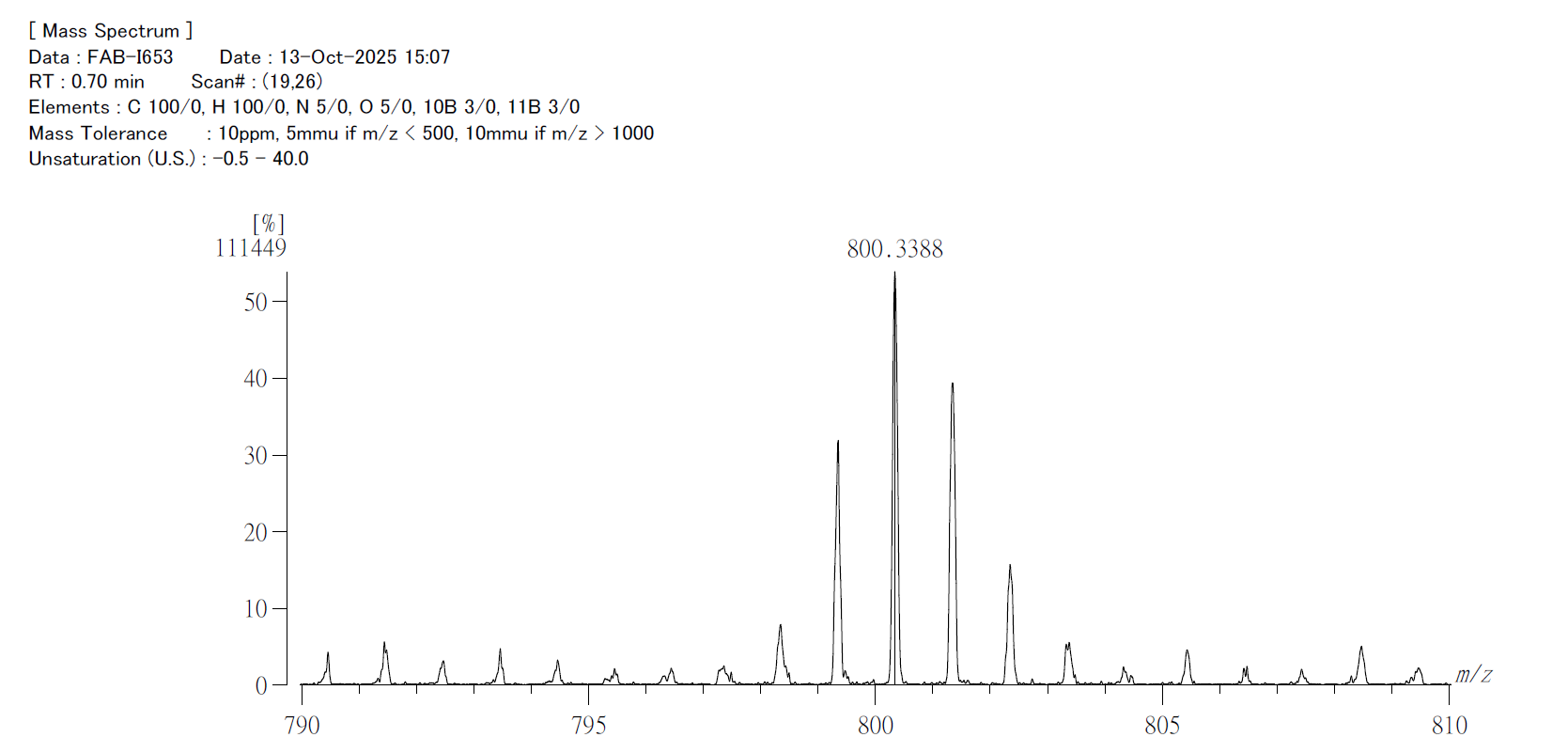


**Figure S21.** HRMS data of ***bf*DOB-BN1**


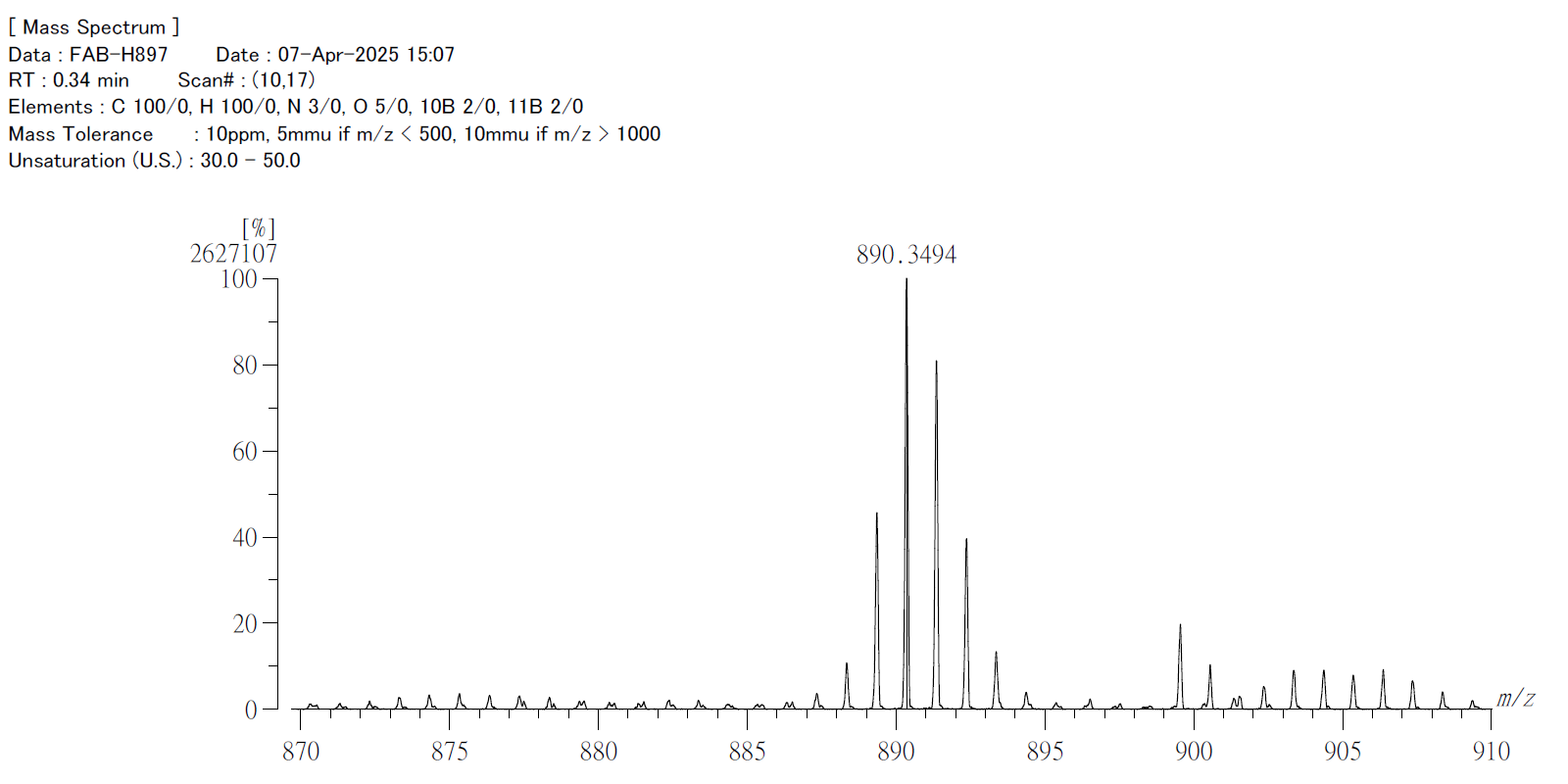


**Figure S22.** HRMS data of ***bf*DOB-BN2**


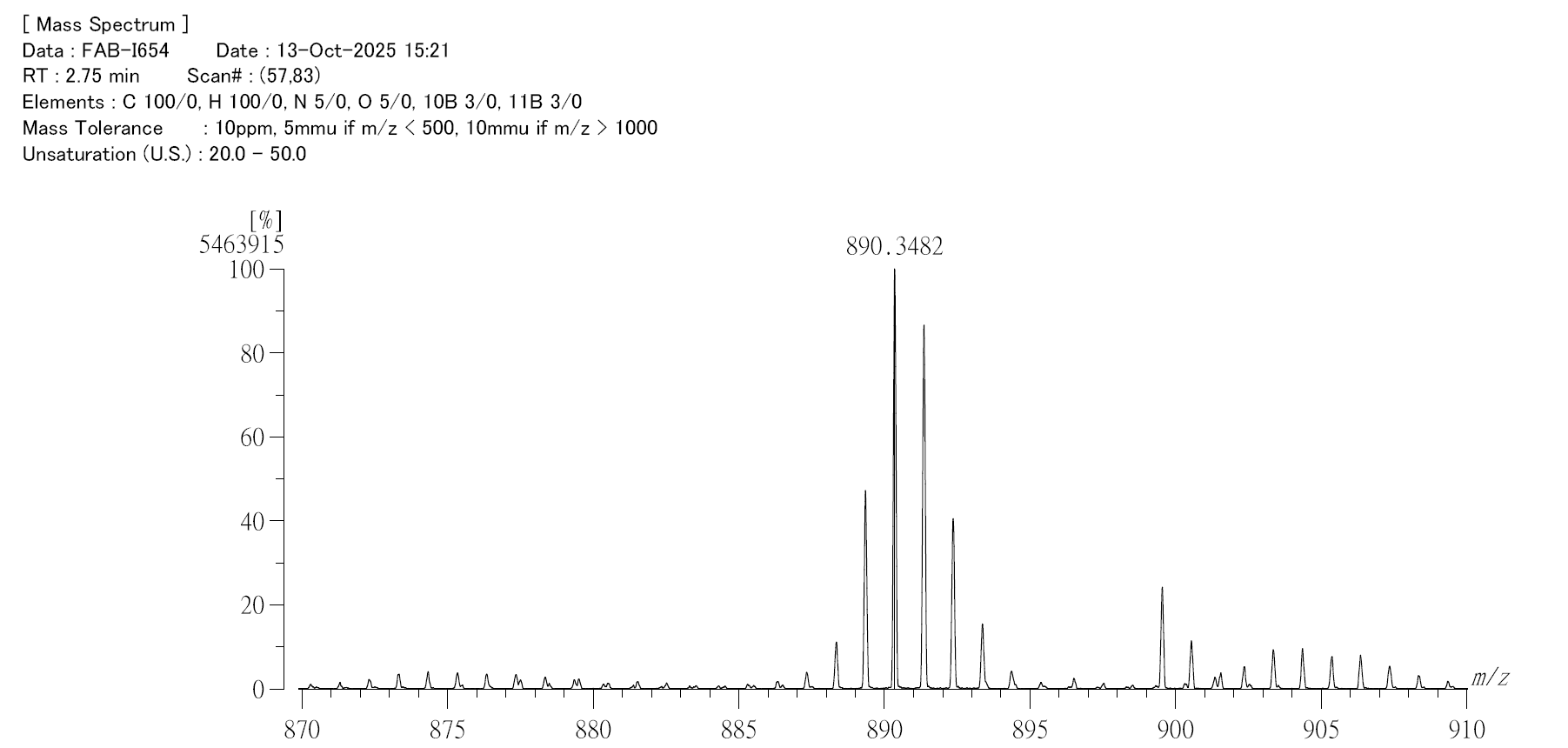


**Figure S23.** HRMS data of ***bf*DOB-BN3**


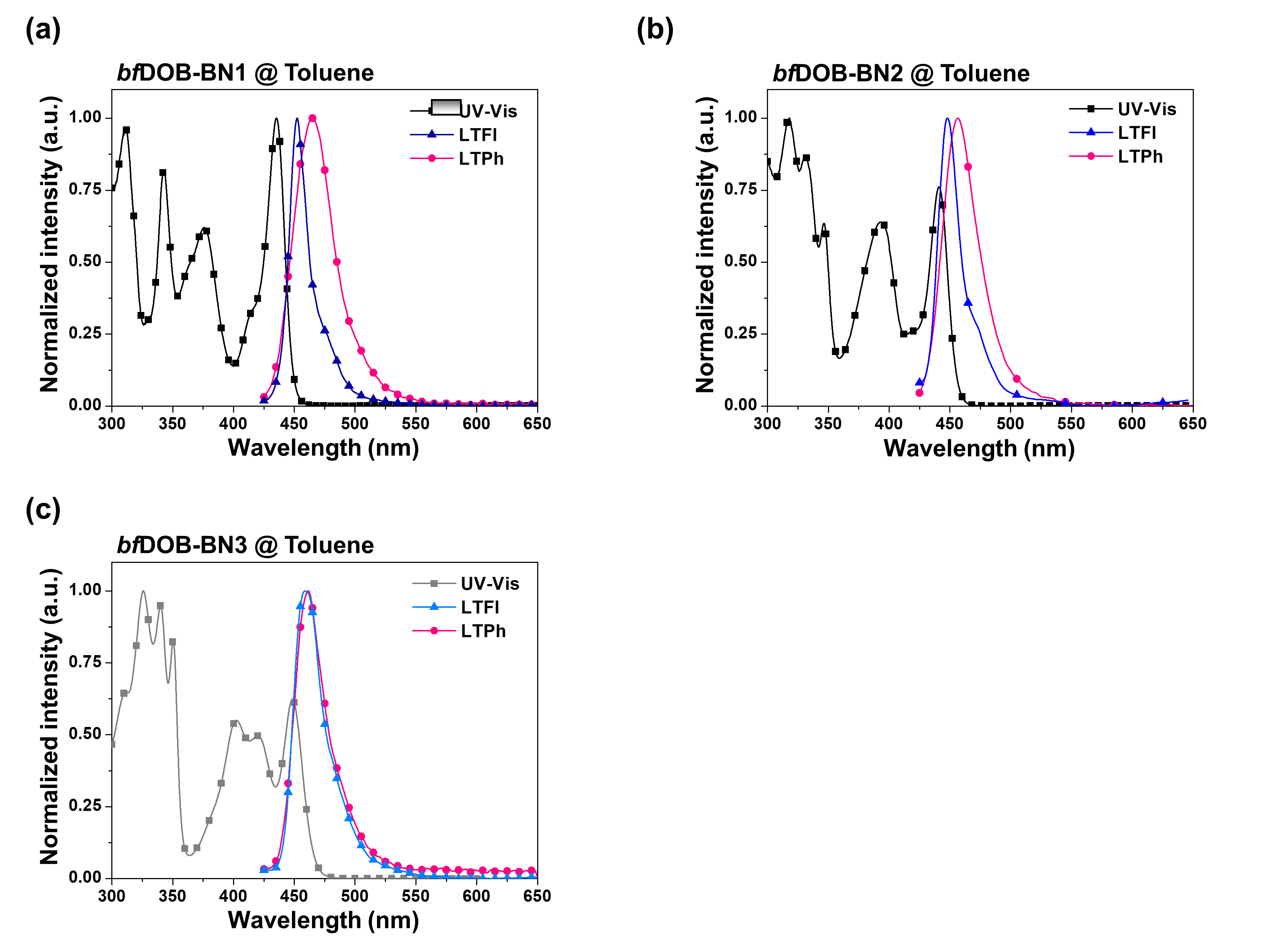


**Figure S24**. UV-Vis and photoluminescence spectra of (a) ***bf*DOB-BN1**, (b) ***bf*DOB-BN2** and (c) ***bf*DOB-BN3** in 10^-5^ M toluene at 77K. LTFl and LTPh represent low-temperature fluorescence and low-temperature phosphorescence, respectively.


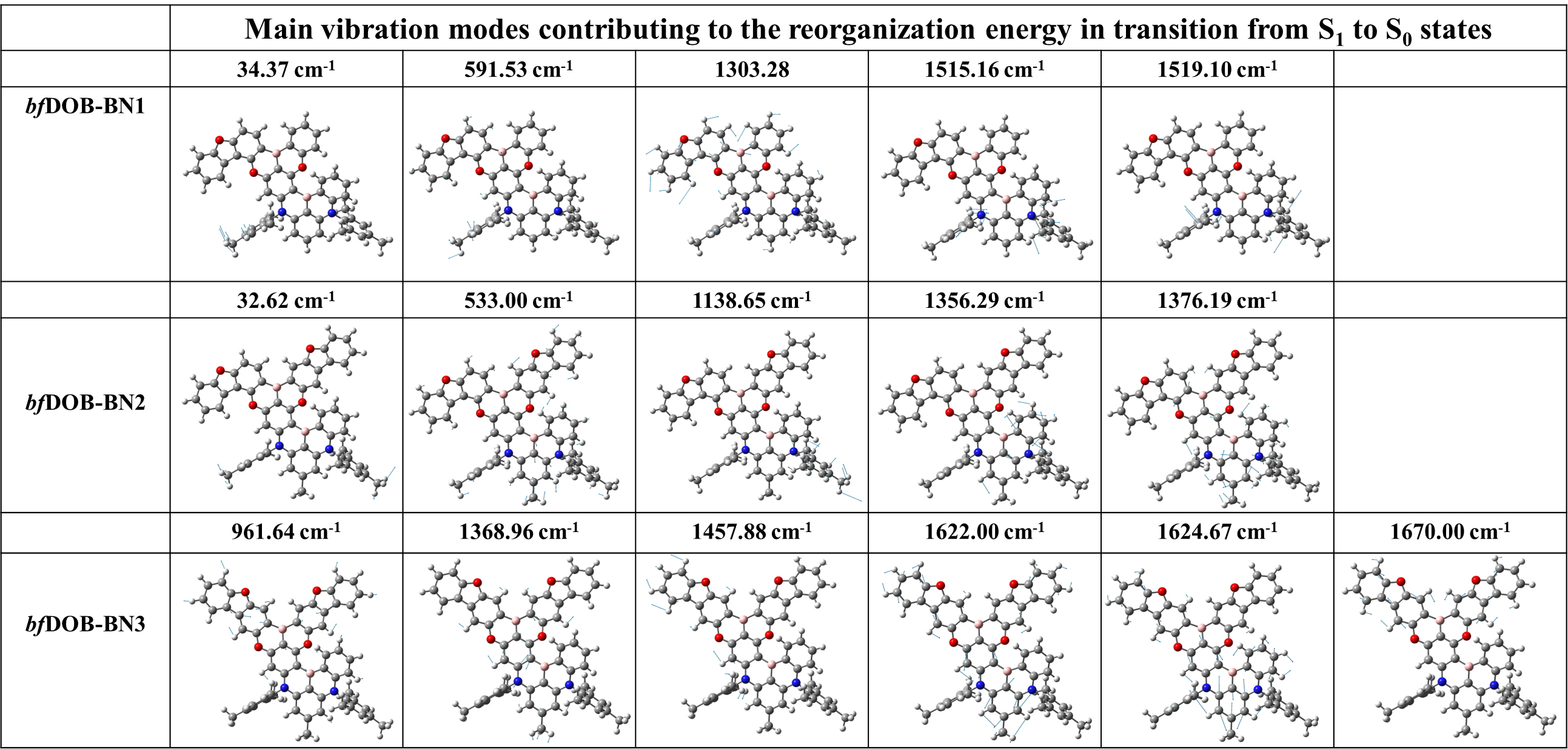
**Figure S25.** Reorganization energy analysis of ***bf*DOB-BN1**, ***bf*DOB-BN2** and ***bf*DOB-BN3** and the dominant vibrational modes contributing to the S₁ → S₀ transition.

**
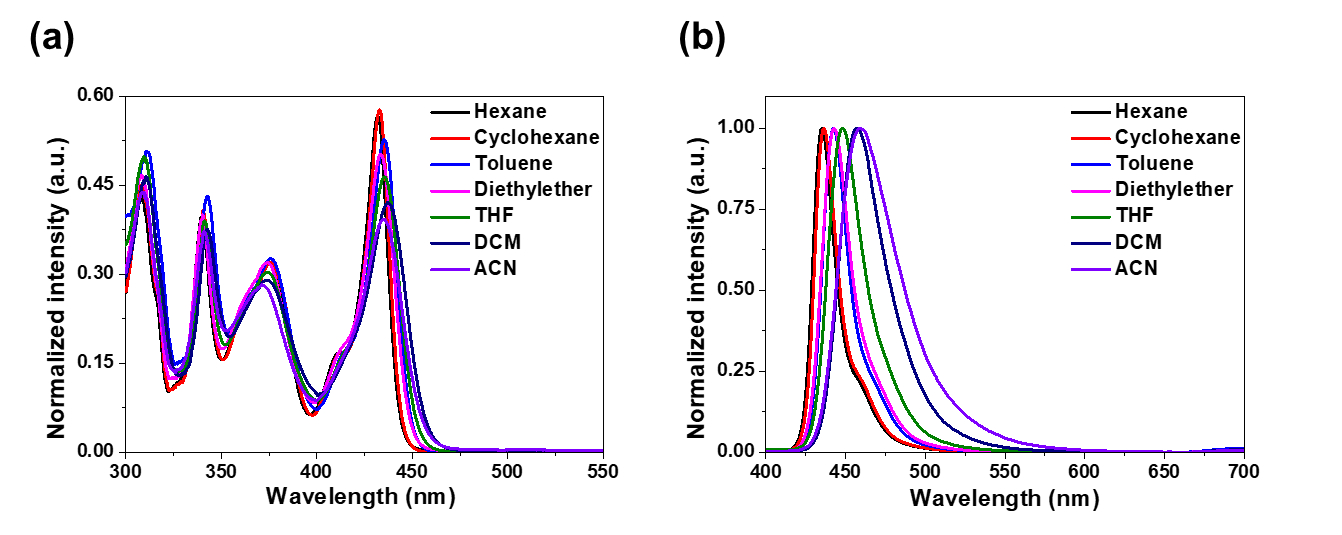
Figure S26. (a)**UV–vis absorption and (b)photoluminescence spectra of ***bf*DOB-BN1** measured in various solvents (10^-5^ M, 298 K).


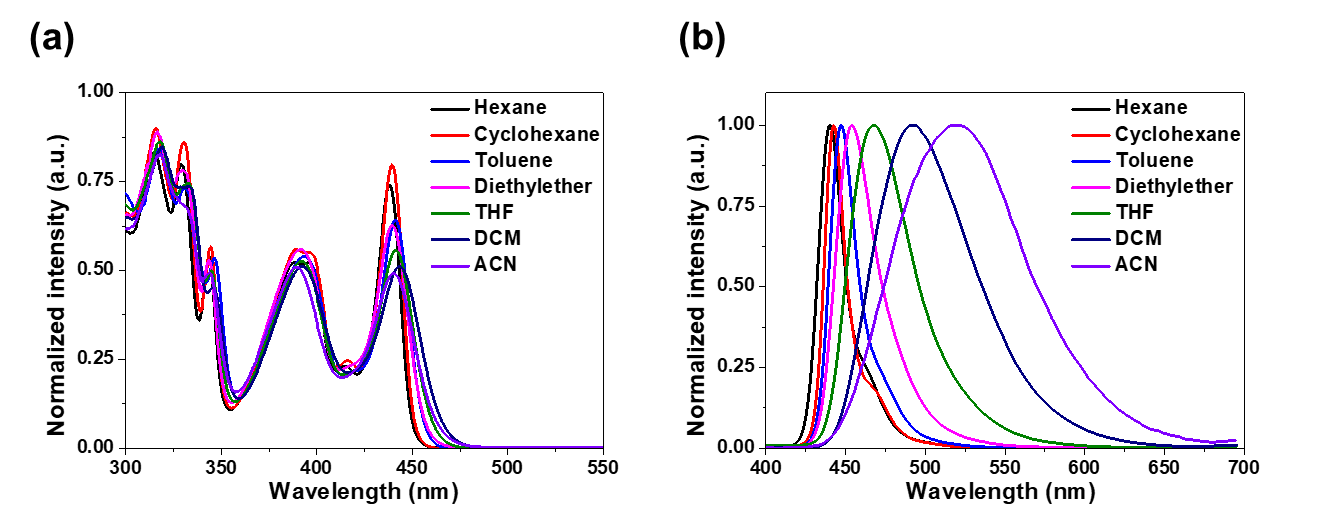


**Figure S27.** (a)UV–vis absorption and (b)photoluminescence spectra of ***bf*DOB-BN2** measured in various solvents (10^-5^ M, 298 K).


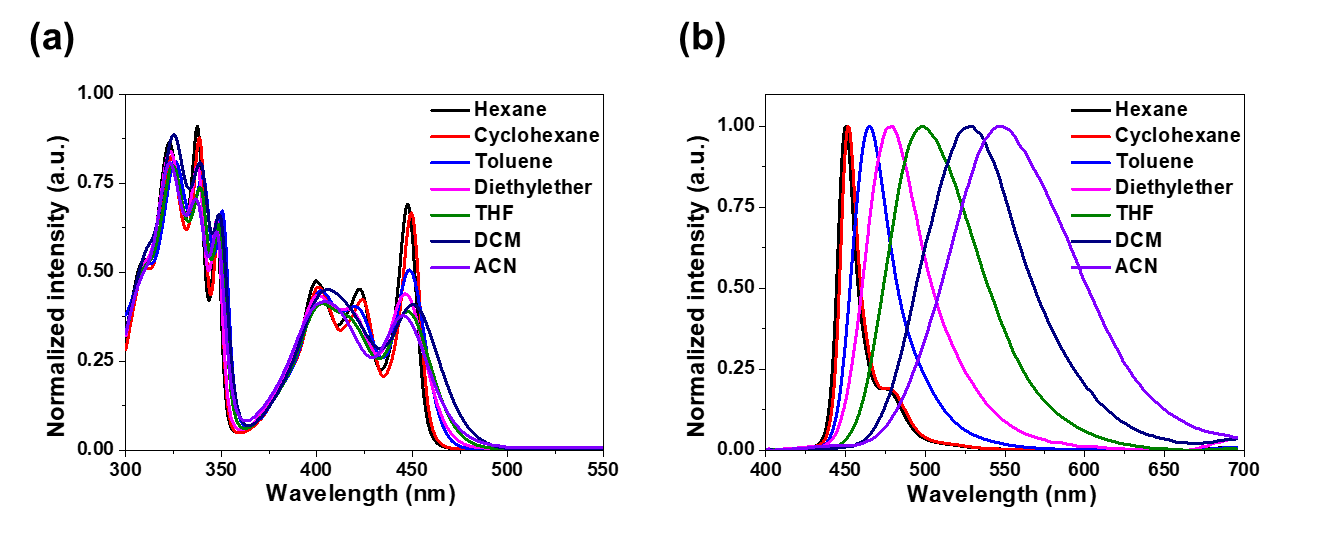


**Figure S28. (a)**UV–vis absorption and (b)photoluminescence spectra of ***bf*DOB-BN3** measured in various solvents (10^-5^ M, 298 K).


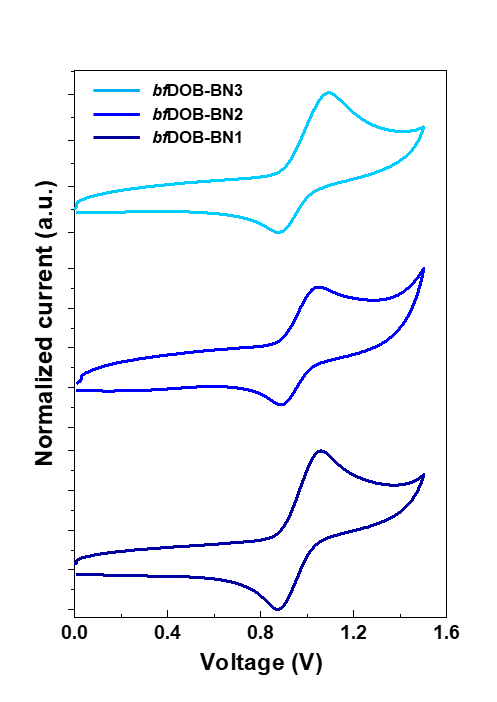
**Figure S29.** Cyclic voltammograms of ***bf*DOB-BN1**, ***bf*DOB-BN2** and ***bf*DOB-BN3**. The oxidation onset potential of each compound was obtained to estimate the HOMO levels.

**
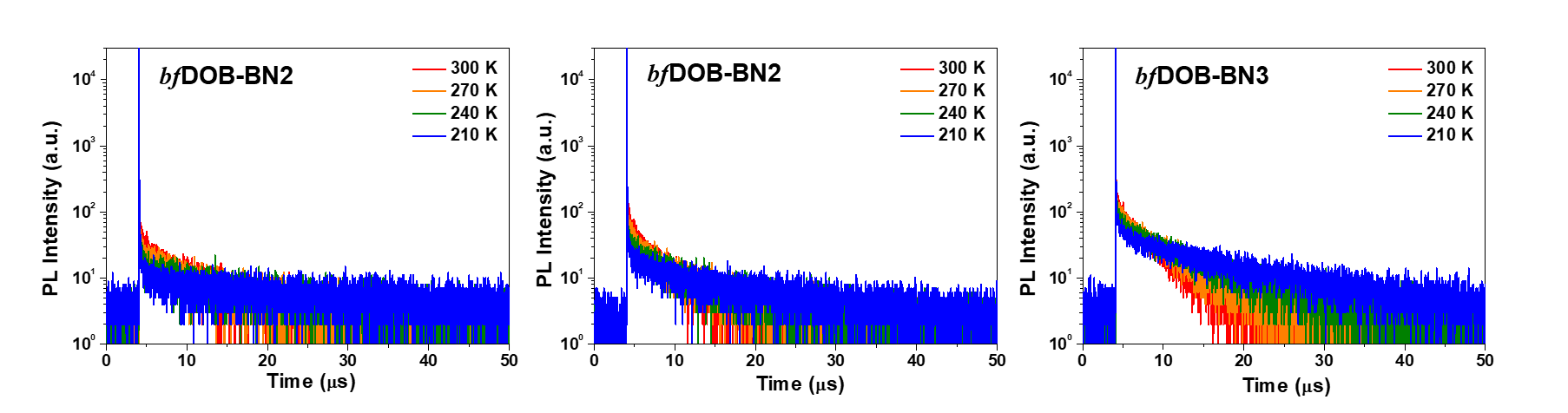
**

**Figure S30**. Temperature-dependent transient PL decay of the delayed component (1.0 wt% doped in 3-CzPB film).

**Figure S31.** Modified Arrhenius plots of ln(*k_rISC,eff_ꞏT^1/2^*) versus 1/T for ***bf*DOB-BN1**, ***bf*DOB-BN2**, and ***bf*DOB-BN3**.

**Figure S32**. TGA thermogram of ***bf*DOB-BN1**, ***bf*DOB-BN2** and ***bf*DOB-BN3**.


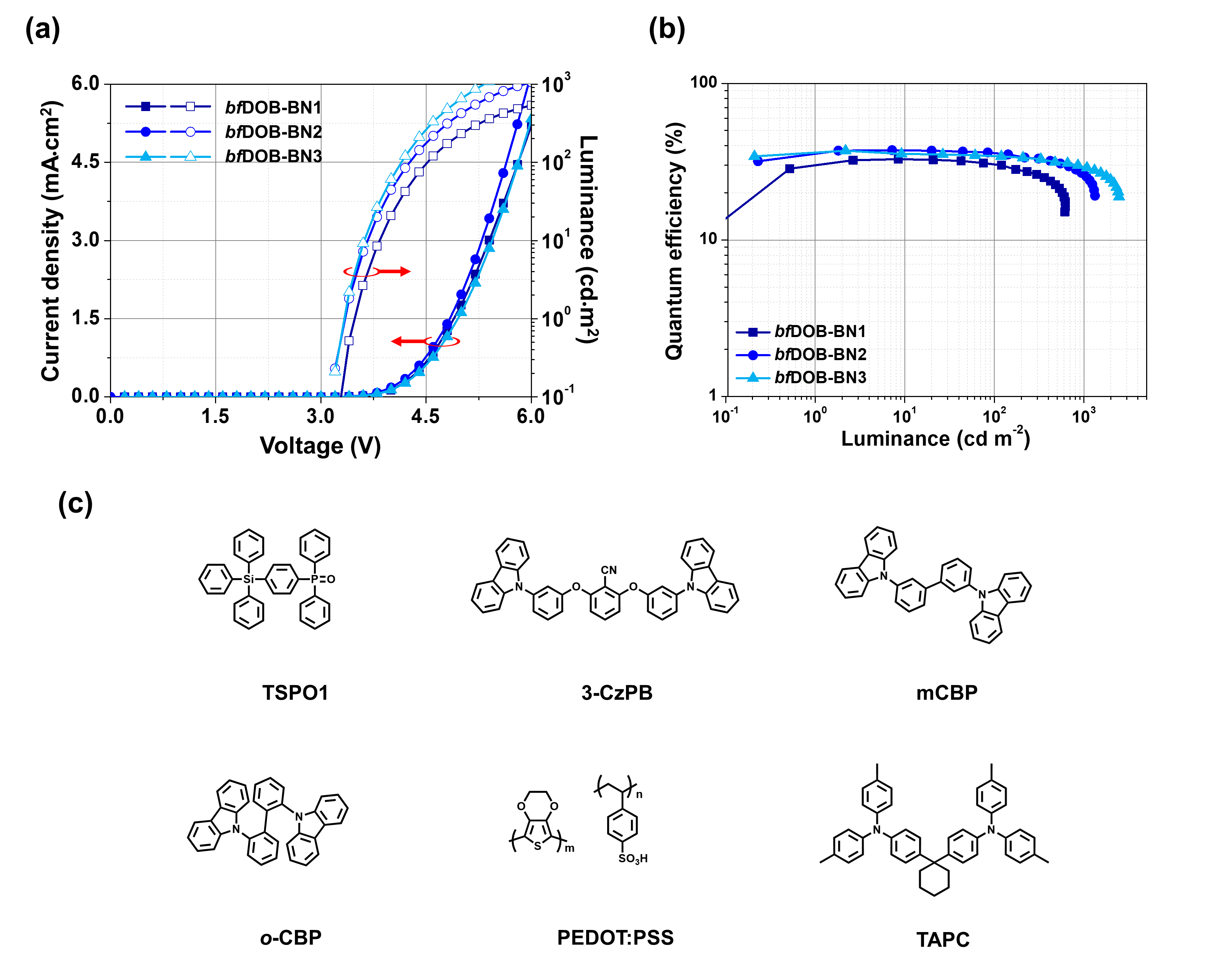


**Figure S33.** (a) Current density-voltage-luminance curves. (b) Quantum Efficiency-Luminance curves for ***bf*DOB-BN1**, ***bf*DOB-BN2** and ***bf*DOB-BN3**.


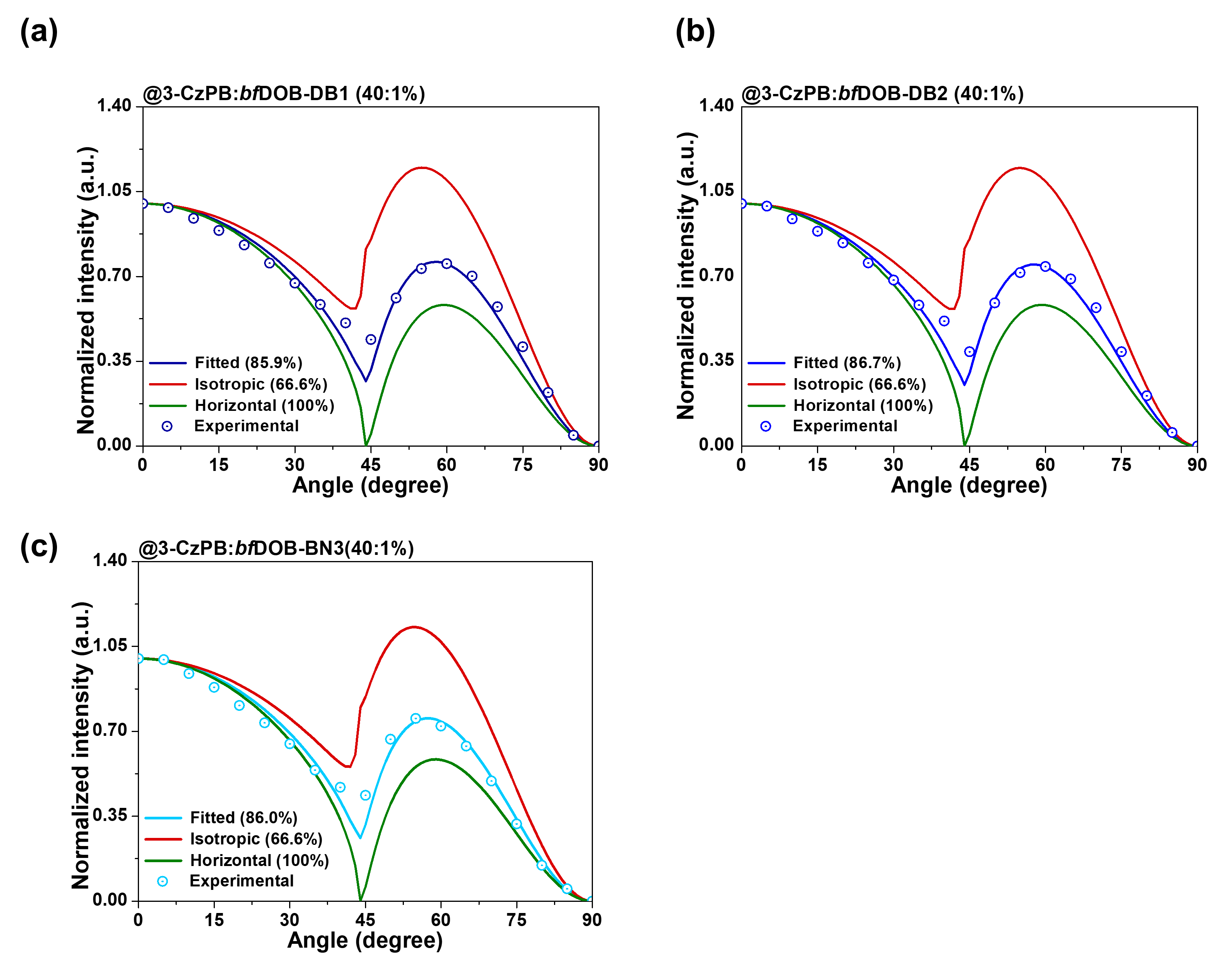


**Figure S34.** Angle-dependent PL spectra of (a) ***bf*DOB-BN1**, (b) ***bf*DOB-BN2** and (c) ***bf*DOB-BN3** doped at 1 wt% in the 3-CzPB host film.

**

**Figure S35.** Operational lifetime measurements of ***bf*DOB-BN1**, ***bf*DOB-BN2** and ***bf*DOB-BN3** at 100 cd/m^-2^.

**6. Supplementary Tables**

**Table S1.** The key parameters of ***bf*DOB-DB1**, ***bf*DOB-BN2**, and ***bf*DOB-BN3** compounds in the excited states.

| Emitters | S_1_  [eV] | T_1_  [eV] | T_2_  [eV] | ΔE(S_1_-T_1_) [eV] | ΔE(S_1_-T_2_)  [eV] | <S_1_\|H_soc_\|T_1_> | <S_1_\|H_soc_\|T_2_> | *k*_rISC_  [s^-1^] | *k_h_*_rISC_  [s^-1^] |
| --- | --- | --- | --- | --- | --- | --- | --- | --- | --- |
| *bf*DOB-BN1 | 2.917 | 2.784 | 2.830 | 0.133 | 0.087 | 0.053 | 0.138 | 1.017×10^4^ | 3.010×10^5^ |
| *bf*DOB-BN2 | 2.765 | 2.714 | 2.826 | 0.051 | -0.061 | 0.039 | 0.103 | 6.551×10^4^ | 3.775×10^6^ |
| *bf*DOB-BN3 | 2.685 | 2.650 | 2.759 | 0.035 | -0.074 | 0.085 | 0.104 | 4.602×10^5^ | 4.455×10^6^ |

**Table S2.** Solvent-dependent photophysical parameters used for the Lippert–Mataga analysis.

| Emitters | Solvent | ε | n | Δ*f* | λ_abs_  [nm] | λ_emi_  [nm] | $\tilde{\nu}$_abs_  [cm^-1^] | $\tilde{\nu}$_emi_  [cm^-1^] | Δ$\tilde{\nu}$ |
| --- | --- | --- | --- | --- | --- | --- | --- | --- | --- |
| *bf*DOB-BN1 | n-Hexane | 1.89 | 1.375 | -0.00008 | 432 | 435 | 23154 | 22989 | 165 |
|  | Cyclohexane | 2.02 | 1.426 | -0.001583 | 433 | 437 | 23105 | 22910 | 196 |
|  | Toluene | 2.38 | 1.496 | 0.0135043 | 435 | 443 | 22973 | 22599 | 374 |
|  | Diethyl ether | 4.34 | 1.352 | 0.1672196 | 434 | 443 | 23068 | 22599 | 469 |
|  | Tetrahydrofuran | 7.52 | 1.407 | 0.208946 | 436 | 448 | 22962 | 22321 | 641 |
|  | Dichloromethane | 8.93 | 1.424 | 0.2171701 | 437 | 457 | 22868 | 21882 | 986 |
|  | Acetonitrile | 35.94 | 1.344 | 0.3046093 | 435 | 459 | 22989 | 21786 | 1202 |
| *bf*DOB-BN2 | n-Hexane | 1.89 | 1.375 | -0.00008 | 438 | 442 | 22826 | 22650 | 176 |
|  | Cyclohexane | 2.02 | 1.426 | -0.001583 | 439 | 443 | 22758 | 22599 | 159 |
|  | Toluene | 2.38 | 1.496 | 0.0135043 | 441 | 447 | 22676 | 22371 | 304 |
|  | Diethyl ether | 4.34 | 1.352 | 0.1672196 | 440 | 454 | 22753 | 22026 | 727 |
|  | Tetrahydrofuran | 7.52 | 1.407 | 0.208946 | 441 | 468 | 22660 | 21368 | 1293 |
|  | Dichloromethane | 8.93 | 1.424 | 0.2171701 | 439 | 492 | 22779 | 20325 | 2454 |
|  | Acetonitrile | 35.94 | 1.344 | 0.3046093 | 440 | 514 | 22717 | 19474 | 3243 |
| *bf*DOB-BN3 | n-Hexane | 1.89 | 1.375 | -0.00008 | 448 | 450 | 22346 | 22222 | 124 |
|  | Cyclohexane | 2.02 | 1.426 | -0.001583 | 450 | 451.5 | 22247 | 22148 | 99 |
|  | Toluene | 2.38 | 1.496 | 0.0135043 | 449 | 465 | 22297 | 21505 | 791 |
|  | Diethyl ether | 4.34 | 1.352 | 0.1672196 | 447 | 479 | 22396 | 20877 | 1520 |
|  | Tetrahydrofuran | 7.52 | 1.407 | 0.208946 | 448 | 498 | 22346 | 20080 | 2266 |
|  | Dichloromethane | 8.93 | 1.424 | 0.2171701 | 451 | 529 | 22198 | 18904 | 3294 |
|  | Acetonitrile | 35.94 | 1.344 | 0.3046093 | 445 | 547 | 22472 | 18282 | 4190 |

**Table S3. Parameters extracted from the Lippert-Mataga plot.**

| Emitters | Regime | Slope [cm^-1^] | Intercept [cm^-1^] |
| --- | --- | --- | --- |
| *bf*DOB-BN1 | Single | 3138 | 190 |
| *bf*DOB-BN2 | Low polarity | 3189 | 199 |
|  | High polarity | 19873 | -2412 |
| *bf*DOB-BN3 | Single | 12120 | 180 |

**Table S4.** Calculated dipole moment between the S_0_ and S_1_ states of ***bf*DOB-BN1**, ***bf*DOB-BN2** and ***bf*DOB-BN3** based on DFT and TDDFT calculations.

| Emitters | Dipole moment | | |
| --- | --- | --- | --- |
|  | **S_0_** | **S_1_** | ΔS_0_-S_1_ |
| *bf*DOB-BN1 | 4.056 | 3.718 | 0.339 |
| *bf*DOB-BN2 | 4.551 | 3.971 | 0.580 |
| *bf*DOB-BN3 | 3.844 | 2.647 | 1.197 |

**Table S5. Comparison of experimental parameters from modified Arrhenius analysis and computational parameters from quantum-chemical calculations for *bf*DOB-BN1, *bf*DOB-BN2, and *bf*DOB-BN3.**

| Emitters | Experimental | | | | | Computational | | |
| --- | --- | --- | --- | --- | --- | --- | --- | --- |
|  | Slope | Intercept | ΔE_ST_ [eV] | *λ_eff_* [eV] | *H_SOC,eff_* [cm^-1^] | ΔE_ST_ [eV] | *λ* [eV] | *H_SOC_* [cm^-1^] |
| *bf*DOB-BN1 | -1260.20 | 18.77 | 0.08 | 0.2486 | 0.126 | 0.133 | 0.159 | 0.053 (S_1_↔T_1_) |
| *bf*DOB-BN2 | -1081.22 | 19.04 | 0.04 | 0.2871 | 0.149 | 0.051 | 0.160 | 0.103 (S_1_↔T_2_) |
| *bf*DOB-BN3 | -899.97 | 18.45 | 0.01 | 0.2899 | 0.112 | 0.035 | 0.160 | 0.104 (S_1_↔T_2_) |

**Table S6.** **Optical simulation results of *bf*DOB-BN2 incorporating the molecular orientation factor (86.7%).**

| Thickness of TSPO1 (nm) | Air (Out-coupling efficiency) | Substrate | Waveguide | SPP |
| --- | --- | --- | --- | --- |
| 15 | 0.26109 | 0.21154 | 0.14925 | 0.37811 |
| 16 | 0.26886 | 0.21398 | 0.15037 | 0.3668 |
| 17 | 0.27651 | 0.21629 | 0.15142 | 0.35578 |
| 18 | 0.28406 | 0.21849 | 0.15241 | 0.34505 |
| 19 | 0.29148 | 0.22057 | 0.15334 | 0.33462 |
| 20 | 0.29876 | 0.22254 | 0.15421 | 0.32448 |
| 21 | 0.3059 | 0.22442 | 0.15504 | 0.31465 |
| 22 | 0.31287 | 0.2262 | 0.15581 | 0.30512 |
| 23 | 0.31967 | 0.22789 | 0.15655 | 0.29589 |
| 24 | 0.32629 | 0.22951 | 0.15724 | 0.28697 |
| 25 | 0.33271 | 0.23105 | 0.1579 | 0.27834 |
| 26 | 0.33894 | 0.23253 | 0.15853 | 0.27001 |
| 27 | 0.34494 | 0.23395 | 0.15913 | 0.26198 |
| 28 | 0.35073 | 0.23533 | 0.15971 | 0.25423 |
| 29 | 0.35628 | 0.23667 | 0.16028 | 0.24678 |
| 30 | 0.36159 | 0.23798 | 0.16084 | 0.2396 |
| 31 | 0.36664 | 0.23926 | 0.16139 | 0.23271 |
| 32 | 0.37144 | 0.24054 | 0.16194 | 0.22608 |
| 33 | 0.37597 | 0.24181 | 0.1625 | 0.21973 |
| 34 | 0.38023 | 0.24308 | 0.16306 | 0.21363 |
| 35 | **0.3842** | 0.24436 | 0.16364 | 0.2078 |
| 37 | 0.39128 | 0.24699 | 0.16487 | 0.19686 |
| 39 | 0.39717 | 0.24976 | 0.16622 | 0.18686 |
| 41 | 0.40183 | 0.25271 | 0.16773 | 0.17774 |
| 43 | 0.40525 | 0.25588 | 0.16943 | 0.16944 |
| 45 | 0.40741 | 0.25932 | 0.17137 | 0.16191 |
| 47 | 0.4083 | 0.26304 | 0.17357 | 0.15508 |
| 49 | 0.40795 | 0.26709 | 0.17606 | 0.1489 |
| 51 | 0.40636 | 0.27146 | 0.17886 | 0.14332 |
| 53 | 0.40356 | 0.27616 | 0.18200 | 0.13828 |
| 55 | 0.39958 | 0.2812 | 0.18549 | 0.13373 |
| 57 | 0.39445 | 0.28656 | 0.18936 | 0.12964 |
| 59 | 0.3882 | 0.29223 | 0.19362 | 0.12594 |

**Table S7.** Summary of the reported deep-blue MR-TADF emitters based on single or double boron (CIE_y_≤0.1). ^[7]^

| Materials | EQE  [%] | CIE_y_ | *k*_rISC_  [10^4^ꞏs^-1^] | Reference |
| --- | --- | --- | --- | --- |
| ***bf*DOB-BN2** | **37.5** | **0.065** | **210** | **This work** |
| DABNA-1 | 13.5 | 0.090 | 0.99 | 7a |
| V-DABNA-F | 29.5 | 0.100 | 16 | 7b |
| PAB | 14.7 | 0.076 | 6.95 | 7c |
| 2tPAB | 16.8 | 0.076 | 6.34 |  |
| 3tPAB | 19.3 | 0.076 | 5.74 |  |
| BOBO-Z | 13.6 | 0.040 | 7 | 7d |
| BOBS-Z | 26.9 | 0.060 | 43 |  |
| BSBS-Z | 26.8 | 0.080 | 16 |  |
| CzBO | 13.4 | 0.050 | 0.9 | 7e |
| tDPAC-BN | 21.6 | 0.0940 | 1.16 | 7f |
| BIC-mCz | 19.4 | 0.050 | 0.4 | 7g |
| mDBIC | 13.5 | 0.050 | 0.5 |  |
| BN1 | 31.2 | 0.080 | 1.3 | 7h |
| BN3 | 37.6 | 0.080 | 25.5 |  |
| t-DABNA | 28.4 | 0.100 | 1.17 | 7i |
| t-DAB-DPA | 27.6 | 0.080 | 3.97 |  |
| 4F-ν-DABNA | 35.8 | 0.080 | 22.8 | 7j |
| 4F-m-ν-DABNA | 33.7 | 0.060 | 21 |  |
| DPACzBN2 | 24.0 | 0.100 | 2.9 | 7k |
| BFCz-DABNA | 28.0 | 0.09 | 2.78 | 7l |
| C-BN | 20.1 | 0.07 | 1.8 | 7m |
| TPD4PA | 30.7 | 0.06 | 25.1 | 7n |
| tBu-TPD4PA | 32.5 | 0.07 | 24.4 |  |
| DABNA-NP-TB | 7.03 | 0.076 | 1.4 | 7o |
| BSS-Cz | 21.8 | 0.09 | 15 | 7p |
| pBP-DABNA-Me | 23.4 | 0.092 | 6.85 | 7q |
| [B-N]N | 16.7 | 0.079 | 29 | 7r |
| α-3BNMes | 14.6 | 0.100 | 0.059 | 7s |
| m-DiNBO | 24.2 | 0.098 | 3.1 | 7t |
| OBN | 23.02 | 0.090 | 1.3 | 7u |
| NBN | 15.7 | 0.090 | 2.9 |  |
| ODBN | 24.5 | 0.1 | 2.1 |  |
| B-N-1 | 4.9 | 0.1 | 2.66 | 7v |
| BuDABNA | 25.1 | 0.093 | 0.66 | 7w |
| A-BN | 41.5 | 0.08 | 7.5 | 7x |
| ν-DABNA-Az1 | 30.8 | 0.083 | 23 | 7y |
| ν-DABNA-Az2 | 29.9 | 0.060 | 31 |  |
| ν-DABNA-Az3 | 33.0 | 0.100 | 34 |  |
| 5-Cz-BO | 22.8 | 0.046 | 27 | 7z |
| Py-BN | 15.8 | 0.045 | 0.64 | 7aa |
| Pm-BN | 5.8 | 0.045 | 0.42 |  |
| iPrAuBN | 14.8 | 0.036 | 83.4 | 7ab |
| ICz-BO | 12.0 | 0.031 | 1.66 | 7ac |
| 2FPAB | 4.2 | 0.044 | 2.46 | 7ad |
| MePAB | 19.8 | 0.046 | 2.37 |  |
| [B-N]N2 | 20.3 | 0.046 | 0.57 | 7ae |
| Me-PABO | 20.4 | 0.089 | 3.5 | 7af |
| IPrBN-mCP | 33.4 | 0.046 | 1.78 | 7ag |
| IPrBN | 20.8 | 0063 | 0.96 |  |
| DOBN | 35.4 | 0.04 | 7.5 | 7ah |
| DBNO | 27.2 | 0.042 | 2.88 | 7ai |
| DMBNO | 32.3 | 0.046 | 4.62 |  |
| 2B-DTACrs | 14.8 | 0.044 | 13 | 7aj |
| MeBN | 26.2 | 0.073 | 2.62 | 7ak |
| NaBN-Me | 32.3 | 0.069 | 4.66 |  |
| NaBN-DPA | 35.2 | 0.055 | 16.2 |  |
| m-Cz-DABNA | 6.94 | 0.07 | 0.6 | 7al |
| tBu-Cz-DABNA | 8.95 | 0.10 | 0.1 |  |
| BN-M1 | 18.7 | 0.049 | 7.09 | 7am |
| BN-M2 | 21.4 | 0.035 | 271 |  |
| BN-M3 | 34.8 | 0.045 | 119 |  |
| DPA-B2 | 28.9 | 0.055 | 8 | 7an |
| BN3 | 38.9 | 0.059 | 37 | 7ao |
| DBDS | 39.6 | 0.061 | 74 | 7ao |

**7. Reference**

[1] Y. Tsuchiya, S. Diesing, F. Bencheikh, Y. Wada, P. L. dos Santos, H. Kaji, E. Zysman-Colman, I. D. W. Samuel, C. Adachi, *J. Phys. Chem. A* **2021**, 125, 8074.

[2] a)K. Cheong, H. Lee, J. Moon, C. H. Ryu, G. W. Kim, J. Y. Kim, I. H. Lee, Y. W. Kim, Y. W. Lee, S. Yu, *Adv. Mater.* **2025**, 37, e10070; b)H. Lee, B. Park, G. R. Han, M. S. Mun, S. Kang, W. P. Hong, H. Y. Oh, T. Kim, *Adv. Mater.* **2024**, 36, 2409394; c)S. H. Jeon, S. Kang, T. Kim, *Adv. Theory Simul.* **2024**, 7, 2300363; d)S. Kang, T. Kim, *Heliyon* **2024**, 10; e)S. Kang, T. Kim, *Chem. Phys. Lett.* **2024**, 856, 141688.

[3] a)K. Shizu, H. Kaji, *Commun. Chem.* **2022**, 5, 53; b)A. Tajti, B. Kozma, P. G. Szalay, *J. Chem. Theory Comput.* **2020**, 17, 439; c)R. Berraud-Pache, F. Neese, G. Bistoni, R. Izsák, *J. Chem. Theory Comput.* **2019**, 16, 564; d)S. M. Pratik, V. Coropceanu, J.-L. Bredas, *ACS Mater. Lett.* **2022**, 4, 440; e)T. Izadkhast, M. Alipour, *J. Chem. Phys.* **2025**, 163.

[4] F. Neese, *WIREs Comput. Mol. Sci.* **2022**, 12, e1606.

[5] R. A. Marcus, in *Protein electron transfer*, Garland Science, 2020.

[6] A. F. Rausch, L. Murphy, J. G. Williams, H. Yersin, *Inorg. Chem.* **2012**, 51, 312.

[7] a)T. Hatakeyama, K. Shiren, K. Nakajima, S. Nomura, S. Nakatsuka, K. Kinoshita, J. Ni, Y. Ono, T. Ikuta, *Adv. Mater.* **2016**, 28, 2777; b)S. Oda, B. Kawakami, M. Horiuchi, Y. Yamasaki, R. Kawasumi, T. Hatakeyama, *Adv. Sci.* **2023**, 10, 2205070; c)Y. Wang, Y. Duan, R. Guo, S. Ye, K. Di, W. Zhang, S. Zhuang, L. Wang, *Org. Electron.* **2021**, 97, 106275; d)I. S. Park, M. Yang, H. Shibata, N. Amanokura, T. Yasuda, *Adv. Mater.* **2022**, 34, 2107951; e)I. S. Park, H. Min, T. Yasuda, *Angew. Chem. Int. Ed.* **2022**, 61, e202205684; f)Y. Wang, K. Di, Y. Duan, R. Guo, L. Lian, W. Zhang, L. Wang, *Chem. Eng. J.* **2022**, 431, 133221; g)X. Wang, Y. Zhang, H. Dai, G. Li, M. Liu, G. Meng, X. Zeng, T. Huang, L. Wang, Q. Peng, *Angew. Chem.* **2022**, 134, e202206916; h)X. Lv, J. Miao, M. Liu, Q. Peng, C. Zhong, Y. Hu, X. Cao, H. Wu, Y. Yang, C. Zhou, *Angew. Chem. Int. Ed.* **2022**, 61, e202201588; i)S. H. Han, J. H. Jeong, J. W. Yoo, J. Y. Lee, *J. Mater. Chem. C* **2019**, 7, 3082; j)K. R. Naveen, H. Lee, R. Braveenth, K. J. Yang, S. J. Hwang, J. H. Kwon, *Chem. Eng. J.* **2022**, 432, 134381; k)Y. Qiu, H. Xia, J. Miao, Z. Huang, N. Li, X. Cao, J. Han, C. Zhou, C. Zhong, C. Yang, *ACS Appl. Mater. Interfaces* **2021**, 13, 59035; l)H. Lee, R. Braveenth, J. D. Park, C. Y. Jeon, H. S. Lee, J. H. Kwon, *ACS Appl. Mater. Interfaces* **2022**, 14, 36927; m)T. Fan, Y. Zhang, L. Wang, Q. Wang, C. Yin, M. Du, X. Jia, G. Li, L. Duan, *Angew. Chem.* **2022**, 134, e202213585; n)K. R. Naveen, H. Lee, L. H. Seung, Y. H. Jung, C. K. Prabhu, S. Muruganantham, J. H. Kwon, *Chem. Eng. J.* **2023**, 451, 138498; o)S. Oda, W. Kumano, T. Hama, R. Kawasumi, K. Yoshiura, T. Hatakeyama, *Angew. Chem.* **2021**, 133, 2918; p)Y. Chang, Y. Wu, X. Wang, W. Li, Q. Yang, S. Wang, S. Shao, L. Wang, *Chem. Eng. J.* **2023**, 451, 138545; q)H. J. Cheon, S. J. Woo, S. H. Baek, J. H. Lee, Y. H. Kim, *Adv. Mater.* **2022**, 34, 2207416; r)G. Meng, H. Dai, J. Zhou, T. Huang, X. Zeng, Q. Wang, X. Wang, Y. Zhang, T. Fan, D. Yang, *Chem. Sci.* **2023**, 14, 979; s)K. Stavrou, S. Madayanad Suresh, D. Hall, A. Danos, N. A. Kukhta, A. M. Slawin, S. Warriner, D. Beljonne, Y. Olivier, A. Monkman, *Adv. Opt. Mater.* **2022**, 10, 2200688; t)G. Liu, H. Sasabe, K. Kumada, H. Arai, J. Kido, *Chem. Eur. J.* **2022**, 28, e202201605; u)J. Jin, C. Duan, H. Jiang, P. Tao, H. Xu, W. Y. Wong, *Angew. Chem. Int. Ed.* **2023**, 62, e202218947; v)K. Bai, M. Li, X. Tan, L. Dai, K. Liang, H. Li, S.-J. Su, *J. Mater. Chem. C* **2023**, 11, 16159; w)T. Lee, J. H. Jang, N. N. T. Nguyen, J. Jung, J. H. Lee, M. H. Lee, *Adv. Sci.* **2024**, 11, 2309016; x)G. Li, M. Du, T. Fan, X. Luo, L. Duan, Y. Zhang, *Mater. Today* **2024**, 73, 30; y)M. Mamada, A. Aoyama, R. Uchida, J. Ochi, S. Oda, Y. Kondo, M. Kondo, T. Hatakeyama, *Adv. Mater.* **2024**, 36, 2402905; z)R. Z. An, Y. Sun, H. Y. Chen, Y. Liu, A. Privitera, W. K. Myers, T. K. Ronson, A. J. Gillett, N. C. Greenham, L. S. Cui, *Adv. Mater.* **2024**, 36, 2313602; aa)X. Cai, Y. Pan, C. Li, L. Li, Y. Pu, Y. Wu, Y. Wang, *Angew. Chem.* **2024**, 136, e202408522; ab)X. F. Song, S. Luo, N. Li, X. Wan, J. Miao, Y. Zou, K. Li, C. Yang, *Angew. Chem.* **2025**, 137, e202413536; ac)K. Jiang, X. Chang, J. Zhu, T. Zhu, J. Yu, Y. Wang, Y. Zhang, D. Ma, W. Zhu, *Angew. Chem. Int. Ed.* **2025**, 64, e202421520; ad)H. Su, Y. Wang, K. Di, H. Yue, S. Huang, Y. Tian, Q. Zhang, H. Shao, R. Guo, L. Wang, *Adv. Funct. Mater.* **2025**, 35, 2419679; ae)D. Wan, J. Zhou, Y. Yang, G. Meng, D. Zhang, L. Duan, J. Ding, *Adv. Mater.* **2024**, 36, 2409706; af)K. Di, R. Guo, Y. Wang, Y. Lv, H. Su, Q. Zhang, B. Yang, L. Wang, *J. Mater. Chem. C* **2023**, 11, 6429; ag)M. Xing, G. Chen, S. Wang, X. Yin, J. Liu, Z. Xue, N. Li, J. Miao, Z. Huang, C. Yang, *Adv. Funct. Mater.* **2025**, 35, 2414635; ah)J. Jin, M. Chen, H. Jiang, B. Zhang, Z. Xie, W.-Y. Wong, *ACS Mater. Lett.* **2024**, 6, 3246; ai)Z. Xue, Z. Xiao, Y. Zou, Z. Chen, J. Liu, Z. Huang, C. Yang, *Chem. Sci.* **2025**, 16, 3655; aj)C.-Y. Chan, S. Madayanad Suresh, Y.-T. Lee, Y. Tsuchiya, T. Matulaitis, D. Hall, A. M. Z. Slawin, S. Warriner, D. Beljonne, Y. Olivier, C. Adachi, E. Zysman-Colman, *Chem. Commun.* **2022**, 58, 9377; ak)J. Liang, J. Dong, S. Xian, N. Li, J. Liu, C. Zhong, Z. Chen, C. Yang, Z. Huang, *Chem. Eng. J.* **2025**, 522, 167524; al)M. Tirupati, J. H. Ham, S. Muruganantham, S. C. Cha, Y. H. Jung, J. H. Kwon, *Angew. Chem. Int. Ed.* **2025**, 64, e202510190; am)Y. Wang, X. Guo, J. Bian, Z. Ma, X. Ge, L. Jiang, G. Li, Z. Chen, D. Guo, J. Zhao, Z. Yang, J. Miao, Z. Chi, *Adv. Mater.*, n/a, e14617; an)T. Hua, X. Cao, J. Miao, X. Yin, Z. Chen, Z. Huang, C. Yang, *Nat. Photonics* **2024**, 18, 1161; ao)H. Lin, Z. Ye, S. Xian, Z. Chen, J. Miao, Z. Huang, C. Zhong, S. Gong, X. Cao, C. Yang, *Adv. Mater.* **2025**, 37, 2502459.
